# Identifying temporal molecular signatures underlying cardiovascular diseases: A data science platform

**DOI:** 10.1016/j.yjmcc.2020.05.020

**Published:** 2020-06-03

**Authors:** Neo Christopher Chung, Howard Choi, Ding Wang, Bilal Mirza, Alexander R. Pelletier, Dibakar Sigdel, Wei Wang, Peipei Ping

**Affiliations:** a NHLBI Integrated Cardiovascular Data Science Training Program at University of California (UCLA), Los Angeles, USA; b Departments of Physiology and Medicine (Cardiology) at UCLA School of Medicine, USA; c Institute of Informatics, Faculty of Mathematics, Informatics and Mechanics University of Warsaw, Warsaw, Poland; d Bioinformatics and Medical Informatics at UCLA School of Engineering, Los Angeles, CA 90095, USA; e Scalable Analytics Institute (ScAi) at UCLA School of Engineering, Los Angeles, CA 90095, USA

**Keywords:** Temporal molecular signatures, Unsupervised clustering, Proteomics, Time-course, Data science, Oxidative post-translational modification

## Abstract

**Objective:**

During cardiovascular disease progression, molecular systems of myocardium (e.g., a proteome) undergo diverse and distinct changes. Dynamic, temporally-regulated alterations of individual molecules underlie the collective response of the heart to pathological drivers and the ultimate development of pathogenesis. Advances in high-throughput omics technologies have enabled cost-effective, temporal profiling of targeted systems in animal models of human diseases. However, computational analysis of temporal patterns from omics data remains challenging. In particular, bioinformatic pipelines involving unsupervised statistical approaches to support cardiovascular investigations are lacking, which hinders one’s ability to extract biomedical insights from these complex datasets.

**Approach and results:**

We developed a non-parametric data analysis platform to resolve computational challenges unique to temporal omics datasets. Our platform consists of three modules. Module I preprocesses the temporal data using either cubic splines or principal component analysis (PCA), and it simultaneously accomplishes the tasks on missing data imputation and denoising. Module II performs an unsupervised classification by K-means or hierarchical clustering. Module III evaluates and identifies biological entities (e.g., molecular events) that exhibit strong associations to specific temporal patterns. The jackstraw method for cluster membership has been applied to estimate *p*-values and posterior inclusion probabilities (PIPs), both of which guided feature selection. To demonstrate the utility of the analysis platform, we employed a temporal proteomics dataset that captured the proteome-wide dynamics of oxidative stress induced post-translational modifications (O-PTMs) in mouse hearts undergoing isoproterenol (ISO)-induced hypertrophy.

**Conclusion:**

We have created a platform, CV.Signature.TCP, to identify distinct temporal clusters in omics datasets. We presented a cardiovascular use case to demonstrate its utility in unveiling biological insights underlying O-PTM regulations in cardiac remodeling.

## 1. Introduction

Pathological progression of chronic diseases often involves dynamic changes of a vast collection of molecular events, including multi-factorial alterations across organ functions and biological processes (e.g., genome, proteome, and metabolome) [1,2]. Systematic characterization of these molecular profiles in their temporal fashion corresponding to disease progress is seen as essential to our understanding on mechanisms and pathogenesis of disease progression [3,4].

Over the past two decades, advances in omics technologies have enabled researchers to cost-effectively obtain large scale data in time-series. One major challenge is how to decode these massive molecular events and their association with disease in a phenotypically meaningful fashion. Classical statistical methods for conventional biological studies have shown success in targeting static conditions in a snap-shot view; however, they are not suitable for omics investigations tracking disease progression. Temporal datasets are often difficult to analyze in an unsupervised manner, due to their inherited complexity, sparsity, and noise levels.

Biomedical innovation and discovery have been supported by two major driving forces: the classical investigative hypothesis-centric approach relies heavily on previously published results; and the recent development of data-driven methods focuses on the understanding of data with the aid of computational intelligence. The success of the latter requires a few notable technical considerations. First, longitudinal datasets (e.g., proteomics) of complex diseases often contain missing values and are embedded with noise. Second, molecular signatures indicative of pathogenesis and phenotypes are often unknown priori, thus requiring advanced computation and extraction. Third, validation of computational output (e.g., molecular signatures) will benefit from an unbiased approach. Finally, an open source package is necessary to enable a straightforward implementation of the protocol, providing a modular system for examination and improvement of each step. Accordingly, we have developed the Cardiovascular Signature Temporal Clustering Platform (CV.Signature.TCP), a data science package tailored for longitudinal proteomics studies to extract temporal molecular patterns indicative of disease phenotypes.

As a use case scenario, we employed a previously published proteomics dataset in cardiovascular biology [5]. The temporal changes of cysteine O-PTMs across the myocardial proteome were captured over time using a mouse model of cardiac hypertrophy [5]. Biological variables included in each O-PTM are defined by its host protein, modification type, modification site, and occupancy. We applied CV.Signature.TCP to analyze this dataset in an unsupervised and non-parametric fashion; our tool identified O-PTM subgroups of temporal importance and enabled further functional delineation. Both the parameter settings and analytical routes of CV.Signature.TCP are general-izable to allow a broader adaptation to other temporal omics data.

## 2. Methods

**Fig. 1.**
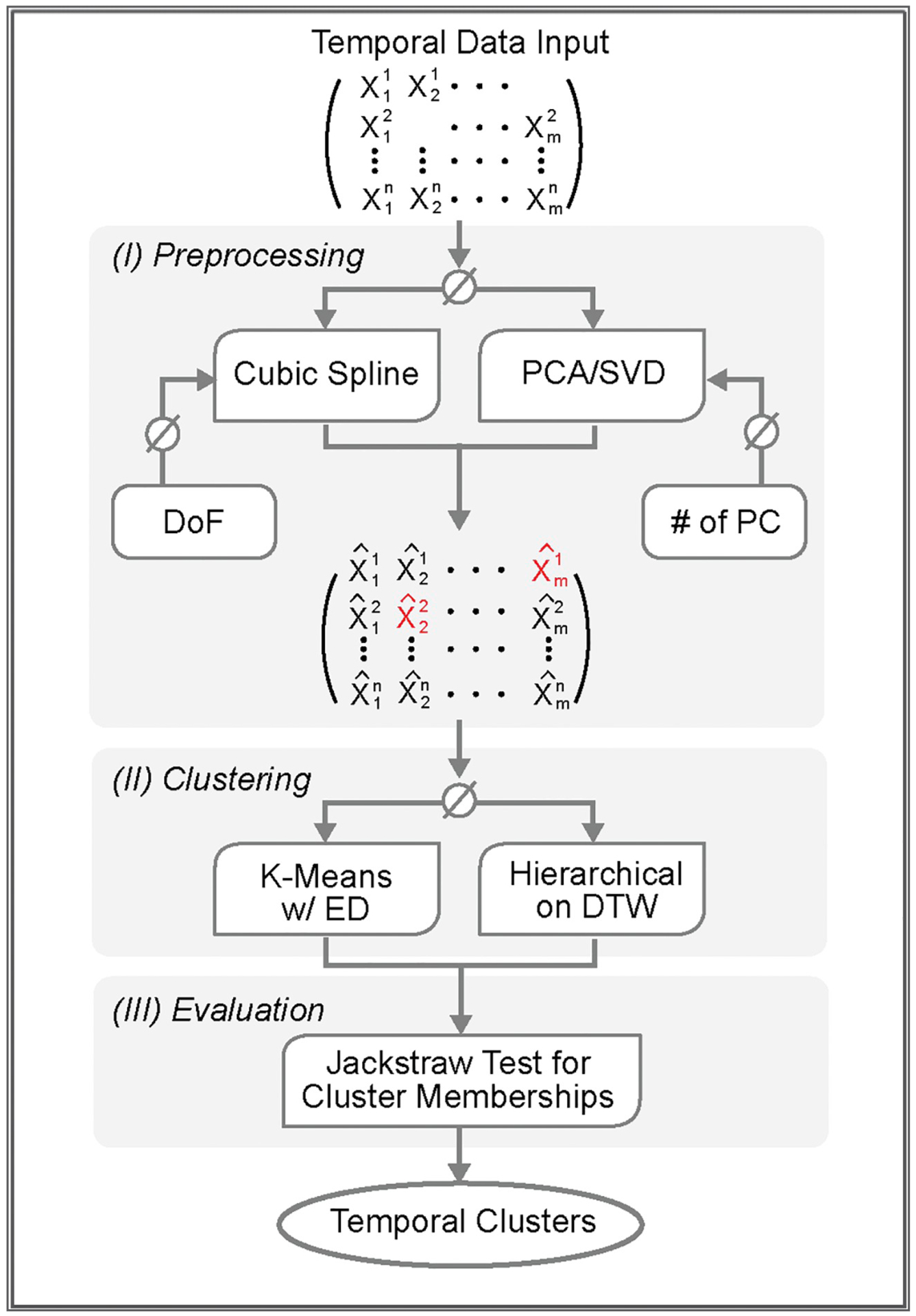
Schematic Overview of CV.Signature.TCP. A computational platform CV.Signature.TCP has been developed to discover temporal patterns of biological molecules related to the progression of diseases (e.g., cardiac hypertrophy). The temporal omics dataset is processed by 3 modules: (I) *Preprocessing*, (II) *Clustering*, and (III) *Evaluation*. Module I conducts missing data imputation and denoising simultaneously via cubic spline. Alternatively, principal component analysis (PCA) and singular value decomposition (SVD) are used. Module II identifies major temporal patterns using K-means with Euclidean distance (ED) and hierarchical clustering with dynamic time warping (DTW). Module III evaluates the significance of molecular variables (e.g., protein O-PTMs) in their clusters using the jackstraw test to obtain *p-*values and posterior inclusion probabilities (PIPs).

Major technical considerations for analyzing temporal proteomics data include missing value imputation, denoising, clustering, and evaluation of variables. Accordingly, we designed and selected non-parametric methods in order to avoid strong assumptions. CV.Signature.TCP has three functional modules, I) *Preprocessing*, II) *Clustering*, and III) *Evaluation* (Fig. 1). This platform is implemented in an open source R package, CV.Signature.TCP (https://github.com/UCLA-BD2K/CV.Signature.TCP).

Considering temporal proteomics datasets with *m* variables (rows) and *n* samples (columns), which correspond to *n* time points. It may be necessary to remove some variables that exhibit minimal or noisy temporal dynamics, utilizing fold changes or dispersion statistics. In the *Preprocessing* module which conducts missing data imputation and denoising, we provide two independent non-parametric methods, cubic splines [6,7] and principal component analysis (PCA) [8]. Cubic splines, which require a minimum of 4 available data points per variable, use inherent temporal structure to impute missing values and reduce the temporal noise simultaneously. Smoothing parameters can be automatically chosen by the platform via cross validation which minimizes the test error of predictive models. Alternatively, the reduced rank model uses the overall systematic variation captured by PCA to denoise the data. After applying PCA, the r < min(m, n) PCs and their loadings are used to reconstruct the data. If values are missing, SVDImpute [9] or nonlinear Iterative Partial Least Squares (NIPALS) [10] are employed. The results of preprocessing are compared with the original data by Pearson correlation statistics and mean squared differences (see details in Supplemental Information).

With the preprocessed data, the platform groups biological variables that covary over time in an unsupervised manner. In the *Clustering* module, two independent clustering models, K-means or hierarchical clustering, are available. Note that temporal clustering is still an actively evolving field. We compared popular software packages that facilitate omics data analyses; details are included in Supplemental Information. When sampling rates or dynamics differ among measurements (e.g., longitudinal clinical data), it might be necessary to apply dynamic time warping (DTW). In the *Evaluation* module, examination of molecular variables in their association with respective clusters is conducted. The jackstraw method is performed to overcome the inherent circular dependency of conducting association tests when the clusters are extracted from omics data. Further, jackstraw test for cluster membership provides *p*-values and posterior inclusion probabilities (PIPs) for individual variables [11,12]. PIPs, which are directly related to local FDRs, are then applied in feature selection and downstream analyses [12,13].

To demonstrate utilities of the CV.Signature.TCP application, we show a sample analysis on a temporal dataset of protein cysteine O-PTMs amid cardiac remodeling [5]. Briefly, the temporal changes in 3 types of cysteine O-PTMs (reversible cysteine O-PTMs; irreversible CysSO_2_H and CysSO_3_H) at the proteome level were obtained using a mouse model of cardiac hypertrophy [5]. These proteomic datasets consist of 6 time points (1, 3, 5, 7, 10, 14 days with ISO treatment) and multiple variables, including modification site/occurrence, modification type, and modification occupancy of cysteine O-PTM on cardiac proteins. The ratio of occupancy (ISO over Control) was calculated for individual protein O-PTMs and averaged among 4 replicates, then followed by a log transformation. Protein O-PTMs alterations are filtered based on criteria established in proteomic studies [13,14]. All O-PTMs exhibiting significant temporal responses during ISO treatment were examined using CV.Signature.TCP. Their associated biological functions (BFs) were annotated using information retrieved from Reactome (release V71, 2019_Dec; https://reactome.org/) [15] and UniProt knowledgebases [16].

## 3. Results and discussion

### 3.1. Assembly a complete data matrix for temporal analysis via CV.Signature.TCP

Our CV.Signature.TCP employs two non-parametric methods, tackling the missing data imputation and denoising simultaneously via the *Preprocessing* module (Fig. 1). We chose to apply the method of cubic splines, in which a degree of freedom (DoF) is automatically selected by global cross validation (dof = “cv.global” option in the preprocessing_spline function). Note that for 165 O-PTMs with only 3 observations (e.g., data points), a missing value imputation based on PCA/SVD is applied. After cubic splines are calculated, the predicted values are obtained for all time points. We validated the Preprocessing module to ensure accuracy and reliability. In particular, we have observed high Pearson correlation (a median coefficient of 0.97) between the input data and preprocessed data (Supplemental Fig. S1A). Furthermore, mean squared differences (MSDs) are low with a median of 0.28 (Supplemental Fig. S1B).

We subsequently applied the CV.Signature.TCP to the cardiac O-PTM datasets. In this dataset, a total of 3446 oxidized cysteine protein O-PTMs were identified, including reversible cysteine O-PTMs, cysteine sulfinylation (CysSO_2_H), and cysteine sulfonylation (CysSO_3_H). Among them, 1735 exhibited temporal alterations; when we applied a threshold of 1.2 Fold-change to filter O-PTMs with limited dynamic changes, we obtained a total number of 1605 O-PTMs underwent temporal alterations (see Supplemental Table S1).

### 3.2. Extraction of temporal patterns using CV.Signature.TCP

**Fig. 2.**
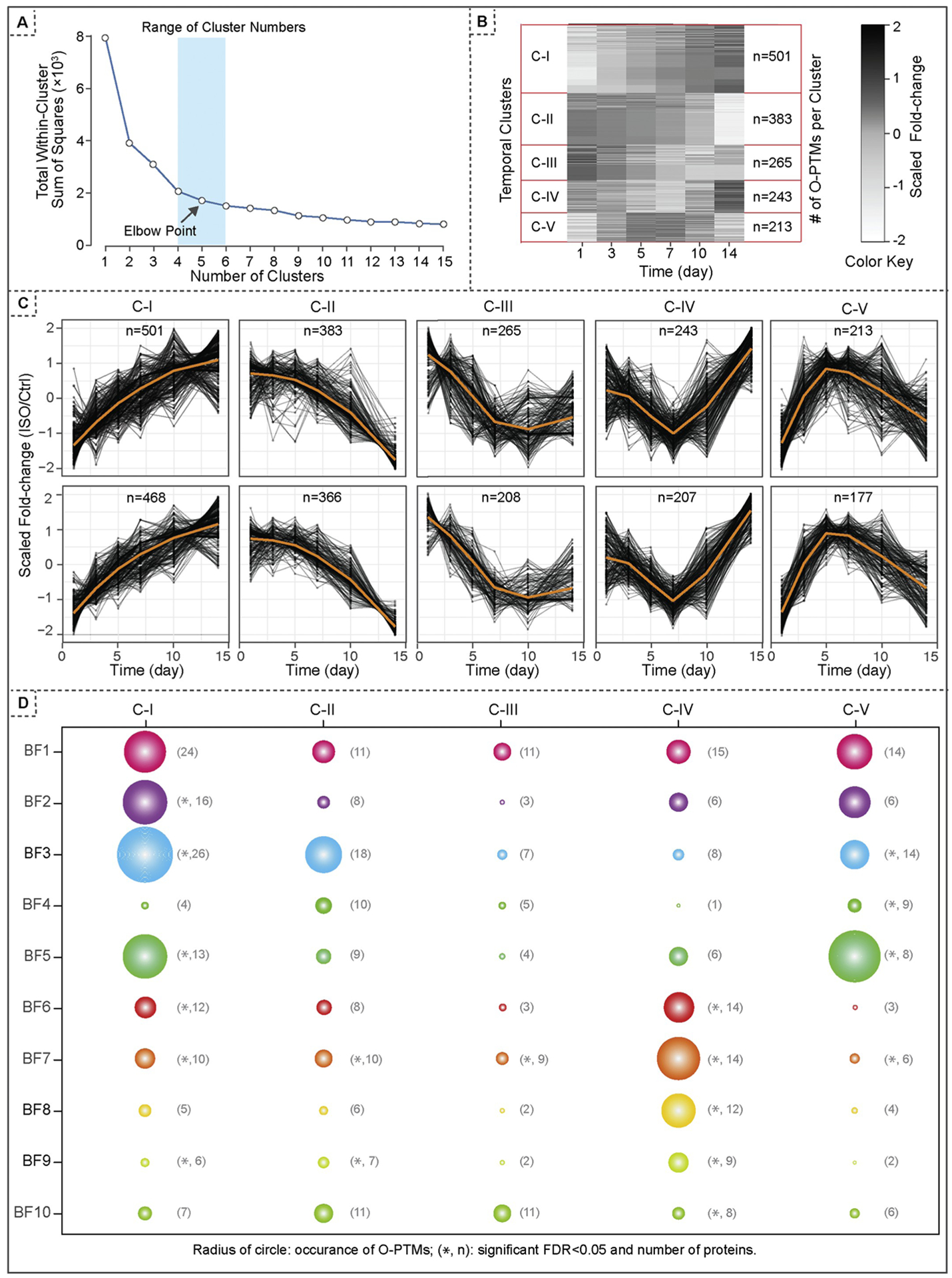
Analysis of Cysteine O-PTMs using CV.Signature.TCP. (A) The scree plot was used to determine a range for the possible number of clusters (K = 4–6). By comparing the clustering results using these K-values, the optimal number of clusters were determined (K = 5) to sufficiently capture the dynamics of cysteine O-PTMs during cardiac remodeling. (B) CV.Signature.TCP platform was employed to extract 5 unique temporal patterns across 1605 Cysteine O-PTMs. A heatmap was used to visualize the temporal changes of O-PTM occupancy for individual O-PTMs. (C) Cysteine O-PTMs in mice vary over time in response to cardiac remodeling. We applied cubic splines with cross-validation to impute and denoise 1605 cysteine O-PTMs. K-means clustering identified 5 clusters (*top row*). Then, the jackstraw test for cluster memberships was applied and 1426 O-PTMs with PIP > 80% were selected (*bottom row*). (D) Protein O-PTMs of temporal significance were further annotated by their temporal patterns (as shown in five clusters) and their biological functions (BFs as shown in 10 essential pathways). Each circle represents a cluster of O-PTMs sharing both the temporal pattern and BF attribute. The occurrences of O-PTMs (a radius of a circle), the false discovery rate (*, FDR < 0.05), and the number of proteins (n) are labelled for each O-PTM cluster. BF1, neutrophil degranulation; BF2, response to elevated platelet cytosolic Ca^2+^; BF3, extracellular matrix organization; BF4, protein translation; BF5, post-translational protein phosphorylation; BF6, glucose metabolism; BF7, pyruvate metabolism and citric acid (TCA) cycle; BF 8, respiratory electron transport; BF9, branched-chain amino acid (BCAA) catabolism; BF10, fatty acid metabolism.

The *Clustering* module of CV.Signature.TCP subsequently employed K-means clustering to extract unique O-PTM temporal patterns during cardiac remodeling (Fig. 1). The scree plot of total within-cluster sum of squares (WCSS) was used to determine a range of possible number of clusters (K = 4–6, Fig. 2A). After comparing the clustering results with these K values, we determined the optimal K value as 5 which resulted in the most distinct temporal patterns. Accordingly, these 1605 O-PTMs were classified into 5 clusters based on the temporal changes in their cysteine O-PTM occupancy (Fig. 2B). Cluster I (C-I) is characterized by a continual descend; cluster II (C-II) by a continual ascend; cluster III (C-III) by an initial descend in the first 7 days followed by remaining at the lowest level; cluster IV (C-IV) by a slight descend in the first 7 days followed by an accelerated increase; and cluster V (C-V) by an initial ascend in first 5–7 days followed by a relapse to the original level (Fig. 2C, top row).

For the *Evaluation* module, we performed a jackstraw test to examine the cluster membership (Fig. 1), computing the individual *p-*values for these 1605 O-PTMs. These *p*-values were further used to estimate the posterior inclusion probabilities (PIPs) [11,12]. Essentially, by estimating the null distribution of F-statistics under independence, we evaluate whether the variables in a given cluster are correctly assigned. The jackstraw procedure learns the over-fitting characteristics of unsupervised clustering and identifies variables that are included in a cluster by a randomized fashion. This step naturally provides potential molecular signature validation based on PIPs. Here we applied a PIP threshold *>* 0.8 to retain 1426 O-PTMs that are strongly related to individual clusters. The *Evaluation* module filtered out biological variables with limited or noisy contribution to the major trend, identifying O-PTMs with significant dynamics during disease progression (Fig. 2C, bottom row).

### 3.3. Exploring biological insights subsequent to analyses by CV.Signature.TCP

Post completion of the cluster analyses, we conducted functional annotation on all five clusters containing the 1426 O-PTMs, retrieving 10 essential biological functions (BFs) using both Reactome [15] and Uniprot knowledgebases [16], The relevant BFs are “inflammation response” (BF1), “calcium signaling” (BF2), “extracellular matrix (ECM) remodeling” (BF3), “protein translation and post-translational regulation” (BFs 4&5), and “energy production and metabolism” (BF6-BF10).The O-PTMs associated with each BF were further classified with their temporal patterns under oxidative stress (Fig. 2D). Accordingly, the occurrences of O-PTMs (radius of circles), false discovery rate (* denotes FDR < 0.05), and the number of proteins (n) are presented for each group, enabling further investigation on dynamic oxidative stress regulations among function-related proteins (Fig. 2D and Supplemental Table S2).

These analyses correlate biological functional significance of molecular variables, in our user case, the O-PTMs, to their displayed temporal pattern. For example, cluster I (C-I) is affiliated with all 10 distinct biological function (BF) groups. The type and frequency of Cysteine O-PTMs are highly dynamic across these BF groups, with “ECM remodeling (BF3)” displaying the highest O-PTM events (43 O-PTMs, shown as the radius of the circle) and functional representation (i.e., FDR < 0.05) hosted by 26 proteins. The other 4 clusters (C-II to C-V) also display a varied degree of associations with these 10 BFs. In C-II, BF3 has the highest O-PTM frequency, whereas both “pyruvate metabolism and TCA cycle (BF7)” and “branched chain amino acid catabolism (BF9)” exhibit highest functional representation. Cluster III (C-III) showed the least protein O-PTM events in all 10 BFs combined, with only one notable functional representation in BF7. In cluster IV (C-IV), the BFs related to “energy production and metabolism (BFs 6–10)” are all highly represented. Finally for cluster V (C-V), “post-translational protein phosphorylation” stands out among others with the highest frequency and functional presentation. Taken together, these temporal changes defined individual protein O-PTM events under pathological stimuli, unveiling novel regulatory targets for intervention and/or potential candidates for biomarkers.

## 4. Conclusion

Our data science platform is developed to enable unsupervised characterization of temporal patterns underlying any disease progression. Implemented in an open source R package ‘CV.Signature.TCP’, it is applicable to a wide range of temporal molecular datasets.

## Supplementary Material

Supplementary Data

## Abbreviations:

BF: biological function
CVDs: cardiovascular diseases
DoF: degree of freedom
DTW: dynamic time warping
ED: Euclidean distance
FDR: false discovery rate
ISO: Isoproterenol
O-PTMs: oxidative stress induced post-translational modifications
PCA: principal component analysis
PIPs: posterior inclusion probabilities
SVD: singular value decomposition
CV.Signature.TCP: cardiovascular signature temporal clustering platform
