# Supplementary material for: Identifying temporal molecular signatures underlying cardiovascular diseases: A data science platform
Source: J Mol Cell Cardiol. Author manuscript; Available in PMC 2021 Aug 1. (PMC7583079; doi:10.1016/j.yjmcc.2020.05.020)
Supplement: Supplementary Data [file NIHMS1637030-supplement-Supplementary_Data.pdf]

SUPPLEMENTAL INFORMATION

Identifying Temporal Molecular Signatures Underlying Cardiovascular Diseases: A Data Science Platform

Neo Christopher Chung[1,2,3,*], Howard Choi[1,2,4,5], Ding Wang[2], Bilal Mirza[2], Alexander R. Pelletier[1,4,5], Dibakar Sigdel[1,2], Wei Wang[1,4,5], Peipei Ping[1,2,4,5,*]

[1]NHLBI Integrated Cardiovascular Data Science Training Program at University of California, Los Angeles (UCLA), [2]Departments of Physiology and Medicine (Cardiology) at UCLA School of Medicine, [3]Institute of Informatics, Faculty of Mathematics, Informatics and Mechanics University of Warsaw, Warsaw, Poland, [4]Bioinformatics and Medical Informatics, and [5]Scalable Analytics Institute (ScAi) at UCLA School of Engineering, Los Angeles, CA, 90095, USA

Preprocessing Assessment

Preprocessing helps the users to impute and denoise temporal proteomics data. It is important to understand how the choices of methods and parameters impact data analysis. While comprehensive comparison is beyond the scope of this paper, we have used two assessment approaches to ensure reliability. First, we calculated Pearson correlation statistics between original observed O-PTM values and their corresponding preprocessed values. Then, these 1605 correlation statistics are shown in **Supplemental Figure S1A**. Most users may likely be interested in ensuring that such correlation remains high. In O-PTM data, the correlation coefficients are strongly skewed towards 1, with a median of 0.97. Second, similarly we calculated the mean squared differences (MSD) between original and preprocessed data. As shown in **Supplemental Figure S1B**, The median MSD is 0.28 and 76.8% of O-PTMs have MDS < 1. Unlike correlation coefficients which are bounded by [-1,1], MSDs depend on magnitudes. Note that extremely low MSDs do not necessarily mean superior performance; not applying any denoising would result in MSD = 0. In the future, it may be beneficial to utilize preprocessing assessments such as correlation and MSD to filter out problematic variables in the downstream analyses.

Selecting the Number of Clusters

A key hyperparameter in identifying major temporal patterns in an unsupervised setting is the number of clusters. As one of the most popular methods, the Elbow method creates the scree plot of the total within-cluster sum of squares (WCSS). Generally, one would like to approximate the point of inflection (elbow point) in WCSS. Our analysis indicates the point of inflection around 5 (**Fig. 2A**). Alternatively, the silhouette analysis measures within-cluster cohesion and between-cluster separation [9]. Ranging from -1 to 1, the high values indicate better clustering where a variable is confidently assigned to its own cluster. In **Supplemental Figure S2**, the average silhouette values are shown; the maximum value occurs at 2. However, using K of 2 is the most coarse option, splitting O-PTMs into two large groups (**Supplemental Figure S3**). It appears when K is set to 5, we could get the most distinct representatives of temporal patterns. We recommend the investigators to test a range of K values based on visualization, evaluation metric, and data analysis.

Alternate Choices in Omics Analysis Software Packages

There exists a wide range of software packages that facilitate omics data analyses. Here we provide comparisons of their functional specialties, which may help investigators to choose for

their own datasets. Generally, identifying major patterns in large-scale data is an active area of research, and time-series clustering remains a challenge [1,2]. Thus, there are several popular software packages that aim to accomplish clustering temporal proteomics data.

TSClust written in R by Drs. Montero and Vilaris (University of A Coruña) implements various similarity measures and time-series clustering algorithms [3]. This includes and extends K-means clustering, hierarchical clustering, and dynamic time warping [4]. It may be very useful to compare different model-free and model-based algorithms and their options available in TSClust. However, it doesn't provide an end-to-end pipeline, such as preprocessing and evaluation, and is not tailored to proteomics or cardiovascular research. CV.Signature.TCP is designed to be modular and extensible such that other clustering algorithms can be incorporated when appropriate.

MetaboAnalyst developed by Xia Lab (McGill University) and Wishart Lab (University of Alberta) offers a web-based platform with a variety of commonly used procedures for data processing, multivariate statistical analysis and data annotation [5]. There are some similarities between MetaboAnalyst and our platform, such as SVD/PCA for missing value imputation and k-means clustering. In contrast, we focus on identifying molecular signatures from temporal proteomics. CV.Signature.TCP performs data preprocessing prior to the Clustering module, which reduces noise from temporal proteomics data, allowing essential patterns of the variables to stand out. Moreover, CV.Signature.TCP offers the Evaluation module to identify biological variables with significant temporal patterns.

mixOmics is an R package composed by Prof. Lê Cao and colleagues (University of Melbourne) for multivariate models of omics data, integrating a mixture of omics data [6]. Analysis methods available in mixOmics mostly rely on existing phenotypes. With respect to phenotypes or other independent variables, mixOmics identifies important variables such as proteins. In contrast, CV.Signature.TCP enables an unsupervised clustering and evaluation, and may be helpful when independent variables are inaccurate or unavailable. Note that both mixOmics and CV.Signature.TCP allow missing data imputation using the non-linear Iterative Partial Least Squares (NIPALS) algorithm [7]. Particularly, in Preprocessing module, PCA/SVD must be approximated if there are missing values, where NIPALS [7] and SVDImpute [8] are available for missing data imputation.

Temporal and Functional Annotation of O-PTMs

We conducted the temporal and functional annotation of O-PTMs. Our goal is not only to perform pathway enrichment on the protein hosts of O-PTMs, but also to uncover whether there are potential links between the temporal behavior of an O-PTM and its functional attribute. Functional annotation often implies the spatial proximity (e.g., ETC complex proteins), subcellular localization (e.g., ECM proteins), and sequence homology (e.g, contractile proteins) among members belonging to the same pathway. We input the protein hosts of O-PTMs exhibiting the same temporal patterns (i.e., separately for members in Clusters I-V) into the analysis pipeline using the Reactome database (www.reactome.org) and the over-representation of pathways was statistically measured using hypergeometric test followed by multiple testing correction (i.e., Benjamini-Hochberg method). The false discovery rates (FDR) were then used to map the high-score functional pathways back to the proteins associated with individual temporal clusters. We first selected 36 pathways that highly enriched with O-PTMs with temporal significance and then prioritized 10 pathways carrying significant biological functions (**Fig. 2D**). Within each biological functional group (BFs) of proteins, their O-PTMs are categorized into 5 clusters based on their temporal behaviors under oxidative regulation, which are defined by 3 critical parameters (i.e., the number of O-PTMs, FDR, and number of protein hosts).

SUPPLEMENTAL FIGURES

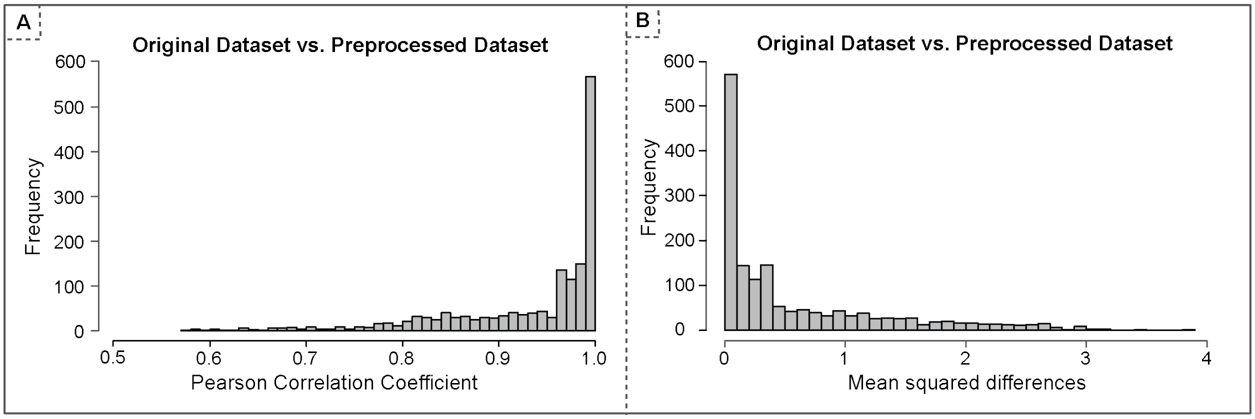

Supplemental Figure S1. Preprocessing assessment. We calculate Pearson correlation statistics and mean squared differences (MSD) between the original O-PTM values and corresponding preprocessed values. The medians of correlation and MSD are 0.97 and 0.28, respectively.

(Two-column Figure, Black & White Figure)

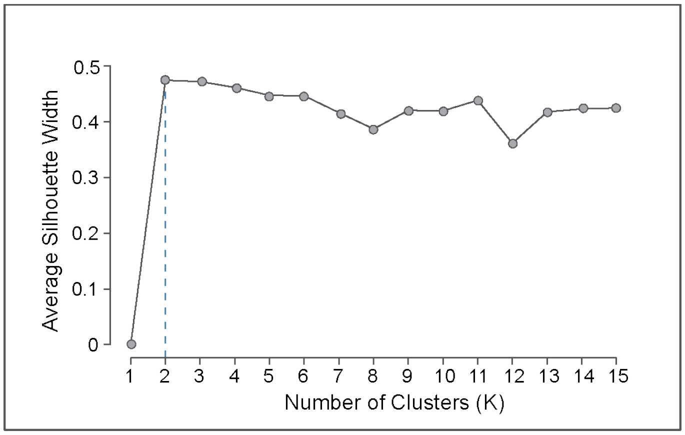

Supplemental Figure S2. Silhouette analysis of O-PTM data. In addition to the scree plot of the total within-cluster sum of squares (Figure 2(a)), the silhouette plot may be used to help select the number of clusters. In O-PTM data, the maximum average silhouette width occurs at K=2.

(One-column Figure, Color Figure)

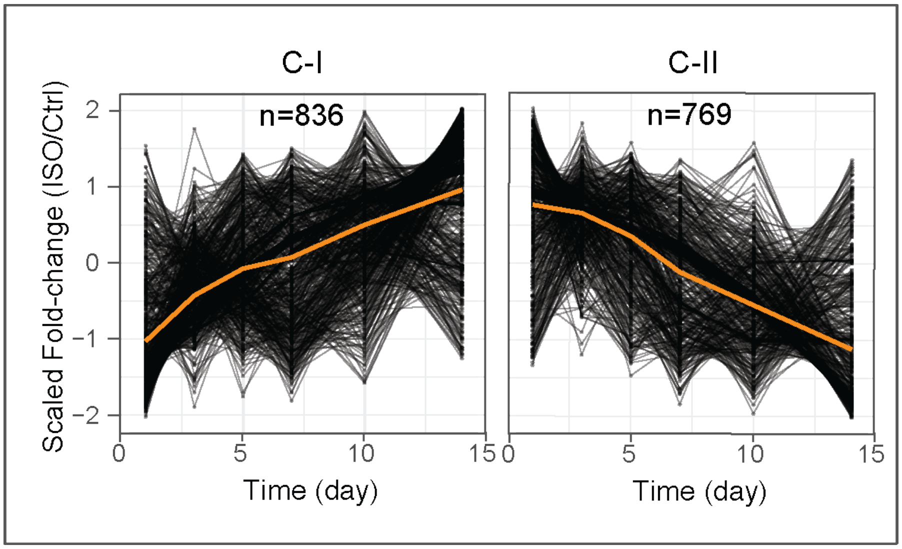

Supplemental Figure S3. K-means clustering of O-PTM data with K=2. Out of 1605 preprocessed O-PTMs, 836 and 769 O-PTMs were clustered into clusters I and II, respectively. Exploring the O-PTM data demonstrated that setting a small K (e.g., as suggested by the silhouette plot) may ignore granular temporal trends.

(One-column Figure, Color Figure)

SUPPLEMENTAL BIBLIOGRAPHY

[1] Aghabozorgi S, Seyed Shirkhorshidi A, Ying Wah T. Time-series clustering – A decade review. Inf Syst 2015; 53:16–38. https://doi.org/10.1016/j.is.2015.04.007.

[2] Liao TW. Clustering of time series data — a survey. Pattern Recognition 2005; 38: 1857–74. https://doi.org/10.1016/j.patcog.2005.01.025.

[3] Montero P, Vilar JA. TSClust : an *R* package for time series clustering. J Stat Softw 2014; 62(1): 1–43. https://doi.org/10.18637/jss.v062.i01.

[4] Berndt DJ, Clifford J. Using dynamic time warping to find patterns in time Series. AAAIWS'94: Proceedings of the 3rd International Conference on Knowledge Discovery and Data Mining 1994; 359–370.

[5] Chong J, Wishart DS, Xia J. Using metaboanalyst 4.0 for comprehensive and integrative metabolomics data analysis. Curr Protoc Bioinformatics 2019; 68(1): e86. https://doi.org/10.1002/cpbi.86.

[6] Rohart F, Gautier B, Singh A, Lê Cao K-A. mixOmics: An R package for 'omics feature selection and multiple data integration. PLoS Comput Biol 2017; 13(11): e1005752. https://doi.org/10.1371/journal.pcbi.1005752.

[7] Wold H. Path models with latent variables: The NIPALS approach. In: Blalock HM, Aganbegian A, Borodkin FM, Boudon R, Capecchi V (Eds.), Quantitative Sociology, New York: Academic Press; 1975, pp. 307–57.

[8] Troyanskaya O, Cantor M, Sherlock G, Brown P, Hastie T, Tibshirani R, et al. Missing value estimation methods for DNA microarrays. Bioinformatics 2001; 17(6): 520–5. https://doi.org/10.1093/bioinformatics/17.6.520.

[9] Rousseeuw PJ. Silhouettes: A graphical aid to the interpretation and validation of cluster analysis. J Comput Appl Math 1987; 20: 53–65. https://doi.org/10.1016/0377-0427(87)90125-7.

SUPPLEMENTAL TABLES.

Supplemental Table S1. A list of 1,605 cysteine O-PTMs and their abundance ratios (i.e., abundance of ISO-treated over control mice) across 6 timepoints.

UniProt ID	Modified Amino Acid	Cysteine PTM type	Day 1	Day 3	Day 5	Day 7	Day 10	Day 14
A2ASQ1	C123	reversible	0.81	1.02	0.94	0.93	NA	NA
A2ASQ1	C1921	reversible	0.9	1.23	0.77	1.09	NA	1.11
A2ASQ1	C441	reversible	0.84	0.79	0.95	0.89	0.79	0.87
A2AUC9	C35	reversible	NA	1.63	1	0.98	0.63	0.96
A2AUC9	C593	reversible	1.4	NA	1.34	1.11	0.68	0.83
O08528	C368	reversible	1.21	NA	0.64	0.7	0.83	0.61
O08528	C375	reversible	1.26	1.97	0.72	0.69	NA	0.68
O08528	C909	reversible	1.29	1.17	0.74	0.73	1.12	0.78
O08529	C82	reversible	1.39	0.92	0.55	0.95	NA	1.08
O08553	C248	reversible	0.89	1.51	1.15	1.36	1.52	1.46
O08553	C504	reversible	1.03	1.11	1.17	1.1	1.47	0.96
O08677	C339	reversible	0.98	0.61	1.42	0.98	0.87	1.26
O08709	C47	reversible	0.87	1.12	1.07	0.81	1.25	1.36
O08749	C477	reversible	NA	0.89	1.05	NA	0.72	0.75
O08749	C80	reversible	1.02	1.09	0.8	0.89	0.75	1.33
O08749	C85	reversible	1.01	1.1	0.71	0.86	0.92	1.18
O08796	C145	reversible	0.93	0.95	0.73	0.82	0.82	NA
O08807	C54	reversible	1.12	1.06	1.58	1.54	1.39	NA
O09161	C53	reversible	1.07	1.06	0.95	0.72	0.78	1.05
O35459	C186	reversible	1.09	0.93	0.99	0.66	0.86	1.29
O35459	C91	reversible	1.05	0.92	0.84	0.53	0.62	0.88
O35604	C177	reversible	0.78	0.9	1.11	0.9	0.97	1.38
O35678	C55	reversible	NA	1.05	1.12	1.02	NA	1.21
O35682	C273	reversible	0.88	1.12	1	1.02	0.78	1.12
O35855	C136	reversible	0.8	0.95	1.26	NA	NA	1
O35855	C148	reversible	NA	0.69	0.87	0.7	0.89	0.83
O35855	C229	reversible	0.99	0.97	1.21	0.86	0.78	1.13
O35855	C343	reversible	1.08	0.98	0.88	0.8	0.67	0.97
O35855	C346	reversible	1.33	1.13	1.44	0.9	NA	NA
O35855	C93	reversible	1.12	1.34	1.01	NA	1.04	1.04
O55023	C183	reversible	NA	1.22	1.28	1.23	1.61	1.55
O55040	C5	so3h	1.07	1.32	NA	1.29	NA	1.45

O55040	C8	so3h	1.07	1.32	NA	1.29	NA	1.45
O55126	C80	reversible	1.41	1.02	1.14	1.22	NA	NA
O55126	C88	reversible	1.06	1.09	1.14	0.82	1.09	1.21
O55137	C412	reversible	1.89	0.96	1.08	NA	NA	0.64
O55143	C349	reversible	0.76	0.92	0.78	0.89	0.92	1.06
O55143	C377	reversible	0.92	1.48	0.78	0.77	1.3	1
O55143	C447	reversible	0.88	1	0.82	0.76	1.1	1.1
O55143	C471	reversible	0.97	1.37	0.67	0.85	1.69	1.61
O55143	C635	reversible	1.04	1.24	0.79	0.81	1.17	1.1
O55143	C669	reversible	1	1.32	0.88	0.79	1.17	1.12
O55143	C875	reversible	0.96	1.11	0.67	0.74	1.17	0.96
O70250	C153	reversible	1.05	1.29	1.08	0.71	1	1.46
O70325	C102	reversible	1.05	1.26	1.52	1.23	1.14	1.24
O70423	C430	reversible	0.98	0.66	1.04	0.91	0.98	0.97
O70433	C165	reversible	1.09	NA	0.72	1.25	1.31	1.66
O70433	C272	reversible	1.33	NA	0.71	NA	2.27	1.08
O70433	C275	reversible	0.78	1.31	0.91	1.24	NA	1.59
O70468	C715	reversible	1.1	1.24	0.67	0.67	1.14	1.01
O88342	C325	reversible	1.14	1.69	1.1	0.8	1.12	1.85
O88569	C50	reversible	1.46	2.61	1.49	NA	NA	1.23
P01027	C1101	reversible	1.14	1.38	1.86	1.63	1.23	1.14
P01027	C1389	reversible	1.04	1.17	1.5	1.77	1.65	1.37
P01027	C1458	reversible	1.05	1.18	1.7	1.44	1.11	1.06
P01027	C1489	reversible	1.24	1.37	1.76	1.49	1.47	1.35
P01027	C1511	reversible	1.24	1.3	1.94	NA	1.33	2.19
P01027	C1537	reversible	1.08	1.36	1.71	1.77	1.33	1.13
P01027	C559	reversible	1.22	1.47	2.04	1.64	1.36	1.27
P01027	C626	reversible	1.18	1.49	1.93	1.46	1.44	NA
P01027	C720	reversible	1.04	1.29	1.92	1.6	1.3	1.22
P01027	C727	reversible	1.11	1.1	2.06	1.18	0.68	0.87
P01027	C728	reversible	1.07	1.03	1.56	1.38	1.01	1.25
P01027	C816	reversible	1.26	1.51	1.86	1.75	1.65	1.29
P01027	C873	reversible	0.95	1.5	1.86	1.41	1.55	1
P01029	C1612	reversible	1.01	1.16	1.37	0.82	1.4	0.85
P01837	C26	reversible	0.87	1.16	1.67	NA	1.59	1.34
P01868	C138	reversible	1	1.16	1.27	0.52	1.43	1.82
P01872	C135	reversible	1.24	1.09	1.29	NA	0.36	1.4
P01872	C27	reversible	1.09	0.96	1.07	1.07	NA	1.39
P01872	C88	reversible	1.2	NA	0.96	1.18	0.83	1.1

P01942	C105	so3h	0.73	1.22	NA	1.14	1.67	0.91
P02088	C94	reversible	0.84	1.52	0.99	1.13	1.36	1.64
P02089	C94	reversible	1.02	NA	1.2	1.01	1.31	1.45
P02463	C1616	reversible	0.86	1.43	1.38	1.4	1.53	1.19
P02468	C349	reversible	0.92	0.75	1	1.01	0.84	1.17
P02468	C472	reversible	0.9	1.03	NA	NA	1.12	1.26
P02469	C170	reversible	NA	0.92	1.38	0.96	NA	0.97
P02469	C1785	reversible	0.86	1.04	1.53	1.19	1.02	0.91
P02469	C190	reversible	0.9	0.79	1.09	0.96	NA	0.83
P02469	C455	reversible	1.06	1.3	1.08	NA	1.89	0.84
P04186	C188	reversible	1.06	1.29	1.04	0.92	1.24	0.85
P04186	C59	reversible	1.15	1.29	1.06	NA	1.38	NA
P04186	C593	reversible	1.03	0.76	1.18	1.04	2.53	1.03
P04186	C596	reversible	1.17	1.52	NA	1.17	NA	1.01
P05064	C178	reversible	1.14	1.08	1.1	1.05	1	1.34
P05064	C202	reversible	0.95	0.82	0.96	1.37	1.15	1.26
P05064	C339	reversible	1.07	1.25	1.03	1.36	1.27	1.8
P05064	C73	reversible	0.97	1.13	0.97	1.21	1.29	1.21
P05201	C391	reversible	0.96	1.48	1.31	1.08	1.47	1.33
P05202	C106	reversible	0.96	1.08	0.92	0.89	1.21	1.14
P05202	C187	reversible	1	1.16	1.05	0.87	1.25	1.29
P05202	C212	reversible	1.07	0.81	1.06	0.96	NA	0.91
P05202	C295	reversible	0.95	1.49	1.06	0.86	1.73	1.19
P05202	C382	reversible	1.05	0.83	0.92	1.05	NA	NA
P06151	C163	reversible	1.1	1.27	1.24	1.12	1.5	1.64
P06684	C715	reversible	0.91	1.18	0.9	1.13	1.07	0.63
P06745	C404	reversible	0.98	1.07	1.08	0.86	1.55	1.28
P06909	C1048	reversible	1.28	1.36	1.82	1.27	1.7	NA
P06909	C1206	reversible	1.77	1.04	1.54	1.18	1.09	1.37
P06909	C178	reversible	0.55	0.74	1.27	NA	NA	1.09
P06909	C267	reversible	0.93	1.5	1.38	0.94	1.38	0.69
P06909	C294	reversible	1.52	1.18	1.94	1.08	1.24	1.31
P06909	C536	reversible	1.2	1.14	1.26	1.05	1.15	1.3
P06909	C569	reversible	1.32	1.47	1.63	1.17	NA	NA
P06909	C85	reversible	1.61	1.84	2.01	1.46	1.79	1.18
P06909	C867	reversible	1.46	1.37	2.16	1.69	1.32	1.14
P06909	C994	reversible	1.32	NA	1.45	1.28	1.44	1.15
P07310	C146	reversible	0.9	1.46	1.1	0.86	1.42	1.44
P07310	C254	reversible	1.24	1.15	0.83	0.9	1.43	1.49

P07310	C283	reversible	NA	1.37	1.02	1.12	1.48	1.41
P07724	C114	reversible	1.08	1.26	1.26	0.94	1.25	1.43
P07724	C115	reversible	1.13	1.32	1.42	0.95	1.31	1.72
P07724	C148	reversible	1.12	1.37	1.56	0.87	1.35	1.49
P07724	C201	reversible	1.17	1.33	1.27	0.9	1.02	1.44
P07724	C289	reversible	1.1	1.6	1.6	0.8	1.56	1.49
P07724	C302	reversible	1.26	1.6	1.74	1.32	1.43	1.21
P07724	C303	reversible	1.47	1.27	1.45	0.92	0.88	1.56
P07724	C313	reversible	1.26	1.14	1.23	NA	1.09	1.34
P07724	C340	reversible	1.08	1.08	1.17	0.94	1.14	1.34
P07724	C393	reversible	0.84	0.77	1.3	1.46	1.11	1.14
P07724	C416	reversible	1.02	1.86	1.24	0.9	1.97	1.23
P07724	C461	reversible	1.02	1.22	1.13	1.31	1.21	1.2
P07724	C462	reversible	0.85	1.02	1.28	1.3	1.14	1.39
P07724	C472	reversible	1	1.4	1.35	1.15	1.4	1.37
P07724	C472	so3h	1.03	1.45	0.77	1.74	1.4	1.22
P07724	C485	reversible	1.07	1.68	1.39	1.18	1.36	1.22
P07724	C500	reversible	0.94	1.2	1.2	1.32	1.08	1.18
P07724	C501	reversible	0.98	0.96	1.22	1.16	1.21	1.1
P07724	C511	reversible	1.03	1.4	1.56	1.28	1.37	1.31
P07724	C538	reversible	1.12	1.3	1.23	1.25	1.34	1.63
P07724	C58	reversible	1.25	1.1	1.28	1.23	1.34	1.16
P07724	C582	reversible	1.1	1.28	1.4	1.08	1.34	1.43
P07724	C583	reversible	1.03	1.12	1.3	1.02	1.17	1.52
P07724	C591	reversible	1.06	1.43	1.41	1.21	1.33	1.58
P07724	C77	reversible	1.15	1.23	1.33	0.97	1.24	1.48
P07724	C86	reversible	1.11	1.43	1.34	1.13	1.33	1.38
P07724	C99	reversible	1.01	1.76	1.67	1.22	1.62	1.38
P08228	C147	reversible	0.88	1.39	1.12	0.7	1.7	1.01
P08249	C212	reversible	1.27	1.55	1.49	1.13	1.23	1.67
P08249	C275	reversible	1.03	1.48	1.14	0.59	1.39	1.37
P08249	C285	reversible	1.04	1.42	1.09	0.74	1.39	1.36
P08249	C89	reversible	0.98	1.3	1.13	0.78	1.19	1.29
P08249	C93	reversible	1.03	1.46	1.23	0.77	1.38	1.45
P08752	C140	reversible	1.01	1.12	1.4	1.06	NA	NA
P08752	C255	reversible	0.93	1.41	1.25	0.82	1.05	1.05
P08752	C287	reversible	1.76	1.37	1.14	0.88	NA	1
P08752	C66	reversible	1.03	0.92	0.7	0.51	NA	0.89
P09055	C691	reversible	1.04	NA	1.28	0.55	NA	1.24

P09411	C108	reversible	1.34	0.94	1.39	NA	NA	1.33
P09411	C99	reversible	NA	1.53	0.92	0.96	NA	0.98
P09541	C74	reversible	0.89	NA	1.51	0.78	NA	1.57
P10126	C234	reversible	0.89	2.01	1.7	1.46	1.41	0.95
P10493	C1230	so3h	0.82	0.89	1.02	0.93	1.06	NA
P10493	C1232	so3h	0.82	0.89	1.02	0.93	1.06	NA
P10493	C423	reversible	0.93	1.28	1.28	0.91	1.59	1
P10493	C616	reversible	0.98	0.8	0.98	0.84	0.85	0.97
P10493	C748	reversible	1	1.01	1.32	0.83	0.89	1.07
P10493	C985	reversible	0.91	0.74	1.1	NA	NA	0.98
P11152	C243	reversible	1.05	0.87	0.92	0.61	NA	1.3
P11276	C374	reversible	1.22	NA	1.9	1.72	1.14	NA
P11276	C420	reversible	1.22	2.13	1.67	1.57	1.56	NA
P11499	C521	reversible	0.95	1.84	0.69	1.11	NA	1.04
P12382	C351	reversible	1.01	1.5	1.11	0.88	1.31	1.32
P12382	C708	reversible	0.99	1.03	1.19	1.37	1.11	1.21
P13020	C329	reversible	0.96	1.42	1.25	0.85	NA	1.28
P13020	C670	reversible	1.08	1.21	1.59	1.28	NA	1.14
P14094	C126	reversible	1.08	0.94	0.88	0.61	1.1	0.89
P14094	C159	reversible	1.15	0.69	0.92	0.95	0.86	NA
P14094	C175	reversible	1.03	1.24	1.23	0.81	0.89	0.78
P14094	C214	reversible	0.85	0.98	1.05	0.89	0.75	0.9
P14152	C137	reversible	1.15	1.73	1.3	0.78	1.77	1.66
P14152	C154	reversible	1.19	1.38	1.25	0.86	1.22	1.65
P14211	C137	reversible	1	1	1.4	0.69	0.92	0.93
P14824	C552	reversible	1.09	1.25	1.81	0.97	0.81	1.58
P14824	C669	reversible	0.99	1.14	1.63	0.86	1	1.74
P16045	C61	reversible	1.1	1.24	1.12	0.72	1.14	1.12
P16125	C294	reversible	1.19	0.67	0.89	NA	0.91	0.76
P16858	C150	reversible	0.99	1.48	1.03	1.22	1.24	1.53
P16858	C154	reversible	1.29	1.34	0.96	1.23	1.11	1.46
P16858	C22	reversible	1.02	1	0.85	0.91	1.12	1.25
P16858	C245	reversible	1.03	1.38	1.1	1.44	1.52	1.45
P17182	C337	reversible	0.85	1.26	2	1.59	NA	1.26
P17182	C339	reversible	1.13	1.24	1.2	1.06	NA	0.83
P17563	C268	reversible	1.07	1.17	1.25	1.04	1.16	1.43
P17563	C31	reversible	1.08	0.97	0.96	0.93	1.04	1.4
P17563	C371	reversible	1.05	0.87	0.86	0.74	0.93	1.21
P17710	C684	reversible	1.14	0.98	0.77	0.87	0.96	1.03

P17710	C869	reversible	1.05	1.17	0.69	0.96	NA	0.43
P17742	C161	reversible	1.22	1.16	1.47	0.78	1.1	1.14
P17742	C62	reversible	1.23	1.23	1.74	0.85	1.19	1
P17751	C117	reversible	0.94	NA	1.58	0.74	NA	1.38
P17751	C268	reversible	1.17	1.73	1.33	1.07	1.16	1.89
P18242	C117	reversible	0.99	0.72	1.06	1.32	NA	1.07
P18242	C288	reversible	1	0.82	0.96	1	0.73	1.12
P18242	C327	reversible	0.8	0.92	1.02	0.74	0.94	0.9
P19221	C104	reversible	0.86	NA	1.35	1.16	1.02	NA
P19221	C264	reversible	0.96	1.09	1.17	1.27	0.85	1.01
P19221	C333	reversible	0.97	0.98	1.44	1.14	1.09	1.01
P19221	C388	reversible	0.77	1.01	0.99	1.14	1.1	1.03
P20108	C230	reversible	0.92	0.98	0.91	0.88	0.83	1.26
P20918	C103	reversible	1.18	1.4	0.97	1.27	1.11	1.98
P20918	C49	reversible	1.19	0.9	1.42	1.28	1.02	1.27
P20918	C586	reversible	1.11	1.24	1.31	1.1	1.03	NA
P21550	C337	reversible	1.05	1.42	1.5	1.09	1.94	1.56
P21550	C339	reversible	1.32	1.4	1.72	1.06	NA	1.85
P21614	C220	reversible	1.27	1.13	1.46	0.96	1.1	1.47
P21956	C290	reversible	1.09	1.21	1.1	1.05	NA	1.18
P21956	C303	reversible	1.32	1.26	0.95	0.85	NA	NA
P21981	C619	reversible	1.03	1.72	1	1.28	0.94	0.9
P23242	C198	reversible	1.02	0.83	0.93	0.82	NA	0.86
P23953	C116	reversible	0.68	1.3	1.72	NA	1.12	NA
P24270	C232	reversible	1.26	0.8	1	0.89	0.99	0.81
P24270	C377	reversible	1.21	1.23	1.34	0.99	1.48	1.03
P24270	C425	reversible	1.4	1.26	1.03	1.25	1	1
P25444	C229	reversible	1.1	1.3	1.76	1.25	1.33	1.33
P26040	C117	reversible	0.96	2.04	NA	1.05	NA	0.89
P26262	C419	reversible	0.99	1.18	1.25	0.9	0.91	0.8
P26443	C112	reversible	0.93	1.23	1.39	1.3	1.36	1.14
P26638	C300	reversible	1.08	1.5	1.31	1.32	1.25	1.07
P27773	C85	reversible	0.84	1.08	1.25	1.36	NA	NA
P28474	C240	reversible	0.96	0.95	0.84	1.01	1.08	0.83
P28665	C1129	reversible	0.87	1.13	1.21	0.95	1.1	NA
P28665	C276	reversible	0.92	1.63	1.01	0.97	1.42	1.47
P28665	C587	reversible	0.89	1.95	1.02	1.03	1.67	1.35
P28665	C680	reversible	0.96	1.75	1.19	1.22	1.77	1.83
P28665	C86	reversible	1.05	1.52	0.84	0.92	1.05	1.41

P28665	C923	reversible	1.03	1.14	0.85	1.03	0.85	1.24
P29758	C150	reversible	1.05	1.15	1.01	0.82	0.9	0.89
P32261	C54	reversible	0.81	0.98	0.96	0.8	NA	NA
P34914	C120	reversible	0.86	1.5	1.33	1	NA	NA
P34914	C230	reversible	1.09	0.89	1.14	0.83	NA	1.12
P35486	C181	reversible	1.06	1.12	1.15	1.04	1.09	1.41
P35486	C218	reversible	0.93	2.04	0.97	1.07	2.03	1.08
P35486	C222	reversible	1.18	1.2	1.2	1.02	1.67	1.17
P35486	C261	reversible	1.06	1.13	0.8	0.99	1.03	1.04
P35486	C273	reversible	1.16	0.99	1.22	1.07	0.8	1.35
P35762	C156	reversible	NA	1.53	0.93	1.32	0.76	1.02
P35762	C157	reversible	1.59	1.32	1.07	NA	0.6	NA
P35762	C175	reversible	0.81	1.18	1.15	1.05	NA	1.2
P35979	C141	reversible	1.29	NA	1.55	0.98	NA	0.89
P38647	C608	reversible	0.9	0.89	0.82	1.11	1.15	1.06
P40124	C92	reversible	0.93	0.96	1.8	1.65	NA	1.18
P41216	C133	reversible	1.08	0.92	0.85	0.87	0.68	0.96
P41216	C242	reversible	0.71	NA	0.98	1.4	NA	0.78
P41216	C298	reversible	0.94	0.76	0.94	0.82	0.84	0.89
P41216	C510	reversible	0.98	0.7	0.86	0.74	0.77	0.92
P41216	C55	reversible	0.85	0.83	0.99	0.96	1.03	0.99
P41216	C626	reversible	1	0.91	0.9	0.98	0.81	0.82
P42208	C111	reversible	NA	1.17	1.34	0.82	1.31	1.31
P45376	C187	reversible	1.14	1.18	0.8	0.62	0.6	1.24
P45376	C200	reversible	1.1	0.79	0.84	0.86	0.7	1.15
P45376	C304	reversible	1.48	1	0.87	1.05	0.62	0.81
P45377	C200	reversible	NA	0.97	1.03	1.35	NA	1.03
P45591	C80	reversible	0.99	1.41	1.34	1.31	1.23	1.24
P46412	C156	reversible	0.93	1.42	1.34	1.2	1.57	1.27
P46935	C365	reversible	1.17	1.53	1.85	NA	NA	1.13
P47738	C388	reversible	1.17	0.99	1.07	0.78	0.8	1.17
P47738	C68	reversible	1.11	0.8	0.96	0.77	0.63	0.81
P47857	C114	reversible	1	1.28	1.02	0.97	1.1	1.14
P47857	C709	reversible	1.07	1.18	1.15	0.93	1.2	1.21
P47934	C449	reversible	1.07	1.16	0.88	0.9	1.07	0.83
P48774	C38	reversible	1.31	1.1	1.02	NA	0.61	NA
P48962	C129	reversible	0.92	0.9	0.98	NA	NA	0.79
P48962	C57	reversible	1.05	1.74	1.08	1.75	1.52	1.43
P50136	C194	reversible	1.16	NA	1.14	0.92	0.86	0.83

P50247	C195	reversible	1.21	NA	1.61	NA	1.9	1.92
P50247	C228	reversible	0.91	NA	1.27	NA	1.36	1.14
P50396	C302	reversible	1.13	1.27	1.4	1.58	0.86	1.58
P50396	C317	reversible	NA	1.45	NA	1.19	1.19	1.04
P50462	C79	reversible	1.46	NA	1.28	0.96	NA	0.84
P50544	C216	reversible	1.02	0.8	1.18	0.95	NA	0.62
P50544	C238	reversible	1.09	1.09	1.16	0.98	1.04	0.82
P50544	C478	reversible	1.05	0.95	1.13	0.93	1.09	1.21
P50544	C604	reversible	0.85	0.89	1.76	1.35	NA	NA
P51174	C342	reversible	0.99	1	1.09	0.89	0.56	1.05
P51174	C351	reversible	0.93	1.01	1.01	0.75	1.22	1.04
P52196	C248	reversible	0.91	0.7	1.33	1.02	1.11	1.23
P52480	C326	reversible	0.97	NA	0.91	1.46	1.11	1.28
P52480	C358	reversible	1.02	0.97	0.91	0.79	0.97	1.14
P52480	C474	reversible	1.07	1.25	1.06	1	1.15	1.11
P52480	C49	reversible	1.14	1.19	0.95	1.02	1.1	1.38
P52503	C107	reversible	1.24	1.12	1.55	1.62	NA	NA
P52503	C79	reversible	0.98	1.08	1.16	0.65	1.15	1.22
P52825	C489	reversible	0.93	1.04	1.06	0.82	0.94	0.96
P52825	C512	reversible	1.02	0.8	1.51	NA	0.77	NA
P52825	C84	reversible	1.4	0.74	1.23	1.98	0.7	0.8
P53395	C279	reversible	1.1	1.02	0.7	0.87	1.66	1.02
P53395	C333	reversible	1	1.27	1.07	0.92	0.75	0.8
P54071	C113	reversible	0.9	1.18	0.91	0.75	0.87	0.74
P54071	C154	reversible	0.94	2.34	2.44	9.2	0.95	NA
P54071	C308	reversible	0.94	1.09	0.93	0.66	1.02	0.95
P54071	C402	reversible	1.05	1	0.91	0.6	0.98	1.04
P54071	C418	reversible	1.04	1.08	0.96	0.66	0.82	0.98
P55066	C1160	so3h	0.81	0.96	1.44	1.01	1.07	1.27
P55264	C159	reversible	1.23	0.9	1.81	0.86	NA	NA
P56382	C19	reversible	1.18	1.2	1.19	0.74	1.14	1.27
P56391	C30	reversible	1.01	1.06	1.26	0.9	NA	NA
P56391	C54	reversible	0.99	1.63	1.25	0.91	NA	NA
P58021	C181	reversible	0.89	0.99	1.05	1.29	NA	NA
P58021	C193	reversible	0.94	1.42	1.09	1	0.79	0.99
P58252	C290	reversible	1.26	1.64	1.1	1.46	1.3	0.8
P58252	C41	reversible	1.08	1.64	0.78	1.31	NA	NA
P58252	C466	reversible	NA	1.31	0.9	NA	1.04	1.53
P58252	C567	reversible	1.22	1.26	0.9	0.95	1.04	1.02

P58252	C591	reversible	0.81	1.47	NA	1.21	NA	0.34
P58252	C693	reversible	1.08	1.08	1.09	NA	0.89	0.82
P58252	C728	reversible	0.68	1.03	2.56	NA	0.74	1.27
P58281	C786	reversible	0.91	0.94	0.66	0.86	0.82	0.91
P58281	C856	reversible	NA	NA	0.76	0.8	1.09	0.54
P62242	C174	reversible	1.22	2.04	1.43	1.2	1.19	1.59
P62281	C116	reversible	1.15	NA	1.63	1.29	NA	1.08
P62631	C326	reversible	1.11	0.99	0.94	0.92	0.64	NA
P62702	C181	reversible	1.04	1.01	1.63	1.42	NA	NA
P62702	C41	reversible	0.94	2	NA	1.49	1.94	0.97
P62717	C109	reversible	1.02	NA	1.49	1.19	1.24	1.26
P62737	C219	reversible	0.95	1.08	1.02	1.16	1.47	NA
P62737	C259	reversible	0.88	1.19	1.29	1.11	1.28	NA
P62754	C12	reversible	1.25	2.15	1.5	1.32	1.44	1.05
P62827	C112	reversible	1.42	1.03	0.55	1.34	NA	0.82
P62908	C119	reversible	0.98	1.29	1.66	1.18	0.94	1.01
P62908	C134	reversible	0.81	NA	1.21	1.24	1.01	NA
P62908	C97	reversible	1.49	NA	1.46	1.38	1.11	NA
P63001	C157	reversible	1.11	1.24	1.42	1.18	1.28	1.27
P63001	C178	reversible	1.03	1.51	1.06	1.14	1.13	1.29
P63017	C17	reversible	0.92	1	1.07	1.16	NA	1.5
P63017	C603	reversible	1.1	1.16	1.11	0.94	1.39	1.29
P63087	C62	reversible	NA	1.22	0.98	1	NA	0.94
P68033	C219	reversible	NA	1	1.4	1.09	0.97	1.21
P68033	C259	reversible	1.01	0.92	1.15	NA	1.18	1.33
P68040	C182	reversible	1.11	NA	1.47	1.29	1.27	NA
P68040	C249	reversible	1.05	1.7	1.93	1.38	1.13	1.32
P70404	C148	reversible	1.01	0.95	0.89	1	1.19	1.27
P70404	C235	reversible	0.94	1.21	1.03	0.83	1.49	1.27
P70404	C236	reversible	0.95	1.15	0.79	1.06	1.07	1.09
P70404	C333	reversible	1.11	1.2	1.27	1.34	1.33	1.11
P70670	C354	reversible	1.13	1.44	1.36	0.96	1.22	0.8
P97351	C201	reversible	1.19	2.09	1.49	1.03	3.04	1.55
P97351	C96	reversible	1.04	1.47	1.37	1.38	1.14	0.98
P97370	C170	reversible	0.86	0.97	1.03	0.82	NA	NA
P97370	C191	reversible	0.81	0.88	1.13	1.18	1.04	1.16
P97370	C249	reversible	1.04	0.89	0.94	0.72	0.87	0.79
P97443	C125	reversible	1.04	0.99	0.73	0.78	0.84	0.96
P97447	C273	reversible	NA	1.24	2.12	2.24	NA	1.79

P97467	C226	reversible	0.79	0.85	1.63	NA	1.64	1.32
P97467	C292	reversible	0.99	0.9	1.1	6.96	1.12	0.77
P97467	C46	reversible	0.75	1.1	1.67	1.92	1.23	0.9
P97927	C1222	reversible	NA	NA	1.54	0.98	1.06	1.39
Q01768	C109	reversible	1.1	1.09	1.28	0.84	1.17	1.27
Q01853	C105	reversible	1.25	1.21	1.25	1.16	1.15	1.29
Q01853	C209	reversible	1.18	1.06	1.21	NA	1.06	0.58
Q01853	C522	reversible	1.16	1.54	1.45	1.31	1.43	1.46
Q01853	C572	reversible	1.06	1.24	1.26	1.31	1.11	1.09
Q01853	C691	reversible	1.59	1.43	1.34	1.73	NA	0.93
Q02053	C481	reversible	1.17	1.13	1.44	1.2	1.35	NA
Q02566	C1750	reversible	0.91	1.55	0.83	0.71	1.06	0.72
Q02566	C37	reversible	1	1.86	0.54	0.73	0.73	0.89
Q02566	C949	reversible	0.88	NA	0.92	NA	0.83	0.77
Q03265	C244	reversible	0.92	1.27	1.52	1.26	1.35	1.14
Q04447	C146	reversible	1.16	1.53	NA	NA	2.41	1.61
Q04447	C254	reversible	0.89	1.68	2.01	1.27	1.9	1.29
Q05793	C1156	reversible	0.72	1.09	1.01	0.99	0.98	0.7
Q05793	C2313	reversible	0.98	NA	1.1	0.63	0.98	1.29
Q05793	C2456	reversible	0.7	NA	1	0.88	1.09	1
Q05793	C3137	reversible	0.89	1.05	1.21	1.07	1.12	0.95
Q05793	C731	reversible	0.93	1.25	1.06	0.9	1.04	0.86
Q05793	C811	reversible	0.68	1.34	NA	1.03	1.24	1.04
Q05793	C892	reversible	0.86	1.26	1.22	0.93	1.18	0.9
Q05BC3	C769	reversible	1.37	1.1	1.37	1.24	NA	NA
Q07113	C513	reversible	0.84	1.37	1.46	1.16	1.8	1.26
Q07113	C724	reversible	0.92	1.3	1.27	1.25	1.01	0.84
Q07113	C807	reversible	1.06	1.06	1.24	1.29	1.2	NA
Q07113	C886	reversible	1	1.3	1.38	1.22	0.99	1.07
Q07417	C151	reversible	0.98	1.08	1.26	1.02	1.47	1.29
Q07417	C289	reversible	1.85	1.18	1.08	NA	0.99	1.12
Q07456	C336	reversible	1.02	1.23	1.89	1.51	0.94	NA
Q08481	C470	reversible	1.48	1.21	1.26	1.07	1.09	1.09
Q08857	C272	reversible	0.99	0.99	0.81	0.74	0.87	1.12
Q08857	C311	reversible	1.01	1.28	1.42	0.91	NA	1.11
Q1XH17	C105	reversible	1.19	1.25	0.97	0.72	1.17	1.08
Q1XH17	C108	reversible	1.06	NA	0.71	0.55	NA	0.92
Q1XH17	C242	reversible	1.18	1.15	0.84	0.97	1.18	1.24
Q2TPA8	C11	reversible	1.07	0.74	1.31	0.78	0.53	NA

Q2TPA8	C218	reversible	0.94	0.77	0.95	0.92	0.67	0.9
Q3TC72	C110	reversible	0.98	0.81	1.11	1.03	NA	0.9
Q3TC72	C119	reversible	0.89	0.77	NA	0.98	0.92	NA
Q3TC72	C214	reversible	1.07	0.66	0.94	1.22	1.21	NA
Q3U269	C354	reversible	0.75	0.93	NA	NA	0.79	0.85
Q3ULD5	C167	reversible	1	1.05	1.15	1	0.99	1.22
Q3ULD5	C267	reversible	1.13	0.79	0.98	0.93	0.82	NA
Q3ULD5	C391	reversible	1.28	1.12	1.14	0.74	0.89	1.36
Q3ULD5	C392	reversible	1.13	1.23	1.86	1.23	1.09	1
Q3ULD5	C431	reversible	1.39	0.75	1.15	1.16	1.23	1.27
Q3V1T4	C571	reversible	0.94	NA	1.43	1.67	NA	0.96
Q3V3R4	C130	reversible	0.91	0.99	NA	0.97	NA	0.55
Q4FZG7	C66	so3h	0.81	1.81	0.79	0.81	NA	NA
Q60597	C395	reversible	1.04	0.96	0.95	0.75	0.94	0.94
Q60597	C487	reversible	1.05	1	1.04	1.17	0.75	0.97
Q60597	C566	reversible	1.06	1.18	1.21	0.89	1.2	1.14
Q60597	C904	reversible	1.1	1.21	1.1	0.87	1.22	1.27
Q60675	C1141	reversible	0.91	1.03	1.26	0.87	0.89	0.62
Q60675	C1447	reversible	0.75	0.73	0.72	0.6	NA	0.67
Q60675	C1502	reversible	1.03	0.99	1.7	0.74	1.05	0.81
Q60675	C1544	reversible	0.22	1.1	1.11	0.95	1.07	NA
Q60675	C2905	reversible	0.95	0.37	0.95	0.74	NA	0.71
Q60675	C349	reversible	0.98	0.85	1.08	0.96	0.81	NA
Q60675	C493	reversible	0.9	0.85	1.14	0.78	0.97	0.88
Q60692	C76	reversible	1.02	1.41	1.49	1.23	1.84	NA
Q60759	C176	reversible	1.55	0.81	1.2	1.01	1.13	NA
Q60854	C102	reversible	1.26	1.08	1.28	0.98	NA	1.4
Q60854	C352	reversible	0.97	1.2	1.09	1.45	NA	1.13
Q60864	C461	reversible	1.03	0.67	NA	NA	0.9	1.27
Q60930	C48	reversible	1.06	1.09	1.08	1.1	0.75	1.53
Q60930	C77	reversible	1.1	1.28	1.22	1	NA	1.12
Q60931	C65	reversible	1.06	1.01	1.07	0.97	0.99	1.38
Q60932	C140	reversible	1.01	0.94	0.85	0.78	0.95	1.36
Q60932	C245	reversible	1.19	0.92	0.97	0.91	0.97	1.43
Q60936	C265	reversible	0.73	1.05	1.32	0.64	0.81	0.76
Q60936	C403	reversible	0.93	0.9	0.93	0.86	0.62	1.06
Q61129	C96	reversible	0.83	1.19	1.33	0.91	0.68	NA
Q61147	C199	reversible	1.15	1.36	1.51	1.23	1.61	1.16
Q61147	C713	reversible	0.94	1.4	1.55	1.65	1.47	1.33

Q61147	C895	reversible	1.05	1.17	1.25	1.01	1.11	1.14
Q61292	C185	reversible	0.89	1.12	1.07	0.82	1.29	1
Q61292	C505	reversible	0.77	0.89	0.95	NA	NA	0.88
Q61292	C906	reversible	0.86	0.88	0.9	0.72	1.07	NA
Q61316	C140	reversible	1.13	1.27	1.22	1.15	NA	1.18
Q61316	C380	reversible	0.96	1.32	1.37	1.19	1.21	NA
Q61316	C780	reversible	1.26	1.24	1.39	1.24	1.07	1.07
Q61425	C201	reversible	1	0.92	0.95	0.81	0.92	1.03
Q61425	C99	reversible	1.05	1.02	1.41	NA	0.6	NA
Q61543	C320	reversible	0.93	1.23	1.49	0.58	0.89	0.81
Q61554	C1847	reversible	1.24	1.73	1.27	1.41	1.39	1.59
Q61554	C2251	reversible	1.01	1.83	1.76	1.42	NA	NA
Q61554	C2413	reversible	1	1.71	1.7	1.2	1.34	1.34
Q61554	C460	reversible	1.05	1.85	1.46	1.45	1.42	1.01
Q61554	C845	reversible	1.2	NA	1.29	1.96	1.11	NA
Q61554	C967	reversible	1.14	1.81	1.63	1.32	1.23	NA
Q61598	C282	reversible	0.94	1.07	1.07	0.86	0.98	0.77
Q61598	C302	reversible	1.21	1.17	1.47	1.48	1.17	1.49
Q61598	C414	reversible	1.24	0.98	1.24	0.65	0.92	NA
Q61702	C265	reversible	1.05	1.63	2	NA	1.48	1.06
Q61738	C546	reversible	1.06	NA	1.01	0.88	0.8	NA
Q61738	C687	reversible	0.88	1.16	1.22	1.02	0.91	1.09
Q61739	C154	reversible	0.77	1.05	1.02	NA	1.13	0.64
Q61739	C881	reversible	1.06	0.91	1.05	NA	0.8	1.13
Q61830	C149	reversible	0.95	1.15	1.4	1.24	1.18	1.07
Q61830	C209	reversible	1.13	1.61	1.83	1.49	1.94	1.39
Q61830	C391	reversible	0.97	1.19	1.27	NA	1.19	1.31
Q61838	C340	reversible	1.03	1.33	1.7	1.26	1.66	0.93
Q61838	C642	reversible	1.1	0.81	1.47	0.81	0.77	0.62
Q61838	C833	reversible	1.12	1.37	1.31	0.91	1.59	1.26
Q63961	C209	reversible	1.21	1.05	NA	1.46	0.95	0.99
Q64669	C180	reversible	0.93	1	NA	1.28	1.14	NA
Q6DIB5	C615	reversible	NA	1.28	1.16	NA	0.87	1.59
Q6IRU2	C154	reversible	1	2.38	1	NA	NA	1.27
Q6NVE9	C130	reversible	1.06	1.1	1.47	1.47	1.32	0.99
Q6P3A8	C226	reversible	0.67	NA	1.09	0.88	0.98	1.07
Q6P3A8	C233	reversible	0.96	1.01	0.96	1.07	1.08	0.83
Q6P3A8	C297	reversible	1	1.91	1.03	1.02	1.33	0.87
Q6P4U0	C864	so2h	0.84	1.09	1.23	0.79	1.06	1.04

Q6P5E4	C159	reversible	0.67	1.13	1.21	NA	1.18	NA
Q6P8J7	C180	reversible	1.11	1.19	1.22	1.08	1.1	1.54
Q6P8J7	C238	reversible	1.14	1.11	1.22	0.91	1.01	1.36
Q6P8J7	C317	reversible	0.98	1.64	1.38	0.92	1.45	1.43
Q6P8J7	C317	so3h	0.87	NA	1.66	NA	1.13	0.79
Q6P8J7	C63	reversible	0.89	0.43	1.08	0.97	NA	0.88
Q6P8J7	C90	reversible	1.16	0.89	1.01	1.13	1.01	1.34
Q6PB66	C1042	reversible	1.14	1.25	1.06	NA	NA	1.14
Q6PB66	C112	reversible	1.28	0.82	0.82	1.11	NA	0.82
Q6PB66	C626	reversible	1.19	NA	0.6	0.92	0.85	1.22
Q6PB66	C847	reversible	0.89	0.91	1.04	0.74	NA	0.93
Q6PDM2	C16	reversible	1.19	1.21	1.58	0.86	1.05	NA
Q6PIE5	C702	reversible	1.06	1.2	1.15	1.03	NA	NA
Q6ZWY3	C77	reversible	1.02	NA	NA	1	1.43	1.01
Q71RI9	C417	reversible	NA	1.09	1.25	0.92	1.75	NA
Q791V5	C49	reversible	0.98	1.11	1.1	0.8	1.17	1.25
Q791V5	C56	reversible	1.01	0.99	1.03	1.04	1.04	1.46
Q791V5	C79	reversible	1.06	1.1	1.2	0.99	1.17	1.63
Q7M760	C104	reversible	NA	1.14	0.86	0.58	1.1	0.75
Q7TMF3	C92	reversible	1.15	0.96	1.05	1.16	1.18	1.29
Q7TMK9	C289	reversible	1.32	1.54	1.14	1.08	NA	NA
Q7TNG5	C605	reversible	0.98	NA	1.14	NA	1.1	1.29
Q7TQ48	C863	reversible	1.12	0.62	1.35	0.64	NA	NA
Q7TQ48	C894	reversible	0.99	0.85	1.27	0.75	1.16	1.29
Q7TQ48	C899	reversible	0.67	0.83	0.62	0.77	0.67	NA
Q7TSQ8	C84	reversible	1.09	1.33	1.11	0.84	NA	1.07
Q80TZ3	C372	reversible	NA	NA	0.7	0.82	0.77	0.75
Q80VJ8	C368	reversible	0.76	1.1	1.95	1.01	1.23	0.72
Q80XL6	C635	so3h	0.83	0.84	NA	0.86	1.26	NA
Q80XN0	C221	reversible	0.68	1.72	0.92	1.58	2.53	2.32
Q80YC5	C47	reversible	0.95	1.03	1.35	1.1	1.08	NA
Q8BFR5	C222	reversible	1.19	1.43	0.95	0.82	1.38	1.26
Q8BFR5	C290	reversible	1.06	1.19	0.95	0.9	1.44	1.09
Q8BGC4	C330	reversible	1.17	1.19	1.24	1.24	NA	0.81
Q8BGH2	C65	reversible	1.03	0.81	0.84	0.84	0.94	0.93
Q8BGK2	C206	reversible	0.99	NA	0.54	0.62	NA	0.86
Q8BH35	C199	reversible	0.66	0.65	0.89	1.2	1.08	1.26
Q8BH59	C435	reversible	0.92	0.82	1.17	NA	1.07	0.74
Q8BH59	C563	reversible	0.95	NA	1.32	0.96	NA	1.27

Q8BH95	C111	reversible	1.22	1.57	1.56	0.59	1.32	1.28
Q8BH95	C225	reversible	1	1.11	1.19	2.62	NA	NA
Q8BHT6	C402	reversible	NA	1.15	0.97	1.38	NA	0.83
Q8BLF1	C345	reversible	0.85	1.01	1.2	1.19	1.05	1.41
Q8BMF3	C450	reversible	NA	0.8	1.02	0.89	1.04	0.92
Q8BMF4	C163	reversible	NA	1.51	0.61	1.07	NA	0.93
Q8BMF4	C290	reversible	1.11	1.17	1.23	0.93	NA	1.54
Q8BMF4	C483	reversible	0.98	1.31	1.21	0.82	1.08	1.24
Q8BMF4	C581	reversible	1.09	0.96	1.16	0.79	1.9	1.66
Q8BMS1	C349	reversible	1.03	1.17	1.34	0.93	1.15	1.15
Q8BMS1	C470	reversible	1.13	1.07	0.92	0.83	0.78	0.97
Q8BMS1	C747	reversible	1.12	1.05	1.29	0.77	1	1.2
Q8BW75	C26	reversible	1.15	0.89	1.34	1.56	NA	1
Q8BWF0	C260	reversible	1	NA	0.74	0.98	NA	1.32
Q8BWF0	C81	reversible	1.12	1.22	0.9	1.05	0.94	1.1
Q8BWT1	C103	reversible	0.77	0.94	1.3	0.91	NA	0.71
Q8BWT1	C107	reversible	0.87	1.08	1.01	0.99	1.61	1.06
Q8BWT1	C287	reversible	0.9	1.24	1.26	0.96	1.52	0.92
Q8BWT1	C92	reversible	1.06	1.12	1.19	0.82	1.1	0.51
Q8BY89	C401	reversible	0.88	NA	1.12	0.55	NA	0.82
Q8C522	C34	reversible	NA	0.85	1.25	1.17	NA	1.15
Q8CEE7	C69	reversible	NA	NA	0.95	0.86	1.49	1.41
Q8CFX1	C376	reversible	1.21	1.4	1.5	1.09	1.07	1.24
Q8CGC7	C1076	reversible	0.91	NA	1	0.81	NA	0.53
Q8CGC7	C697	reversible	1.22	1.12	1.46	0.97	NA	1.22
Q8CGK3	C898	reversible	0.73	1.11	1	0.8	1.09	0.99
Q8CI94	C326	reversible	0.77	NA	1.06	0.93	0.97	1.17
Q8CI94	C373	reversible	1.12	1.07	1.04	0.87	1.21	1.52
Q8CI94	C437	reversible	1.05	1.05	1.03	0.64	1	0.83
Q8CI94	C446	reversible	0.98	1.85	1.03	0.94	1.78	1.28
Q8CI94	C581	reversible	1.68	1.16	1.41	NA	1.16	2.35
Q8JZN5	C275	reversible	1.12	0.98	1.01	0.77	0.85	0.88
Q8JZN5	C617	reversible	1.08	1.17	1.13	0.77	1.13	1.04
Q8JZQ2	C401	reversible	NA	1.23	0.9	0.75	NA	1.12
Q8K010	C408	reversible	1.03	1.04	1.23	1.01	1.37	0.97
Q8K182	C140	reversible	0.93	0.77	0.68	0.85	0.83	0.84
Q8K182	C497	reversible	0.76	1.19	0.65	NA	1.57	NA
Q8K1M6	C367	reversible	1.04	1.36	1.21	NA	1.32	0.98
Q8K1M6	C373	reversible	1.15	0.92	1.04	1.29	1.32	1.01

Q8K1M6	C452	reversible	1.09	0.92	1.23	1.09	1.13	1.24
Q8K1R3	C459	reversible	1.26	1.27	1.04	0.88	NA	1.29
Q8K224	C489	so2h	0.7	1.07	NA	0.42	0.72	NA
Q8K2B3	C189	reversible	0.75	0.9	0.89	NA	1.42	NA
Q8K2B3	C191	reversible	1.16	0.89	1.18	NA	1.16	0.37
Q8K2B3	C238	reversible	1.15	1.05	0.89	0.87	1.21	1.1
Q8K2B3	C266	reversible	1.07	1.15	1.12	0.81	1.18	1.21
Q8K2B3	C357	reversible	NA	0.62	1.07	NA	1.45	0.44
Q8K2B3	C467	reversible	1.04	0.99	1.19	1.1	NA	1.37
Q8K2B3	C475	reversible	1.18	0.93	0.96	0.83	0.94	1.08
Q8K2B3	C536	reversible	1.02	0.88	0.87	0.79	0.83	1.01
Q8K2B3	C654	reversible	1.08	0.94	0.77	0.93	0.86	1.11
Q8K2C6	C293	reversible	1.13	1.1	1.4	1.17	1.34	NA
Q8K3A6	C211	reversible	NA	1.14	0.86	1.54	NA	1
Q8K3J1	C119	reversible	1.22	0.89	1.22	0.82	1.66	1.39
Q8K3J1	C123	reversible	0.93	1.06	1.2	0.98	0.83	1.4
Q8K411	C627	reversible	NA	NA	1.01	1.03	1.04	2.13
Q8K4G1	C599	reversible	1.05	1.37	1.15	NA	1.47	1.1
Q8QZR5	C238	reversible	1.01	1.11	1	1.24	1.33	NA
Q8QZS1	C335	reversible	1.03	0.71	0.91	0.96	1.37	1.09
Q8QZS1	C94	reversible	1	1.26	1.04	0.75	1	1.08
Q8QZT1	C116	reversible	1.1	1.24	1.14	0.8	1.19	1.14
Q8QZT1	C193	reversible	1.17	1.26	0.88	0.55	1.15	1.16
Q8R086	C300	reversible	1.02	1.2	1.05	0.96	1.03	1.58
Q8R086	C509	reversible	1.02	0.88	1.04	NA	0.96	1.29
Q8R0F8	C115	reversible	0.76	1.1	NA	1.06	0.93	1.65
Q8R127	C98	reversible	0.95	1.6	1.23	0.96	2.28	0.8
Q8R4N0	C181	reversible	NA	0.86	1.24	NA	0.84	1.47
Q8R5C5	C34	reversible	1.02	1.31	1.08	0.72	1.12	1.19
Q8VBT0	C106	reversible	1.06	1.13	1.2	1.45	1.08	NA
Q8VCG4	C188	reversible	0.81	NA	0.87	1.21	NA	1.11
Q8VCM7	C34	reversible	1.08	1.38	1.47	1.34	1.38	0.91
Q8VCT4	C284	reversible	1.61	0.54	1.3	0.58	1.42	0.87
Q8VDM4	C448	reversible	NA	1.67	1.22	1.16	NA	1.17
Q8VDN2	C211	reversible	0.93	1.23	1.01	0.77	1	1.13
Q8VDN2	C249	reversible	1.02	1.09	1.37	0.99	1.02	1.02
Q8VDN2	C663	reversible	1.13	NA	0.96	0.58	0.86	1.1
Q8VDN2	C705	reversible	1.28	1.16	1.09	0.91	0.92	0.98
Q8VE95	C100	reversible	1.02	1.4	NA	1.09	NA	0.91

Q8VE96	C254	reversible	0.8	1.14	1.04	1.06	0.87	0.89
Q91VD9	C367	reversible	0.92	0.99	0.82	0.92	0.93	0.98
Q91VD9	C554	reversible	1.01	1.18	1.25	0.95	1.3	1.21
Q91VD9	C75	reversible	1.03	0.98	1.05	0.8	1.06	1.27
Q91VD9	C78	reversible	1.21	1.02	1.18	0.75	1	1.09
Q91VD9	C92	reversible	1.1	1.02	1.12	1.02	1.08	1.47
Q91VR2	C103	reversible	1.09	1.11	1.25	0.9	1.13	1.43
Q91WK1	C114	reversible	1.01	0.78	NA	0.89	0.83	1.41
Q91X72	C148	reversible	0.96	1.24	1.21	1.05	1.31	1.15
Q91X72	C153	reversible	0.87	1.17	1.49	1.21	1.31	0.93
Q91X72	C199	reversible	1.09	0.75	1.44	1.24	0.9	1.03
Q91X72	C230	reversible	1.01	1.25	1.84	1.21	1.49	1.2
Q91X72	C255	reversible	0.68	0.97	1.14	0.83	1.09	0.75
Q91X72	C364	reversible	0.99	0.97	1.61	1.3	1.19	1.01
Q91X72	C406	reversible	0.89	0.8	1.55	1.08	1.11	0.9
Q91X72	C458	reversible	0.92	0.81	1.77	1.26	1.14	1.14
Q91YT0	C125	reversible	0.9	0.97	0.99	0.77	1.08	1.1
Q91YT0	C142	reversible	1	1.15	1.1	0.86	1.33	1.32
Q91YT0	C238	reversible	0.94	1.05	1.39	NA	1.73	1.35
Q91YT0	C255	reversible	1.05	0.89	1.29	NA	1.17	1.1
Q91YT0	C425	reversible	0.98	0.65	0.97	1.2	1.09	NA
Q91YY4	C141	reversible	0.99	1.07	0.91	NA	1.07	1.27
Q91ZA3	C612	reversible	1.22	NA	0.76	0.86	NA	0.93
Q91ZX7	C808	so2h	0.94	1.02	NA	NA	0.83	0.95
Q91ZX7	C815	so2h	NA	1.44	1.01	1.03	NA	0.85
Q921G7	C117	reversible	1.29	0.87	1.01	0.91	0.92	1.22
Q921G7	C265	reversible	1.14	1.05	0.89	0.82	1.03	1.04
Q921G7	C386	reversible	0.99	1	1.08	1.1	1.24	0.96
Q921G7	C585	reversible	0.97	0.72	1.17	0.72	1.09	0.97
Q921G7	C588	reversible	0.92	0.92	1.02	0.81	0.91	0.59
Q921I1	C156	reversible	0.92	1.24	1.54	NA	NA	1.18
Q921I1	C177	reversible	0.94	1.13	1.56	1.68	1.01	1.28
Q921I1	C180	reversible	1.11	1.07	1.49	1.79	1.23	1.02
Q921I1	C246	reversible	0.95	1.22	1.34	1.25	1.06	1.14
Q921I1	C260	reversible	0.79	1.14	1.2	1.14	1	0.97
Q921I1	C28	reversible	0.83	1.03	1.39	1.29	1.2	1.16
Q921I1	C350	reversible	0.86	1.23	1.22	1.18	1.3	1.08
Q921I1	C363	reversible	0.86	1.47	1.84	1.36	NA	1.36
Q921I1	C373	reversible	0.87	0.79	1.15	0.69	0.96	NA

Q921I1	C386	reversible	0.93	1.14	1.21	1.13	1.19	0.95
Q921I1	C472	reversible	0.85	1.32	1.64	1.07	1.17	1.43
Q921I1	C506	reversible	1	1.02	1.26	1.37	0.94	1.14
Q921I1	C583	reversible	0.93	1.18	1.33	1.14	0.89	1.31
Q921I1	C597	reversible	1.08	1.3	1.54	1.21	1.27	1.3
Q921I1	C633	reversible	1.04	0.9	1.91	NA	NA	0.87
Q921I1	C638	reversible	0.9	1.03	1.25	1.54	1.13	1.11
Q921I1	C67	reversible	0.84	1.14	1.42	1.21	1.3	1.5
Q921I1	C692	reversible	0.83	1.01	1.55	1.46	0.81	0.97
Q922B2	C203	reversible	1.11	1.72	1.11	0.62	1.46	0.94
Q922B2	C267	reversible	0.92	1.45	1	1.11	1.26	1.56
Q924M7	C309	reversible	0.98	NA	1.13	1.32	1.32	1.44
Q924X2	C448	reversible	1	1.26	1.07	0.92	1.16	1.21
Q924X2	C526	reversible	0.95	1.18	0.78	0.81	NA	NA
Q924X2	C86	reversible	1.37	1.01	0.64	NA	1.01	0.91
Q99J39	C359	reversible	1.12	0.9	0.82	0.74	0.86	0.66
Q99KI0	C385	reversible	1.01	1.22	1.1	0.75	1.27	1.11
Q99KI0	C451	reversible	1.06	0.97	0.87	0.94	1.3	0.98
Q99LB2	C210	reversible	1.15	0.91	1.5	1.04	1.26	1.5
Q99LC3	C183	reversible	0.97	0.72	0.83	0.91	0.8	1.13
Q99LC3	C67	reversible	1.07	1.15	0.94	0.96	1.23	1.31
Q99LC5	C109	reversible	1.2	1.03	1.27	1.02	1.07	1.3
Q99LC5	C155	reversible	1.1	1.33	1.14	0.74	1.45	1.37
Q99LC5	C53	reversible	1.07	1.31	1.11	0.75	1.22	1.28
Q99LC5	C68	reversible	0.97	1.04	0.97	0.75	1.14	1.09
Q99LX0	C46	reversible	1.32	1.04	1.12	0.67	0.69	NA
Q99LX0	C53	reversible	1.03	0.84	0.71	1.16	NA	NA
Q99LY9	C43	reversible	1.15	1.02	0.82	0.96	0.78	1.11
Q99LY9	C66	reversible	1.02	1.06	0.8	0.81	0.97	1.04
Q99MN9	C518	reversible	NA	NA	0.97	0.35	1.02	0.47
Q99MR8	C186	reversible	NA	0.97	0.94	NA	0.84	0.57
Q99MR8	C450	reversible	1	0.95	1.04	0.68	0.93	NA
Q99MR8	C591	reversible	1.04	0.96	0.8	NA	0.96	NA
Q99NB1	C235	reversible	1.04	1.05	1.13	1.07	1.1	1.32
Q99NB1	C86	reversible	0.82	0.83	1.21	1.18	NA	NA
Q9CPU4	C56	reversible	1.12	1.26	1.16	1.2	0.96	1.67
Q9CPV4	C182	reversible	0.96	0.9	1.03	1.15	1.06	1.21
Q9CPV4	C206	reversible	0.97	0.9	1.14	1.36	1.48	NA
Q9CPY7	C129	reversible	1.09	1.22	1.18	0.88	1.04	1.46

Q9CPY7	C335	reversible	0.87	NA	1.21	0.9	0.99	NA
Q9CPY7	C462	reversible	1.23	1.15	1.21	0.96	1.25	1.35
Q9CQ62	C86	reversible	0.88	1.41	1.55	1.1	NA	1.02
Q9CQN1	C503	reversible	0.69	NA	0.92	1.04	0.49	0.78
Q9CR57	C42	reversible	1.11	0.98	1.61	NA	1.26	NA
Q9CR61	C59	reversible	0.97	0.8	0.96	0.65	NA	NA
Q9CR61	C80	reversible	1.1	1.01	0.98	0.83	1.06	1.23
Q9CR62	C184	reversible	1	1.24	1.54	0.96	0.85	1.73
Q9CRB9	C183	reversible	0.96	0.9	0.83	0.67	0.86	1
Q9CRB9	C193	reversible	0.7	0.82	0.71	0.29	0.88	NA
Q9CRB9	C204	reversible	0.88	0.53	1.22	0.83	NA	1.09
Q9CRB9	C214	reversible	0.95	0.94	0.86	0.96	NA	0.8
Q9CY64	C280	reversible	1.01	1.53	0.85	NA	1.05	1.1
Q9CZ13	C268	reversible	0.97	1.2	1.13	0.96	1.33	1.41
Q9CZ13	C380	reversible	1.02	1.38	1.27	0.88	1.38	1.4
Q9CZ13	C410	reversible	0.93	1.22	0.96	0.96	1.47	1.28
Q9CZ13	C453	reversible	0.95	1.08	1.04	0.87	1.45	1.31
Q9CZ13	C69	reversible	0.89	0.71	1.12	1.03	NA	NA
Q9CZB0	C107	reversible	1.26	1.17	1.19	0.9	1.27	1.46
Q9CZB0	C70	reversible	1.15	1.23	NA	1.05	0.9	NA
Q9CZU6	C101	reversible	0.98	1.21	1.1	0.83	1.34	1.33
Q9D051	C169	reversible	0.69	0.84	0.83	1.04	2.45	NA
Q9D051	C263	reversible	1.04	1.23	0.91	0.85	1.18	1.06
Q9D0F9	C101	reversible	1.07	1	1.3	1.24	1.31	NA
Q9D0F9	C160	reversible	0.96	0.88	1.11	0.82	NA	1.23
Q9D0G0	C196	reversible	1.01	0.93	NA	1.36	0.88	NA
Q9D0K2	C456	reversible	0.75	1.02	1.11	0.98	1.34	NA
Q9D172	C174	reversible	1.13	1.1	0.65	1.1	0.66	1.01
Q9D1Q6	C189	reversible	0.91	0.98	1.05	1.09	1.02	1.26
Q9D1Q6	C301	reversible	0.69	0.82	1.08	0.86	0.98	1.05
Q9D1Q6	C318	reversible	0.99	1.14	NA	1.04	1.2	1.34
Q9D404	C86	reversible	1.1	0.83	0.94	1.11	0.97	NA
Q9D5T0	C137	reversible	0.88	1.01	1.66	1.2	NA	NA
Q9D5T0	C310	reversible	1.05	1.07	1.05	0.95	NA	1.88
Q9D6R2	C222	reversible	0.88	0.84	0.87	0.85	0.67	1.05
Q9D6R2	C331	reversible	1.08	1	0.99	0.82	0.86	1.28
Q9D6R2	C359	reversible	0.89	0.96	1.1	0.71	0.78	0.79
Q9D7B6	C215	reversible	1.22	1.38	1.04	0.9	1.29	1.27
Q9D7B6	C281	reversible	NA	NA	0.78	1.03	1.12	1.28

Q9D7N3	C227	reversible	NA	1.23	0.92	0.85	NA	0.74
Q9D7N9	C148	reversible	1.18	1.18	1.82	0.9	1.38	NA
Q9D8B4	C18	reversible	0.88	1.03	0.9	0.78	1.11	1.05
Q9D8B4	C95	reversible	0.83	0.96	0.91	0.82	1.01	1.25
Q9DB20	C141	reversible	1.18	1.13	1.27	0.66	1.21	1.46
Q9DB77	C192	reversible	1.1	1.6	1.01	0.88	1.28	1.26
Q9DBD0	C260	reversible	0.83	1.46	NA	1.14	NA	0.75
Q9DBD0	C58	reversible	1	1.16	1.91	1.29	1.56	NA
Q9DBD0	C67	reversible	1.1	1.22	1.19	1.07	NA	NA
Q9DBL1	C261	reversible	0.98	NA	0.94	NA	1.15	1.37
Q9DC69	C86	reversible	0.85	1.05	1.03	0.98	1.58	0.95
Q9DCJ5	C110	reversible	0.99	0.91	0.83	0.68	1	1.12
Q9DCJ5	C36	reversible	1	0.98	1.04	0.8	1.07	1.28
Q9DCJ5	C66	reversible	0.99	1.44	1.02	0.75	1.62	1.56
Q9DCL9	C281	reversible	1.04	1.36	1.22	NA	1.35	0.94
Q9DCS9	C125	reversible	0.99	1.09	1.15	0.83	1.09	1.12
Q9DCS9	C77	reversible	1.12	1.13	0.9	0.96	1.17	1.27
Q9DCW4	C66	reversible	1	1.09	1.17	0.79	1.25	1.1
Q9DCW4	C71	reversible	1.07	0.95	1.47	0.75	1.07	1.13
Q9DCX2	C101	reversible	0.73	2.04	1.64	0.81	0.82	1.64
Q9DCZ1	C186	reversible	1.07	NA	1.09	1.09	1.25	1.21
Q9EQ20	C368	reversible	NA	1.28	1.25	0.88	NA	1.38
Q9EQ20	C86	reversible	1.13	0.88	0.64	1.07	0.68	1.05
Q9EQH3	C156	reversible	1.37	NA	NA	1	1.21	1.33
Q9ESB3	C103	reversible	0.83	1.39	1.64	1.39	NA	NA
Q9ESB3	C216	reversible	0.97	1.55	1.41	1.53	1.14	1.39
Q9ESB3	C89	reversible	0.96	1.45	1.31	1.68	NA	NA
Q9ESW4	C72	reversible	0.88	1.52	1.3	1.15	1	0.94
Q9JHI5	C134	reversible	0.99	0.97	1.03	1.2	1.07	0.77
Q9JHI5	C252	reversible	1	0.81	1.02	0.77	0.86	0.98
Q9JHI5	C348	reversible	1.09	1.04	0.91	0.51	1.22	1.03
Q9JHW2	C44	reversible	0.78	0.88	NA	1.3	0.93	1.05
Q9JHW2	C97	reversible	0.9	0.79	1.04	1.25	NA	NA
Q9JI91	C187	reversible	0.68	1.91	1.38	1.38	2.14	0.97
Q9QUI0	C16	reversible	1.13	1.26	1.17	1.57	1.01	1.31
Q9QUR6	C57	reversible	1.21	1.37	1.34	1.55	1.4	1.18
Q9QWK4	C194	reversible	1.08	1.19	0.84	1.03	1.92	1.18
Q9QXX4	C504	reversible	0.96	1.04	1.3	1.22	NA	1.01
Q9QY15	C320	reversible	0.97	1.35	NA	NA	1.35	1.06

Q9QYG0	C78	reversible	0.76	1.35	1.1	0.69	NA	NA
Q9QZZ6	C50	reversible	1.05	0.63	1	0.7	NA	0.79
Q9R069	C118	reversible	0.96	1.17	0.89	0.78	1.11	0.72
Q9R069	C284	reversible	0.97	NA	1.22	0.81	NA	0.74
Q9R069	C516	reversible	0.95	1	1.23	0.74	0.8	1
Q9R0H0	C199	reversible	1.18	0.82	0.86	0.63	NA	0.62
Q9WTP6	C42	reversible	0.98	0.46	1.08	0.87	NA	NA
Q9WTP6	C92	reversible	0.99	1.06	NA	0.57	NA	0.73
Q9WUB3	C446	reversible	0.94	1.48	1.1	1.03	1.52	1.22
Q9WUB3	C496	reversible	1.57	1.14	1.16	1.2	1.4	1.27
Q9WUM5	C172	reversible	0.99	0.97	1.07	1.16	1.92	1
Q9WUM5	C181	reversible	0.82	1.03	0.93	1.61	0.87	1.01
Q9WUM5	C60	reversible	0.96	1	1.11	1.32	1.27	1.04
Q9WUR2	C279	reversible	1.07	NA	1.29	1.1	0.66	NA
Q9WVH9	C46	reversible	0.97	0.85	NA	NA	0.86	0.77
Q9Z0P5	C275	reversible	0.98	0.87	1.23	NA	1.17	NA
Q9Z0P5	C67	reversible	0.99	0.95	1.09	1.55	1.3	NA
Q9Z1Q2	C7	reversible	0.98	NA	1.48	1.35	1.23	NA
Q9Z2C5	C482	reversible	1.22	1.15	NA	1.46	0.88	NA
Q9Z2I9	C152	reversible	1.01	1.31	0.87	0.8	0.9	1.19
Q9Z2I9	C158	reversible	1.11	1.14	0.85	0.81	0.9	1.2
Q9Z2I9	C270	reversible	1.41	0.92	0.9	NA	NA	0.89
Q9Z2I9	C430	reversible	1.04	1.02	0.77	0.68	0.85	1
Q9Z2Z6	C155	reversible	1	1.18	0.51	NA	1.15	NA
A2A8L5	C1527	so3h	0.77	NA	NA	0.97	NA	1.56
A2A8L5	C1539	so2h	0.77	NA	NA	0.97	NA	1.56
A2AUC9	C147	reversible	1.04	NA	1.19	1.22	NA	NA
B2RXS4	C617	reversible	0.92	NA	0.95	0.8	NA	NA
E9PV24	C438	reversible	1.06	0.94	NA	NA	NA	0.72
O08529	C374	reversible	1.18	1.39	1.11	NA	NA	NA
O35600	C1487	so2h	0.81	NA	1.24	NA	NA	1.23
O35600	C1489	so2h	0.81	NA	1.24	NA	NA	1.23
O55143	C344	reversible	NA	NA	0.73	0.93	0.83	NA
O55143	C498	reversible	NA	1.22	0.6	1	NA	NA
O70370	C178	reversible	NA	1.6	0.79	1.05	NA	NA
O70468	C1260	reversible	0.85	NA	NA	0.78	NA	0.99
O88737	C1775	so2h	1.17	NA	0.76	1.16	NA	NA
O88792	C49	reversible	1.03	NA	1.29	1.29	NA	NA
O89103	C297	reversible	1	0.85	NA	0.45	NA	NA

P01029	C667	reversible	2.32	1.08	NA	3.38	NA	NA
P01872	C304	reversible	1.27	NA	1.1	0.85	NA	NA
P01872	C352	reversible	0.88	NA	1.22	NA	NA	0.89
P02463	C1493	reversible	1.1	0.94	1.33	NA	NA	NA
P02468	C475	reversible	NA	NA	1.46	0.63	0.98	NA
P06801	C47	reversible	1.09	1.01	1.26	NA	NA	NA
P06909	C205	so3h	NA	NA	NA	0.69	0.94	0.78
P06909	C210	so2h	NA	NA	NA	1.07	0.94	0.78
P07724	C384	reversible	NA	0.96	NA	1.09	NA	1.45
P07724	C385	reversible	NA	0.96	NA	1.09	NA	1.45
P08607	C245	reversible	1.42	1.54	NA	2.23	NA	NA
P09055	C618	reversible	NA	NA	1.54	0.43	NA	1.89
P09411	C50	reversible	NA	1.31	NA	0.95	NA	1.37
P10605	C319	reversible	0.8	0.87	0.99	NA	NA	NA
P10751	C9	reversible	1.46	0.86	NA	1.12	NA	NA
P16015	C187	reversible	2.27	NA	1.65	1.09	NA	NA
P19221	C236	reversible	1.06	NA	1.38	1.18	NA	NA
P19221	C547	reversible	NA	0.81	1.02	1.31	NA	NA
P20918	C324	reversible	1.37	1.6	1	NA	NA	NA
P20918	C609	reversible	0.99	1.08	0.72	NA	NA	NA
P20918	C625	reversible	0.99	1.08	0.72	NA	NA	NA
P21981	C553	reversible	NA	NA	0.78	1.02	NA	0.71
P22315	C193	reversible	NA	1.55	NA	0.75	NA	1.1
P26041	C117	reversible	1.31	1.79	NA	NA	NA	1.38
P28271	C392	reversible	1.12	1.3	NA	NA	NA	1.6
P28474	C97	reversible	1.44	1.01	NA	0.91	NA	NA
P34914	C312	reversible	NA	0.74	1.18	NA	0.67	NA
P34914	C521	reversible	1.04	NA	0.83	0.91	NA	NA
P35700	C71	reversible	0.89	0.73	NA	1.38	NA	NA
P35922	C141	so2h	NA	NA	0.73	0.99	1.03	NA
P41183	C636	reversible	0.89	NA	0.95	NA	NA	1.37
P42125	C87	reversible	0.87	NA	1.02	NA	0.78	NA
P45376	C299	reversible	NA	NA	0.64	1.63	0.97	NA
P45377	C83	so3h	NA	0.94	NA	NA	1.24	0.84
P50396	C202	reversible	1.21	1.6	1.19	NA	NA	NA
P50462	C168	reversible	1.17	NA	0.7	1.22	NA	NA
P50462	C25	reversible	NA	NA	1.18	0.82	NA	1.26
P50544	C423	reversible	NA	0.96	1.25	1.19	NA	NA
P51881	C257	reversible	1.93	1.09	NA	0.69	NA	NA

P52503	C104	reversible	0.87	NA	1.34	1.12	NA	NA
P54797	C135	reversible	0.99	NA	1.49	NA	1.27	NA
P56391	C65	reversible	1.12	NA	1.9	0.85	NA	NA
P62281	C60	reversible	1.13	NA	1.56	1.18	NA	NA
P62827	C120	reversible	NA	2.26	NA	1.85	NA	1.61
P62880	C148	reversible	0.86	1.21	NA	0.82	NA	NA
P63011	C184	reversible	0.51	NA	1.05	1.26	NA	NA
P67778	C69	reversible	1.19	1.1	1.28	NA	NA	NA
P97351	C139	reversible	NA	1.89	1.78	NA	1.16	NA
Q00724	C192	reversible	1.43	NA	1.63	1.18	NA	NA
Q03265	C294	reversible	0.99	0.9	NA	NA	0.81	NA
Q04998	C38	reversible	NA	1.07	NA	NA	1.07	1.21
Q05920	C739	reversible	NA	1.22	0.91	1.08	NA	NA
Q07113	C1509	reversible	0.92	NA	NA	0.95	0.83	NA
Q07113	C2241	reversible	0.92	1.19	NA	1.63	NA	NA
Q07968	C379	reversible	NA	1.8	1.4	NA	1.76	NA
Q08857	C313	reversible	NA	1.34	NA	0.87	NA	1.09
Q2TL60	C331	reversible	NA	NA	1.21	1.1	NA	1.34
Q2VLH6	C591	reversible	1.35	1.09	0.91	NA	NA	NA
Q3THS6	C34	reversible	1.82	NA	1.16	NA	NA	1.37
Q3TMH2	C143	reversible	1.01	2.41	1.28	NA	NA	NA
Q3TXS7	C898	reversible	NA	NA	1.31	1.08	NA	1.17
Q3UX10	C323	reversible	0.59	NA	0.99	NA	1.17	NA
Q3V384	C72	reversible	NA	1.24	1.15	NA	NA	1.12
Q60675	C3115	reversible	NA	1.15	NA	NA	1.15	0.76
Q60930	C104	reversible	1.05	1.18	NA	NA	NA	1.21
Q61147	C173	reversible	0.74	NA	NA	NA	1.23	1.21
Q61543	C114	reversible	0.68	1.11	NA	0.72	NA	NA
Q61554	C1182	reversible	1.46	NA	1.56	NA	NA	1.85
Q61554	C2489	reversible	0.89	1.46	1.34	NA	NA	NA
Q61738	C103	reversible	NA	0.46	0.63	0.85	NA	NA
Q61838	C1339	reversible	1.01	NA	1.38	NA	NA	0.62
Q61838	C249	reversible	1.5	0.93	NA	1.02	NA	NA
Q61838	C689	reversible	NA	0.63	1.72	NA	1.21	NA
Q61838	C859	reversible	1.23	1.25	1.2	NA	NA	NA
Q61838	C861	reversible	1.23	1.2	NA	NA	NA	1.11
Q62188	C248	reversible	0.73	NA	2.1	1.57	NA	NA
Q64112	C63	so2h	NA	1.39	0.95	NA	NA	0.88
Q6GYP7	C1152	reversible	NA	0.63	0.73	0.98	NA	NA

Q6PIE5	C372	reversible	1.16	NA	0.85	0.83	NA	NA
Q6PIE5	C426	reversible	1	0.75	NA	0.88	NA	NA
Q6X893	C97	reversible	0.72	NA	1.28	NA	0.8	NA
Q6ZWV3	C105	reversible	1.21	NA	1.74	1.35	NA	NA
Q80V23	C162	reversible	1.38	1.12	1.25	NA	NA	NA
Q80YQ8	C4	reversible	NA	1.12	NA	NA	1.27	1.21
Q8BFP9	C238	reversible	1.04	NA	NA	0.95	NA	0.82
Q8BGK2	C23	reversible	NA	1.31	0.77	0.92	NA	NA
Q8BH35	C502	reversible	0.83	NA	0.76	1.03	NA	NA
Q8BH86	C54	reversible	1.24	NA	0.74	0.91	NA	NA
Q8BJU9	C267	reversible	NA	1.07	1.09	NA	NA	0.8
Q8BKY8	C12	so2h	1.15	NA	0.99	NA	0.81	NA
Q8C7H1	C97	reversible	NA	0.99	1.03	1.28	NA	NA
Q8C7X2	C339	reversible	0.94	NA	NA	1.37	NA	0.72
Q8CC88	C415	reversible	NA	NA	0.87	0.88	NA	0.81
Q8CFX1	C390	reversible	NA	0.79	1.01	NA	1.25	NA
Q8CFX1	C621	reversible	NA	0.79	0.65	0.77	NA	NA
Q8CGK3	C509	reversible	NA	1.2	0.71	NA	0.97	NA
Q8JZQ2	C312	reversible	1.01	0.86	0.72	NA	NA	NA
Q8K182	C194	reversible	0.82	0.74	NA	NA	0.74	NA
Q8K2B3	C311	reversible	1.86	0.95	0.59	NA	NA	NA
Q8K2C6	C181	reversible	NA	NA	1.25	0.86	0.96	NA
Q8K3A6	C209	reversible	NA	NA	1.01	0.72	NA	0.77
Q8K4G1	C417	reversible	NA	1.05	1.33	0.9	NA	NA
Q8QZS1	C44	reversible	NA	1.4	1.14	0.87	NA	NA
Q8QZT1	C123	reversible	NA	NA	1.21	0.88	NA	0.99
Q8R2Q0	C420	reversible	0.92	NA	1.22	NA	1.23	NA
Q8R3F9	C577	reversible	1.02	NA	0.79	NA	NA	1.1
Q8R4N0	C72	reversible	1.11	0.73	NA	NA	NA	1.27
Q8VBT1	C275	reversible	0.68	1.19	NA	NA	1.1	NA
Q8VDQ1	C187	reversible	1.01	NA	0.79	0.98	NA	NA
Q8VEM8	C131	reversible	0.72	1	NA	1.35	NA	NA
Q91W90	C366	reversible	NA	1.33	1.71	NA	NA	1.21
Q91YK8	C153	reversible	NA	NA	1.28	0.97	0.99	NA
Q91ZJ5	C123	reversible	1.05	1.39	1.4	NA	NA	NA
Q91ZX7	C3696	reversible	NA	NA	1.86	1.25	1.33	NA
Q922B1	C197	reversible	NA	1.81	NA	0.72	NA	0.71
Q924D0	C130	reversible	0.85	NA	1.2	NA	NA	0.7
Q924X2	C155	reversible	1.22	NA	1.19	1.03	NA	NA

Q99J39	C214	reversible	1.07	0.7	0.87	NA	NA	NA
Q99J39	C67	reversible	1	0.52	0.64	NA	NA	NA
Q99JY3	C61	reversible	NA	NA	1.2	0.89	NA	1.47
Q99M04	C210	reversible	0.67	1.23	1.51	NA	NA	NA
Q99M87	C292	reversible	1.1	1.26	NA	0.95	NA	NA
Q99MN9	C367	reversible	1.12	2.39	NA	0.76	NA	NA
Q99MN9	C519	reversible	0.83	NA	1.35	0.49	NA	NA
Q99P30	C223	reversible	1.25	NA	1.46	0.84	NA	NA
Q9CPU0	C139	reversible	1.01	NA	1.59	1.05	NA	NA
Q9CPY7	C376	reversible	1.05	1.74	NA	1.09	NA	NA
Q9CQJ8	C42	reversible	NA	NA	NA	1.66	1.42	1.37
Q9CZ30	C75	reversible	NA	1.22	3.02	1.25	NA	NA
Q9D0S9	C75	reversible	1.15	NA	1.35	NA	NA	1.1
Q9D5T0	C359	reversible	0.95	NA	1.28	1.08	NA	NA
Q9D6R2	C351	reversible	NA	1.94	0.58	NA	NA	1.25
Q9D7B6	C249	reversible	0.84	NA	0.51	NA	NA	0.92
Q9D7J4	C28	reversible	1.13	0.74	NA	0.7	NA	NA
Q9D7N9	C139	reversible	0.98	NA	1.87	1.01	NA	NA
Q9D8N0	C194	reversible	NA	1.61	NA	1.49	NA	1.56
Q9DBL1	C234	reversible	1.8	NA	NA	0.98	NA	1.7
Q9DC70	C194	reversible	0.74	0.62	NA	NA	NA	1.05
Q9DCM0	C219	reversible	0.82	1.2	1.24	NA	NA	NA
Q9DCM0	C34	reversible	1	0.94	NA	1.2	NA	NA
Q9ET30	C64	reversible	1.35	NA	2.05	1.37	NA	NA
Q9JI91	C48	reversible	0.9	NA	NA	1.48	2.4	NA
Q9QXX4	C566	reversible	1.22	NA	0.95	1.11	NA	NA
Q9QYJ0	C146	reversible	NA	1.34	NA	0.98	1.34	NA
Q9QZ88	C41	reversible	1	NA	1.1	1.28	NA	NA
Q9R069	C47	reversible	NA	1.13	1.32	0.82	NA	NA
Q9R0P9	C220	reversible	0.98	2.58	NA	1.4	NA	NA
Q9Z0X1	C316	reversible	0.66	NA	NA	NA	0.83	1.09
Q9Z2W0	C445	reversible	NA	2.25	NA	1.01	1.19	NA
Q9Z2Z6	C58	reversible	0.76	0.67	NA	1.11	NA	NA
P08122	C1653	reversible	1.22	1.25	1.27	1.3	1.34	1.39
P08122	C1660	reversible	2.23	2.01	1.79	1.57	1.24	0.79
P13542	C950	reversible	0.87	0.84	0.82	0.79	0.75	0.7
P16125	C164	reversible	1.31	1.27	1.24	1.2	1.14	1.07
Q61292	C50	reversible	0.58	0.62	0.66	0.71	0.77	0.86
Q61835	C61	so3h	0.73	0.74	0.75	0.76	0.77	0.79

Q8C547	C870	so2h	5.28	4.48	3.67	2.87	1.67	0.06
Q9JKS4	C594	reversible	0.64	0.67	0.71	0.75	0.81	0.89
A2ARA8	C933	so2h	1.17	1.02	2.58	3.54	4.98	6.99
E9Q394	C1380	so3h	0.79	0.57	0.54	0.44	0.29	0.1
O54734	C130	reversible	0.97	1.28	2.6	3.56	5.01	6.99
O88516	C415	reversible	1.06	1.06	2.55	3.52	4.97	6.99
P16332	C469	reversible	1.02	0.69	0.67	0.54	0.35	0.1
P17710	C214	reversible	1.1	1.33	2.66	3.61	5.03	6.99
P20918	C73	reversible	1.55	1.51	2.91	3.8	5.13	6.99
P21956	C308	reversible	0.83	1.6	2.66	3.62	5.06	6.99
Q06806	C325	reversible	0.96	1.19	2.56	3.53	4.99	6.99
Q3UHN9	C6	so2h	0.79	0.83	0.63	0.52	0.35	0.1
Q60864	C62	reversible	0.91	0.8	0.67	0.54	0.36	0.1
Q61129	C62	so3h	1.38	0.84	2.6	3.55	4.97	6.99
Q61129	C64	so3h	1.38	0.84	2.6	3.55	4.97	6.99
Q6TL19	C605	reversible	0.94	0.87	0.7	0.57	0.37	0.1
Q6TL19	C608	reversible	0.94	0.87	0.7	0.57	0.37	0.1
Q80V03	C321	so3h	1.01	0.86	0.73	0.59	0.38	0.1
Q8BFZ8	C12	so3h	1.36	0.63	0.78	0.62	0.38	0.1
Q8BGA8	C10	so3h	1.06	1.01	2.53	3.51	4.96	6.99
Q8VCM7	C48	reversible	1.13	0.9	2.52	3.49	4.95	6.99
Q9CR55	C19	so3h	1.04	1.03	2.53	3.5	4.96	6.99
Q9CZ49	C559	so3h	0.92	0.94	0.72	0.59	0.39	0.1
Q9DCJ5	C78	reversible	1.38	0.57	0.77	0.61	0.37	0.1
A2ARA8	C923	so2h	1.17	1.02	3.56	6.99	6.99	6.99
O35660	C87	reversible	0.99	1	0.62	0.1	0.1	0.1
O70325	C16	so2h	1.1	0.88	0.62	0.1	0.1	0.1
P06909	C906	reversible	1.22	0.99	3.57	6.99	6.99	6.99
P62897	C18	so2h	1.06	0.72	0.57	0.1	0.1	0.1
P63038	C442	reversible	1.67	1.12	3.75	6.99	6.99	6.99
Q5SUV1	C56	reversible	1.08	0.96	3.52	6.99	6.99	6.99
Q60766	C373	so3h	0.41	0.59	0.33	0.1	0.1	0.1
Q61129	C57	so2h	1.38	0.84	3.58	6.99	6.99	6.99
Q61316	C417	reversible	1.06	2.37	3.9	6.99	6.99	6.99
Q61702	C537	reversible	0.84	0.76	0.51	0.1	0.1	0.1
Q6PDM2	C148	reversible	1.22	1.03	3.58	6.99	6.99	6.99
Q80W21	C87	reversible	1.11	1	3.54	6.99	6.99	6.99
Q8BGA8	C20	so3h	1.06	1.01	3.53	6.99	6.99	6.99
Q8BH59	C505	reversible	1.04	0.88	0.61	0.1	0.1	0.1

Q8BPB5	C298	reversible	0.92	1.11	3.51	6.99	6.99	6.99
Q8BPB5	C48	reversible	0.77	1.13	0.59	0.1	0.1	0.1
Q8CFX1	C83	reversible	1.2	1.56	3.72	6.99	6.99	6.99
Q91VM9	C156	reversible	0.64	1.27	0.59	0.1	0.1	0.1
Q99J39	C31	so3h	1.04	1.17	3.56	6.99	6.99	6.99
Q9D051	C161	reversible	0.9	1.19	3.53	6.99	6.99	6.99
Q9JKB1	C95	reversible	1.35	1.22	3.67	6.99	6.99	6.99
Q9Z1K6	C163	reversible	1	0.91	0.6	0.1	0.1	0.1
O88799	C3240	so2h	0.1	0.36	0.48	0.61	1.21	0.76
P49817	C156	so2h	6.99	5.64	4.67	3.69	1.02	1.11
Q05793	C1140	reversible	6.99	5.66	4.69	3.73	1.08	1.2
Q5M956	C140	reversible	0.1	0.33	0.46	0.59	1.07	0.87
Q6PFY8	C537	reversible	6.99	5.83	4.99	4.16	1.84	1.97
Q8K2A7	C571	so2h	0.1	0.25	0.4	0.56	0.76	1.11
P01942	C105	reversible	6.99	6.99	6.99	4.58	1.27	1.69
P49817	C143	so2h	6.99	6.99	6.99	4.39	1.02	1.11
Q69ZK6	C1674	so3h	0.1	0.1	0.1	0.43	1.36	0.35
Q8BWF0	C328	reversible	6.99	6.99	6.99	4.37	0.9	1.12
Q9CXW4	C72	reversible	6.99	6.99	6.99	4.48	1.13	1.4
Q9DB20	C141	so3h	6.99	6.99	6.99	4.37	0.68	1.34
A1L317	C3	so3h	0.69	0.6	0.51	0.42	0.29	0.1
A2AHJ4	C1000	reversible	1.01	1.93	2.85	3.77	5.15	6.99
A2ARZ3	C2049	so2h	0.67	0.58	0.49	0.41	0.28	0.1
A2AVA0	C3004	so2h	1.61	2.43	3.26	4.09	5.33	6.99
A2AVA0	C3550	so2h	1.14	2.04	2.94	3.84	5.19	6.99
A4Q9E5	C922	so3h	0.74	0.64	0.54	0.45	0.3	0.1
A6H584	C1974	so2h	0.96	0.83	0.7	0.56	0.37	0.1
B1AY10	C575	reversible	1.4	2.26	3.12	3.98	5.27	6.99
D2J0Y4	C463	so2h	0.88	0.76	0.64	0.52	0.34	0.1
F7A4A7	C1907	so3h	1.35	2.21	3.08	3.95	5.25	6.99
F7A4A7	C753	reversible	1.21	2.1	2.99	3.88	5.21	6.99
G3X912	C76	so2h	1.41	2.27	3.12	3.98	5.27	6.99
O08550	C1255	so3h	1.28	2.16	3.04	3.92	5.23	6.99
O35309	C183	reversible	0.8	0.69	0.58	0.48	0.32	0.1
O35516	C1344	so2h	1.4	2.26	3.12	3.98	5.27	6.99
O35516	C1349	so2h	1.4	2.26	3.12	3.98	5.27	6.99
O35516	C410	reversible	1.15	2.05	2.95	3.85	5.19	6.99
O35598	C436	reversible	1.06	1.98	2.89	3.8	5.17	6.99
O35643	C123	so2h	0.95	0.82	0.69	0.56	0.37	0.1

O35714	C45	reversible	0.97	0.84	0.7	0.57	0.37	0.1
O35887	C13	so3h	1.1	2.01	2.91	3.82	5.18	6.99
O35914	C700	so3h	1.28	2.16	3.04	3.92	5.23	6.99
O55222	C8	so2h	0.94	0.81	0.68	0.55	0.36	0.1
O88342	C225	so2h	1.06	1.97	2.88	3.8	5.16	6.99
P01029	C1721	reversible	0.79	0.69	0.58	0.47	0.32	0.1
P06909	C114	reversible	1.01	1.93	2.85	3.77	5.15	6.99
P06909	C205	reversible	0.45	0.4	0.34	0.29	0.21	0.1
P06909	C850	reversible	1.47	2.32	3.17	4.02	5.29	6.99
P07759	C260	reversible	1.57	2.41	3.24	4.07	5.32	6.99
P08607	C329	reversible	0.89	0.77	0.65	0.53	0.35	0.1
P09528	C131	reversible	1.39	2.25	3.11	3.97	5.27	6.99
P09542	C85	reversible	1.05	1.96	2.88	3.79	5.16	6.99
P10493	C409	reversible	0.92	0.8	0.67	0.54	0.36	0.1
P11152	C266	reversible	1.06	1.97	2.88	3.8	5.17	6.99
P11276	C136	reversible	1.31	2.18	3.06	3.93	5.24	6.99
P11881	C1646	so3h	2.09	2.85	3.6	4.35	5.48	6.99
P15105	C99	reversible	1.58	2.41	3.25	4.08	5.33	6.99
P16858	C150	so2h	1	0.86	0.72	0.59	0.38	0.1
P16858	C154	so3h	1	0.86	0.72	0.59	0.38	0.1
P17534	C80	so2h	2.11	2.86	3.61	4.36	5.49	6.99
P17534	C86	so2h	0.69	0.6	0.51	0.42	0.29	0.1
P17534	C86	so3h	0.91	0.79	0.67	0.54	0.35	0.1
P17710	C890	so3h	0.4	0.36	0.31	0.27	0.2	0.1
P25118	C99	so3h	0.86	0.74	0.63	0.51	0.34	0.1
P26041	C284	reversible	0.88	0.76	0.64	0.52	0.34	0.1
P28078	C105	so2h	0.93	0.8	0.67	0.55	0.36	0.1
P28825	C675	so2h	0.81	0.7	0.59	0.48	0.32	0.1
P28825	C680	so2h	0.81	0.7	0.59	0.48	0.32	0.1
P29788	C179	so2h	1.02	1.94	2.86	3.78	5.15	6.99
P31938	C277	reversible	0.78	0.67	0.57	0.47	0.31	0.1
P37242	C36	so2h	1.06	1.98	2.89	3.8	5.17	6.99
P37242	C93	so3h	0.97	0.84	0.7	0.57	0.37	0.1
P47791	C355	reversible	0.77	0.67	0.57	0.47	0.31	0.1
P52431	C1013	reversible	0.65	0.57	0.48	0.4	0.27	0.1
P52480	C317	so3h	0.95	0.82	0.69	0.56	0.37	0.1
P54265	C217	so2h	0.82	0.71	0.6	0.49	0.33	0.1
P55066	C252	so2h	0.88	0.76	0.64	0.52	0.34	0.1
P56400	C30	reversible	0.78	0.68	0.57	0.47	0.31	0.1

P58459	C1101	so2h	0.92	0.79	0.67	0.54	0.36	0.1
P61407	C1227	so3h	0.79	0.68	0.58	0.47	0.32	0.1
P63260	C217	so3h	0.95	0.82	0.69	0.56	0.36	0.1
P68040	C138	reversible	1.16	2.06	2.96	3.85	5.2	6.99
P70398	C2238	so3h	1.07	1.98	2.89	3.8	5.17	6.99
P70398	C2239	so3h	1.07	1.98	2.89	3.8	5.17	6.99
P80314	C395	reversible	0.83	0.72	0.61	0.5	0.33	0.1
P81183	C121	reversible	0.87	0.76	0.64	0.52	0.34	0.1
P97315	C143	so3h	0.66	0.58	0.49	0.41	0.28	0.1
P97315	C149	so2h	0.66	0.58	0.49	0.41	0.28	0.1
P97412	C1983	reversible	1.01	1.93	2.85	3.77	5.15	6.99
P97447	C276	reversible	1.12	2.02	2.92	3.83	5.18	6.99
P97479	C1864	so3h	1.19	2.08	2.97	3.87	5.21	6.99
P97479	C628	so2h	0.92	0.8	0.67	0.55	0.36	0.1
P97479	C705	so3h	0.81	0.7	0.59	0.48	0.32	0.1
P98156	C400	reversible	1.1	2	2.91	3.82	5.18	6.99
Q08857	C311	so3h	1.39	2.25	3.11	3.97	5.27	6.99
Q08857	C313	so2h	1.39	2.25	3.11	3.97	5.27	6.99
Q1XH17	C86	reversible	1.31	2.18	3.06	3.93	5.24	6.99
Q2TJ95	C105	so3h	1.77	2.57	3.37	4.18	5.38	6.99
Q2VLH6	C364	reversible	1.05	1.96	2.88	3.79	5.16	6.99
Q3TXS7	C571	reversible	1.38	2.25	3.11	3.97	5.27	6.99
Q3U492	C529	reversible	1	0.86	0.72	0.59	0.38	0.1
Q3UDW8	C533	so2h	1.02	1.94	2.86	3.78	5.15	6.99
Q3UH93	C1592	reversible	1.19	2.08	2.98	3.87	5.21	6.99
Q3UND0	C190	reversible	0.94	0.81	0.68	0.56	0.36	0.1
Q3URK3	C592	reversible	1.07	1.98	2.89	3.8	5.17	6.99
Q497K7	C204	so3h	0.86	0.75	0.63	0.51	0.34	0.1
Q4U2R1	C3880	so3h	0.92	0.8	0.67	0.55	0.36	0.1
Q52KF3	C547	so2h	0.64	0.56	0.48	0.39	0.27	0.1
Q5BKS4	C118	so2h	1.49	2.34	3.18	4.03	5.3	6.99
Q5BKS4	C234	so3h	1.02	1.94	2.86	3.78	5.15	6.99
Q5DU57	C267	reversible	0.88	0.76	0.64	0.52	0.34	0.1
Q5DU57	C274	reversible	0.88	0.76	0.64	0.52	0.34	0.1
Q5NCS9	C361	so2h	1.03	1.95	2.86	3.78	5.16	6.99
Q5NCS9	C364	so2h	1.03	1.95	2.86	3.78	5.16	6.99
Q5SPV6	C19	so3h	0.68	0.59	0.51	0.42	0.28	0.1
Q60675	C784	reversible	1.12	2.02	2.93	3.83	5.18	6.99
Q60675	C831	reversible	0.89	0.77	0.65	0.53	0.35	0.1

Q60766	C371	so2h	0.41	0.36	0.32	0.27	0.2	0.1
Q60864	C282	reversible	0.69	0.6	0.51	0.42	0.28	0.1
Q60930	C9	so3h	1.73	2.54	3.35	4.16	5.37	6.99
Q61147	C275	reversible	0.99	0.85	0.71	0.58	0.38	0.1
Q61543	C400	reversible	0.62	0.54	0.46	0.38	0.26	0.1
Q61554	C494	reversible	1.77	2.57	3.38	4.18	5.38	6.99
Q61555	C512	so3h	0.02	0.04	0.05	0.06	0.08	0.1
Q61792	C35	so2h	0.83	0.72	0.61	0.5	0.33	0.1
Q61830	C316	reversible	1.55	2.39	3.23	4.06	5.32	6.99
Q62443	C89	reversible	0.96	0.83	0.7	0.57	0.37	0.1
Q63961	C184	reversible	1.62	2.44	3.27	4.1	5.34	6.99
Q68FD5	C1565	so2h	1.15	2.05	2.95	3.85	5.19	6.99
Q6P9Y7	C285	reversible	1.53	2.37	3.21	4.05	5.31	6.99
Q6PDK2	C279	so2h	1	0.86	0.72	0.59	0.38	0.1
Q6PIC6	C449	so2h	0.28	0.26	0.23	0.2	0.16	0.1
Q6PZE0	C254	so3h	1.12	2.03	2.93	3.83	5.19	6.99
Q6PZE0	C795	so2h	0.85	0.74	0.62	0.51	0.34	0.1
Q6Q899	C811	so3h	0.99	0.85	0.72	0.58	0.38	0.1
Q6S5J6	C396	reversible	2.82	3.46	4.1	4.74	5.71	6.99
Q6ZQM0	C287	so3h	0.96	0.83	0.7	0.57	0.37	0.1
Q7TMB8	C1087	so2h	1.32	2.19	3.06	3.94	5.25	6.99
Q7TNC6	C187	reversible	0.89	0.77	0.65	0.53	0.35	0.1
Q7TQJ8	C318	so2h	0.78	0.67	0.57	0.47	0.31	0.1
Q7TQJ8	C321	so2h	0.78	0.67	0.57	0.47	0.31	0.1
Q7TSV6	C130	reversible	1.19	2.08	2.98	3.87	5.21	6.99
Q80T14	C1004	reversible	0.92	0.8	0.67	0.54	0.36	0.1
Q80T79	C2437	so2h	1.53	2.37	3.21	4.05	5.31	6.99
Q80UE6	C409	reversible	1.06	1.97	2.88	3.79	5.16	6.99
Q80WQ8	C841	reversible	1.19	2.08	2.97	3.87	5.21	6.99
Q80WQ9	C308	so3h	1.18	2.08	2.97	3.86	5.2	6.99
Q80Z19	C2385	reversible	0.78	0.68	0.57	0.47	0.31	0.1
Q80Z19	C2622	so3h	1.04	1.96	2.87	3.79	5.16	6.99
Q80ZF8	C482	reversible	1.02	1.94	2.86	3.78	5.15	6.99
Q80ZF8	C492	reversible	1.02	1.94	2.86	3.78	5.15	6.99
Q8BG26	C71	reversible	1.17	2.07	2.96	3.86	5.2	6.99
Q8BGA8	C309	reversible	0.93	0.81	0.68	0.55	0.36	0.1
Q8BGX0	C546	reversible	1.44	2.3	3.15	4	5.28	6.99
Q8BHE8	C43	so3h	1.28	2.16	3.04	3.91	5.23	6.99
Q8BRH4	C393	reversible	1.04	1.96	2.87	3.79	5.16	6.99

Q8BRH4	C434	reversible	0.77	0.67	0.57	0.46	0.31	0.1
Q8BWF0	C422	reversible	0.98	0.85	0.71	0.58	0.37	0.1
Q8BX70	C1092	so3h	3.4	3.95	4.51	5.06	5.89	6.99
Q8C013	C31	reversible	0.84	0.73	0.61	0.5	0.33	0.1
Q8C8B0	C99	reversible	0.9	0.78	0.66	0.53	0.35	0.1
Q8C8R3	C984	so3h	3.27	3.84	4.41	4.99	5.85	6.99
Q8CBY3	C585	so3h	1.18	2.08	2.97	3.86	5.2	6.99
Q8CFI5	C22	so2h	0.81	0.7	0.59	0.49	0.32	0.1
Q8CH72	C62	reversible	0.8	0.69	0.58	0.48	0.32	0.1
Q8CHY6	C419	reversible	1.36	2.23	3.09	3.96	5.26	6.99
Q8CI32	C157	so2h	1.18	2.08	2.97	3.86	5.2	6.99
Q8JZN5	C331	reversible	0.71	0.61	0.52	0.43	0.29	0.1
Q8K411	C619	reversible	0.68	0.59	0.5	0.42	0.28	0.1
Q8VCG4	C96	reversible	0.74	0.64	0.54	0.45	0.3	0.1
Q8VE47	C248	reversible	1.43	2.29	3.14	4	5.28	6.99
Q8VEM9	C297	reversible	0.93	0.8	0.68	0.55	0.36	0.1
Q8VHR5	C421	so2h	1	0.86	0.72	0.59	0.38	0.1
Q91VD9	C179	so3h	1.47	2.32	3.17	4.02	5.29	6.99
Q91XE4	C26	reversible	0.75	0.65	0.55	0.45	0.3	0.1
Q91ZE5	C75	so2h	0.82	0.71	0.6	0.49	0.33	0.1
Q921H8	C381	reversible	1	0.86	0.72	0.59	0.38	0.1
Q921I1	C526	so2h	0.98	0.85	0.71	0.58	0.38	0.1
Q99K43	C184	reversible	0.89	0.77	0.65	0.53	0.35	0.1
Q99N87	C326	reversible	1.11	2.02	2.92	3.83	5.18	6.99
Q99P47	C960	so2h	1.47	2.32	3.17	4.02	5.29	6.99
Q9CQF0	C50	so3h	0.85	0.73	0.62	0.5	0.33	0.1
Q9CQR4	C74	reversible	1.43	2.29	3.14	4	5.28	6.99
Q9CR50	C27	reversible	0.99	0.86	0.72	0.58	0.38	0.1
Q9CR50	C34	reversible	0.99	0.86	0.72	0.58	0.38	0.1
Q9CU65	C675	so3h	0.92	0.79	0.67	0.54	0.36	0.1
Q9CU65	C680	so3h	0.92	0.79	0.67	0.54	0.36	0.1
Q9CXJ4	C444	reversible	1.07	1.98	2.89	3.8	5.17	6.99
Q9CZ30	C187	reversible	0.84	0.72	0.61	0.5	0.33	0.1
Q9D172	C175	reversible	0.97	0.83	0.7	0.57	0.37	0.1
Q9D4J7	C283	so2h	1.04	1.96	2.87	3.79	5.16	6.99
Q9D6I7	C347	reversible	1.03	1.95	2.86	3.78	5.16	6.99
Q9D7U6	C87	so3h	1	1.92	2.85	3.77	5.15	6.99
Q9DBR3	C288	so3h	0.99	0.85	0.72	0.58	0.38	0.1
Q9EPL2	C557	reversible	1.05	1.96	2.88	3.79	5.16	6.99

Q9ESD7	C1253	so3h	0.96	0.83	0.7	0.56	0.37	0.1
Q9ESN2	C49	reversible	0.9	0.78	0.66	0.53	0.35	0.1
Q9JHW2	C170	reversible	0.93	0.8	0.68	0.55	0.36	0.1
Q9QXC1	C10	so2h	1.09	2	2.91	3.81	5.18	6.99
Q9QXZ0	C2880	so3h	0.88	0.76	0.64	0.52	0.34	0.1
Q9QZ59	C100	so3h	0.47	0.41	0.36	0.3	0.22	0.1
Q9QZB9	C125	so2h	0.89	0.77	0.65	0.53	0.35	0.1
Q9QZR9	C1675	reversible	1.18	2.08	2.97	3.86	5.2	6.99
Q9R087	C244	so2h	0.87	0.75	0.63	0.51	0.34	0.1
Q9R0L7	C392	so2h	0.77	0.67	0.57	0.46	0.31	0.1
Q9WUA3	C122	reversible	1.01	1.93	2.85	3.77	5.15	6.99
Q9WVH9	C53	reversible	0.94	0.82	0.69	0.56	0.36	0.1
Q9Z1T6	C1969	so3h	0.92	0.8	0.67	0.54	0.36	0.1
Q9Z2I0	C37	so3h	1.08	1.99	2.9	3.81	5.17	6.99
Q9Z2I0	C551	reversible	1.1	2	2.91	3.82	5.18	6.99
A2RT91	C1309	so2h	1.08	3.21	4.13	6.99	6.99	6.99
B2RXS4	C1141	so2h	0.95	0.64	0.51	0.1	0.1	0.1
B7ZWI3	C44	reversible	1	0.68	0.54	0.1	0.1	0.1
D2J0Y4	C462	so2h	0.88	0.6	0.48	0.1	0.1	0.1
E9PV24	C56	reversible	2.41	4.07	4.78	6.99	6.99	6.99
G3X912	C80	so2h	1.41	3.42	4.29	6.99	6.99	6.99
O08524	C276	so3h	0.92	0.62	0.5	0.1	0.1	0.1
O08550	C1005	so2h	0.92	0.62	0.5	0.1	0.1	0.1
O09118	C341	so3h	1.13	3.25	4.16	6.99	6.99	6.99
O09118	C343	so3h	1.13	3.25	4.16	6.99	6.99	6.99
O09131	C191	reversible	1.11	3.23	4.15	6.99	6.99	6.99
O35566	C185	reversible	0.6	0.42	0.34	0.1	0.1	0.1
O35714	C44	reversible	0.97	0.66	0.52	0.1	0.1	0.1
O35914	C717	so2h	0.82	0.56	0.45	0.1	0.1	0.1
O70481	C511	so2h	0.9	0.61	0.49	0.1	0.1	0.1
O88322	C1317	reversible	0.67	0.47	0.38	0.1	0.1	0.1
O88799	C3349	so3h	1.2	3.29	4.19	6.99	6.99	6.99
O88839	C668	so2h	1.19	3.29	4.19	6.99	6.99	6.99
P02468	C1013	reversible	1.26	3.33	4.22	6.99	6.99	6.99
P02468	C859	so2h	1.02	3.18	4.11	6.99	6.99	6.99
P02468	C954	reversible	1.25	3.32	4.22	6.99	6.99	6.99
P06684	C728	reversible	0.81	0.55	0.44	0.1	0.1	0.1
P06801	C468	reversible	0.73	0.5	0.41	0.1	0.1	0.1
P06909	C920	reversible	1.23	3.31	4.21	6.99	6.99	6.99

P07724	C289	so3h	0.75	0.51	0.41	0.1	0.1	0.1
P10925	C585	so2h	0.59	0.42	0.34	0.1	0.1	0.1
P11103	C125	so2h	1.1	3.23	4.14	6.99	6.99	6.99
P11881	C1653	so3h	2.09	3.86	4.62	6.99	6.99	6.99
P12656	C51	reversible	0.85	0.58	0.46	0.1	0.1	0.1
P17534	C83	so2h	0.91	0.62	0.5	0.1	0.1	0.1
P20444	C143	so2h	1.01	3.17	4.1	6.99	6.99	6.99
P20918	C257	reversible	1.11	3.23	4.15	6.99	6.99	6.99
P20918	C335	reversible	1.49	3.48	4.33	6.99	6.99	6.99
P21981	C230	reversible	1.08	3.22	4.13	6.99	6.99	6.99
P25118	C102	so3h	0.86	0.59	0.47	0.1	0.1	0.1
P28652	C7	so3h	0.9	0.61	0.49	0.1	0.1	0.1
P28825	C686	so3h	0.81	0.55	0.45	0.1	0.1	0.1
P29341	C132	reversible	1.06	3.2	4.13	6.99	6.99	6.99
P32261	C463	reversible	0.86	0.59	0.47	0.1	0.1	0.1
P37889	C754	reversible	1.3	3.35	4.24	6.99	6.99	6.99
P47962	C62	so2h	0.9	0.61	0.49	0.1	0.1	0.1
P48754	C61	so3h	7.81	7.51	7.39	6.99	6.99	6.99
P51410	C134	reversible	1.16	3.26	4.17	6.99	6.99	6.99
P51491	C311	so2h	0.75	0.52	0.42	0.1	0.1	0.1
P55849	C673	so2h	0.88	0.6	0.48	0.1	0.1	0.1
P59511	C1495	so3h	0.96	0.65	0.52	0.1	0.1	0.1
P59644	C556	so2h	1.43	3.44	4.3	6.99	6.99	6.99
P59997	C910	so2h	0.81	0.55	0.44	0.1	0.1	0.1
P59997	C912	so3h	0.81	0.55	0.44	0.1	0.1	0.1
P60882	C2205	so2h	0.84	0.57	0.46	0.1	0.1	0.1
P63260	C217	reversible	1.49	3.48	4.33	6.99	6.99	6.99
P70312	C102	reversible	0.74	0.51	0.41	0.1	0.1	0.1
P70414	C762	reversible	0.88	0.6	0.48	0.1	0.1	0.1
P97314	C143	so3h	1.07	3.21	4.13	6.99	6.99	6.99
P97315	C146	so3h	0.66	0.46	0.37	0.1	0.1	0.1
P98156	C344	reversible	0.73	0.5	0.41	0.1	0.1	0.1
Q00724	C178	reversible	1.66	3.59	4.42	6.99	6.99	6.99
Q01721	C175	reversible	0.66	0.46	0.37	0.1	0.1	0.1
Q05793	C2456	so3h	2.99	4.43	5.06	6.99	6.99	6.99
Q07802	C151	reversible	1.37	3.4	4.27	6.99	6.99	6.99
Q2VLH6	C576	reversible	1.3	3.36	4.24	6.99	6.99	6.99
Q3TZX3	C173	so2h	0.91	0.62	0.49	0.1	0.1	0.1
Q3UDW8	C532	so2h	1.02	3.18	4.1	6.99	6.99	6.99

Q3UHK3	C1058	reversible	0.95	0.65	0.52	0.1	0.1	0.1
Q3ZRW6	C116	reversible	1.06	3.2	4.12	6.99	6.99	6.99
Q497K7	C197	so3h	0.96	0.65	0.52	0.1	0.1	0.1
Q5DU56	C325	reversible	0.82	0.56	0.45	0.1	0.1	0.1
Q5NC05	C903	so2h	1.08	3.22	4.14	6.99	6.99	6.99
Q5SPW0	C138	so2h	1.36	3.39	4.27	6.99	6.99	6.99
Q5XJY6	C515	so2h	0.63	0.44	0.36	0.1	0.1	0.1
Q5XJY6	C70	so3h	1.43	3.44	4.3	6.99	6.99	6.99
Q5XJY6	C71	so3h	1.43	3.44	4.3	6.99	6.99	6.99
Q60585	C317	reversible	0.98	0.66	0.53	0.1	0.1	0.1
Q60676	C404	reversible	1.63	3.57	4.4	6.99	6.99	6.99
Q60718	C636	reversible	0.83	0.57	0.45	0.1	0.1	0.1
Q60766	C375	so2h	0.87	0.59	0.47	0.1	0.1	0.1
Q60864	C278	reversible	0.69	0.48	0.39	0.1	0.1	0.1
Q61543	C980	reversible	1.14	3.25	4.16	6.99	6.99	6.99
Q61554	C1008	reversible	1.29	3.35	4.23	6.99	6.99	6.99
Q61554	C1032	so3h	1.59	3.54	4.38	6.99	6.99	6.99
Q61554	C1039	so3h	1.59	3.54	4.38	6.99	6.99	6.99
Q61554	C2061	reversible	0.88	0.6	0.48	0.1	0.1	0.1
Q61555	C2335	so3h	0.87	0.6	0.48	0.1	0.1	0.1
Q62311	C231	so3h	0.89	0.61	0.49	0.1	0.1	0.1
Q64737	C134	reversible	0.96	0.65	0.52	0.1	0.1	0.1
Q67BT3	C291	reversible	1.39	3.41	4.28	6.99	6.99	6.99
Q68FE8	C499	so3h	1.13	3.25	4.16	6.99	6.99	6.99
Q6A0A9	C147	so2h	0.61	0.43	0.35	0.1	0.1	0.1
Q6A0D4	C192	reversible	0.97	0.66	0.52	0.1	0.1	0.1
Q6A4J8	C489	so2h	0.85	0.58	0.47	0.1	0.1	0.1
Q6DIB5	C167	so2h	0.83	0.57	0.45	0.1	0.1	0.1
Q6NS46	C505	so3h	0.9	0.61	0.49	0.1	0.1	0.1
Q6PB97	C33	reversible	1.16	3.27	4.18	6.99	6.99	6.99
Q6PDN3	C486	so2h	0.2	0.16	0.15	0.1	0.1	0.1
Q6PZE0	C7340	reversible	1.15	3.26	4.17	6.99	6.99	6.99
Q6Q899	C814	so3h	0.99	0.67	0.53	0.1	0.1	0.1
Q6Q899	C819	so3h	0.99	0.67	0.53	0.1	0.1	0.1
Q6V595	C337	so2h	1.18	3.28	4.18	6.99	6.99	6.99
Q6X6Z7	C76	reversible	1.12	3.24	4.15	6.99	6.99	6.99
Q6ZWR4	C250	so3h	0.85	0.58	0.46	0.1	0.1	0.1
Q769J6	C700	so2h	0.95	0.64	0.51	0.1	0.1	0.1
Q7TQ48	C648	reversible	0.99	0.67	0.53	0.1	0.1	0.1

Q80WQ9	C305	so3h	1.18	3.28	4.18	6.99	6.99	6.99
Q80Y50	C27	so2h	0.72	0.5	0.4	0.1	0.1	0.1
Q80Y50	C31	so2h	0.72	0.5	0.4	0.1	0.1	0.1
Q80Z19	C2619	so3h	1.04	3.19	4.11	6.99	6.99	6.99
Q8BFY9	C862	reversible	1.06	3.2	4.12	6.99	6.99	6.99
Q8BGS3	C383	reversible	1.12	3.24	4.15	6.99	6.99	6.99
Q8BKV0	C90	so2h	1.03	3.18	4.11	6.99	6.99	6.99
Q8BMF3	C275	reversible	0.91	0.62	0.49	0.1	0.1	0.1
Q8BPM0	C160	so2h	1	0.67	0.54	0.1	0.1	0.1
Q8BQZ5	C116	so2h	1.26	3.33	4.22	6.99	6.99	6.99
Q8BU30	C27	so3h	0.82	0.56	0.45	0.1	0.1	0.1
Q8BX09	C212	so2h	0.85	0.58	0.47	0.1	0.1	0.1
Q8BX46	C210	so2h	0.4	0.29	0.25	0.1	0.1	0.1
Q8BX70	C1096	so2h	3.4	4.7	5.26	6.99	6.99	6.99
Q8C0S4	C461	reversible	1	0.68	0.54	0.1	0.1	0.1
Q8C5V5	C488	so2h	1.4	3.42	4.29	6.99	6.99	6.99
Q8C7R4	C721	reversible	1	3.16	4.1	6.99	6.99	6.99
Q8CH72	C65	reversible	0.8	0.55	0.44	0.1	0.1	0.1
Q8CHY6	C425	reversible	1.4	3.42	4.29	6.99	6.99	6.99
Q8CI94	C143	reversible	0.71	0.49	0.4	0.1	0.1	0.1
Q8CJ27	C882	so3h	1.22	3.3	4.2	6.99	6.99	6.99
Q8CJ69	C105	reversible	1.01	3.17	4.1	6.99	6.99	6.99
Q8JZZ6	C153	reversible	0.32	0.24	0.21	0.1	0.1	0.1
Q8K0E8	C213	so2h	1.29	3.35	4.24	6.99	6.99	6.99
Q8K1R3	C4	so2h	1.19	3.28	4.19	6.99	6.99	6.99
Q8QZZ7	C167	reversible	1.11	3.23	4.15	6.99	6.99	6.99
Q8R1G2	C198	so3h	7.86	7.54	7.41	6.99	6.99	6.99
Q8R2Q4	C329	reversible	1.36	3.39	4.27	6.99	6.99	6.99
Q8VCM7	C164	reversible	1.87	3.72	4.51	6.99	6.99	6.99
Q8VDI7	C43	so3h	1.01	3.17	4.1	6.99	6.99	6.99
Q8VE19	C775	so2h	0.73	0.5	0.41	0.1	0.1	0.1
Q8VEE1	C31	so2h	0.96	0.65	0.52	0.1	0.1	0.1
Q8VHR5	C410	so2h	1	0.68	0.54	0.1	0.1	0.1
Q91VD9	C176	so2h	1.47	3.46	4.32	6.99	6.99	6.99
Q91VD9	C182	so3h	1.47	3.46	4.32	6.99	6.99	6.99
Q91WG7	C396	reversible	0.6	0.42	0.34	0.1	0.1	0.1
Q91YP0	C273	reversible	0.94	0.64	0.51	0.1	0.1	0.1
Q91ZX7	C3342	so3h	0.6	0.42	0.35	0.1	0.1	0.1
Q924D0	C130	so3h	0.84	0.57	0.46	0.1	0.1	0.1

Q99J39	C447	reversible	1.13	3.25	4.16	6.99	6.99	6.99
Q99KE8	C336	reversible	1	0.68	0.54	0.1	0.1	0.1
Q99KI0	C410	reversible	0.52	0.37	0.3	0.1	0.1	0.1
Q99LD4	C51	so2h	1.08	3.22	4.14	6.99	6.99	6.99
Q99N48	C103	reversible	0.66	0.46	0.37	0.1	0.1	0.1
Q99NB9	C965	so3h	1.35	3.39	4.27	6.99	6.99	6.99
Q99P47	C964	so3h	1.47	3.47	4.32	6.99	6.99	6.99
Q99PQ2	C114	reversible	1.48	3.47	4.33	6.99	6.99	6.99
Q9CPY7	C445	reversible	0.82	0.56	0.45	0.1	0.1	0.1
Q9CQ29	C56	reversible	1.12	3.24	4.16	6.99	6.99	6.99
Q9CQ29	C57	reversible	1.12	3.24	4.16	6.99	6.99	6.99
Q9CQ65	C163	so3h	0.7	0.49	0.39	0.1	0.1	0.1
Q9CU65	C683	so2h	0.92	0.63	0.5	0.1	0.1	0.1
Q9CUU3	C674	reversible	1.04	3.19	4.11	6.99	6.99	6.99
Q9CXE0	C432	reversible	0.84	0.57	0.46	0.1	0.1	0.1
Q9CYQ7	C6	reversible	1.13	3.25	4.16	6.99	6.99	6.99
Q9CZU6	C359	so2h	1.17	3.27	4.18	6.99	6.99	6.99
Q9D0K2	C235	so3h	0.93	0.63	0.5	0.1	0.1	0.1
Q9D2G2	C105	reversible	1.28	3.35	4.23	6.99	6.99	6.99
Q9D2H6	C504	reversible	0.83	0.57	0.45	0.1	0.1	0.1
Q9D6E4	C219	reversible	1.32	3.36	4.25	6.99	6.99	6.99
Q9D6F9	C239	reversible	1	0.67	0.54	0.1	0.1	0.1
Q9D8W7	C134	so2h	1.22	3.3	4.2	6.99	6.99	6.99
Q9DB26	C49	reversible	1.26	3.33	4.22	6.99	6.99	6.99
Q9DBJ1	C153	reversible	1.07	3.21	4.13	6.99	6.99	6.99
Q9EQJ0	C207	so2h	1.04	3.19	4.12	6.99	6.99	6.99
Q9ESD7	C1255	so3h	0.96	0.65	0.52	0.1	0.1	0.1
Q9JIP3	C87	reversible	0.92	0.63	0.5	0.1	0.1	0.1
Q9JJB1	C208	so3h	1.01	3.17	4.1	6.99	6.99	6.99
Q9QUH0	C23	so2h	1.09	3.22	4.14	6.99	6.99	6.99
Q9QUH0	C26	so2h	1.09	3.22	4.14	6.99	6.99	6.99
Q9QXC1	C15	so2h	0.98	0.67	0.53	0.1	0.1	0.1
Q9QXK2	C207	so3h	0.63	0.44	0.36	0.1	0.1	0.1
Q9QZ59	C103	so3h	0.47	0.34	0.28	0.1	0.1	0.1
Q9QZB9	C126	so2h	0.89	0.61	0.48	0.1	0.1	0.1
Q9QZF2	C12	so3h	0.61	0.43	0.35	0.1	0.1	0.1
Q9QZF2	C20	so2h	0.61	0.43	0.35	0.1	0.1	0.1
Q9R016	C1170	reversible	0.77	0.53	0.43	0.1	0.1	0.1
Q9R087	C270	so3h	0.63	0.44	0.36	0.1	0.1	0.1

Q9R1A3	C374	so2h	1.1	3.23	4.15	6.99	6.99	6.99
Q9R1A3	C376	so3h	1.1	3.23	4.15	6.99	6.99	6.99
Q9R1B9	C1478	so2h	0.88	0.6	0.48	0.1	0.1	0.1
Q9WUB0	C471	so2h	0.96	0.65	0.52	0.1	0.1	0.1
Q9WUU9	C1260	reversible	0.72	0.5	0.4	0.1	0.1	0.1
Q9WVQ5	C31	reversible	0.94	0.64	0.51	0.1	0.1	0.1
Q9Z0E2	C111	reversible	0.57	0.4	0.33	0.1	0.1	0.1
Q9Z0F8	C635	reversible	1.23	3.31	4.21	6.99	6.99	6.99
Q9Z2E2	C368	reversible	1.07	3.21	4.13	6.99	6.99	6.99
Q9Z2E2	C374	reversible	0.91	0.62	0.49	0.1	0.1	0.1
A2ARA8	C21	so3h	0.1	0.21	0.31	0.41	0.56	0.76
A2ARA8	C22	so3h	0.1	0.21	0.31	0.41	0.56	0.76
A2ASS6	C6705	so2h	0.1	0.22	0.34	0.46	0.63	0.87
B1AY10	C720	reversible	6.99	6.11	5.23	4.34	3.02	1.26
D3Z423	C30	reversible	0.1	0.22	0.33	0.45	0.62	0.84
O08529	C39	reversible	6.99	6.1	5.2	4.31	2.96	1.17
O08709	C47	so2h	0.1	0.11	0.11	0.12	0.12	0.13
O35118	C236	so2h	6.99	6.22	5.45	4.68	3.52	1.98
O88516	C424	reversible	6.99	6.09	5.19	4.29	2.93	1.13
P06151	C163	so3h	6.99	6.19	5.39	4.59	3.38	1.78
P07724	C385	so3h	6.99	6.33	5.67	5.01	4.02	2.7
P08249	C93	so3h	6.99	6.14	5.29	4.44	3.16	1.46
P10493	C712	reversible	6.99	6.09	5.19	4.3	2.95	1.15
P14152	C137	so3h	6.99	6.11	5.23	4.35	3.04	1.28
P26262	C383	reversible	6.99	6.08	5.18	4.27	2.91	1.1
P33434	C21	so3h	6.99	6.13	5.28	4.42	3.14	1.42
P33434	C22	so3h	6.99	6.13	5.28	4.42	3.14	1.42
P34960	C3	reversible	6.99	6.18	5.38	4.57	3.36	1.75
P35235	C104	reversible	6.99	6.08	5.17	4.27	2.9	1.09
P42227	C251	so2h	0.1	0.24	0.37	0.51	0.71	0.98
P46660	C182	reversible	6.99	6.21	5.43	4.65	3.48	1.92
P47226	C414	so3h	6.99	6.18	5.37	4.56	3.34	1.72
P53702	C182	reversible	6.99	6.13	5.26	4.39	3.1	1.37
P54071	C336	reversible	0.1	0.22	0.33	0.44	0.61	0.83
P56400	C26	reversible	0.1	0.22	0.34	0.46	0.64	0.88
P60867	C36	reversible	6.99	6.12	5.26	4.39	3.09	1.36
P62259	C97	so2h	6.99	6.12	5.24	4.37	3.05	1.3
Q05920	C372	reversible	0.1	0.1	0.09	0.09	0.08	0.07
Q07417	C109	reversible	0.1	0.2	0.29	0.38	0.51	0.69

Q0KK56	C573	so2h	6.99	6.08	5.16	4.25	2.88	1.05
Q2LKU9	C334	so3h	6.99	6.08	5.18	4.27	2.91	1.09
Q2VLH6	C139	so2h	6.99	6.08	5.16	4.25	2.88	1.05
Q3TWL2	C140	so3h	6.99	6.23	5.46	4.7	3.55	2.02
Q3TXX3	C381	reversible	0.1	0.22	0.34	0.46	0.64	0.88
Q3TXX3	C382	reversible	0.1	0.22	0.34	0.46	0.64	0.88
Q3UV16	C300	reversible	6.99	6.13	5.27	4.41	3.12	1.4
Q3UXZ9	C1166	so3h	0.1	0.23	0.35	0.47	0.65	0.9
Q3UXZ9	C1177	so3h	6.99	6.1	5.21	4.32	2.98	1.2
Q3V0C1	C294	so2h	6.99	6.17	5.35	4.53	3.3	1.66
Q3V384	C100	reversible	6.99	6.07	5.16	4.24	2.86	1.03
Q3V3R4	C548	so2h	0.1	0.19	0.27	0.36	0.48	0.65
Q4VC17	C1158	so2h	6.99	6.08	5.18	4.27	2.91	1.1
Q5NC05	C902	so2h	0.1	0.23	0.36	0.49	0.69	0.94
Q5U405	C290	so2h	6.99	6.43	5.87	5.31	4.47	3.35
Q61554	C1081	reversible	6.99	6.09	5.19	4.3	2.95	1.15
Q62178	C497	so2h	0.1	0.21	0.31	0.41	0.56	0.76
Q62312	C79	reversible	6.99	6.13	5.27	4.42	3.13	1.41
Q64518	C349	reversible	0.1	0.24	0.38	0.51	0.71	0.98
Q64518	C447	reversible	6.99	6.12	5.25	4.38	3.08	1.34
Q64518	C636	reversible	6.99	6.08	5.18	4.27	2.91	1.1
Q64527	C336	so3h	6.99	6.1	5.2	4.31	2.96	1.17
Q69Z69	C760	reversible	6.99	6.09	5.18	4.28	2.92	1.12
Q69ZK6	C1679	so2h	0.1	0.14	0.18	0.22	0.28	0.35
Q6NS59	C1429	reversible	0.1	0.23	0.35	0.48	0.66	0.91
Q6P1B1	C316	so3h	6.99	6.07	5.15	4.23	2.85	1.01
Q6P1G2	C1070	so2h	6.99	6.08	5.18	4.27	2.91	1.1
Q6P1G2	C589	so3h	6.99	6.14	5.29	4.45	3.17	1.48
Q6R6I7	C40	so2h	6.99	6.46	5.93	5.4	4.6	3.54
Q6R6I7	C47	so2h	6.99	6.46	5.93	5.4	4.6	3.54
Q6ZWV3	C80	so2h	6.99	6.08	5.18	4.27	2.91	1.09
Q7TNG5	C301	reversible	0.1	0.24	0.37	0.5	0.69	0.96
Q7TNM2	C249	so3h	6.99	6.23	5.46	4.69	3.55	2.02
Q7TPQ9	C183	so3h	6.99	6.25	5.52	4.78	3.67	2.19
Q80SU3	C341	reversible	6.99	6.11	5.23	4.35	3.03	1.27
Q80UG2	C137	reversible	6.99	6.09	5.18	4.28	2.93	1.12
Q80UG2	C813	reversible	0.1	0.23	0.36	0.48	0.67	0.93
Q80UJ1	C435	so2h	0.1	0.24	0.38	0.51	0.72	0.99
Q80UL9	C45	so2h	0.1	0.24	0.37	0.5	0.7	0.96

Q80UW0	C258	reversible	0.1	0.23	0.35	0.47	0.66	0.9
Q8BIV3	C690	so2h	0.1	0.24	0.38	0.52	0.72	1
Q8BKX6	C1021	reversible	0.1	0.13	0.16	0.18	0.22	0.27
Q8BMF3	C82	reversible	0.1	0.19	0.28	0.36	0.49	0.67
Q8BWQ6	C303	so3h	6.99	6.08	5.16	4.25	2.88	1.06
Q8BWQ6	C315	so3h	6.99	6.08	5.16	4.25	2.88	1.06
Q8BWR2	C42	reversible	6.99	6.09	5.19	4.29	2.94	1.15
Q8BYL4	C408	reversible	6.99	6.08	5.16	4.24	2.87	1.04
Q8CBA2	C353	reversible	6.99	6.14	5.28	4.43	3.15	1.44
Q8CD54	C1703	reversible	6.99	6.15	5.3	4.45	3.18	1.49
Q8CFG4	C110	so3h	6.99	6.16	5.32	4.48	3.23	1.56
Q8CGY6	C784	reversible	6.99	6.08	5.16	4.25	2.87	1.04
Q8K0C4	C345	so2h	6.99	6.1	5.2	4.31	2.96	1.17
Q8K1J6	C21	so3h	0.1	0.23	0.35	0.47	0.65	0.89
Q8K224	C486	reversible	0.1	0.22	0.34	0.45	0.63	0.86
Q8R0W0	C2412	reversible	0.1	0.22	0.34	0.46	0.64	0.88
Q91VW5	C1203	so2h	6.99	6.15	5.31	4.46	3.2	1.51
Q91Z83	C947	reversible	0.1	0.18	0.26	0.34	0.46	0.62
Q91ZJ5	C276	so3h	6.99	6.09	5.18	4.28	2.93	1.12
Q91ZT9	C237	reversible	0.1	0.23	0.35	0.47	0.66	0.9
Q91ZX7	C4340	so2h	6.99	6.08	5.16	4.24	2.87	1.04
Q921F4	C284	reversible	0.1	0.23	0.36	0.48	0.67	0.93
Q923J1	C36	so3h	6.99	6.16	5.34	4.51	3.27	1.61
Q924C1	C44	so2h	6.99	6.15	5.31	4.47	3.2	1.52
Q99J62	C194	so3h	6.99	6.08	5.16	4.25	2.87	1.04
Q99KE1	C190	so3h	6.99	6.09	5.19	4.29	2.94	1.14
Q99KI0	C126	so3h	6.99	6.09	5.19	4.29	2.94	1.14
Q99KI0	C385	so3h	6.99	6.12	5.25	4.38	3.08	1.34
Q99N64	C183	so3h	0.1	0.19	0.28	0.37	0.5	0.68
Q9CR25	C47	so3h	6.99	6.11	5.23	4.35	3.03	1.27
Q9CU24	C148	so2h	0.1	0.21	0.31	0.41	0.56	0.76
Q9CU65	C726	so3h	0.1	0.19	0.28	0.37	0.51	0.69
Q9CZ13	C410	so3h	6.99	6.11	5.22	4.34	3.01	1.24
Q9D0E1	C708	so3h	6.99	6.09	5.19	4.28	2.93	1.12
Q9D0M3	C139	reversible	6.99	6.21	5.42	4.63	3.46	1.88
Q9D2H5	C8	reversible	6.99	6.08	5.17	4.26	2.89	1.07
Q9D8M7	C432	reversible	0.1	0.24	0.38	0.51	0.71	0.98
Q9DA16	C151	so3h	0.1	0.18	0.25	0.33	0.44	0.59
Q9DB77	C192	so3h	6.99	6.09	5.19	4.29	2.94	1.14

Q9EQW7	C1430	so3h	0.1	0.14	0.18	0.21	0.27	0.34
Q9ER04	C206	so2h	6.99	6.3	5.61	4.92	3.89	2.51
Q9ER04	C209	so2h	6.99	6.3	5.61	4.92	3.89	2.51
Q9ERU9	C1366	reversible	0.1	0.23	0.36	0.49	0.68	0.94
Q9JLV5	C462	so3h	0.1	0.2	0.29	0.39	0.53	0.72
Q9QWK5	C1164	so3h	0.1	0.2	0.3	0.4	0.54	0.74
Q9QZC7	C62	reversible	0.1	0.2	0.3	0.4	0.55	0.75
Q9WTL4	C280	reversible	6.99	6.1	5.21	4.33	2.99	1.22
Q9WUA3	C717	so3h	6.99	6.12	5.25	4.38	3.07	1.33
Q9WUB0	C211	reversible	0.1	0.22	0.34	0.46	0.64	0.88
Q9Z2U2	C1931	so3h	6.99	6.11	5.23	4.35	3.03	1.26
A2AGL3	C2226	reversible	6.99	6.99	6.99	4.89	3.4	1.06
A2AVA0	C1595	reversible	0.1	0.1	0.1	0.3	0.44	0.66
A2RSQ0	C193	so2h	0.1	0.1	0.1	0.38	0.58	0.89
O08709	C47	so3h	0.1	0.1	0.1	0.4	0.6	0.93
O35118	C245	so2h	6.99	6.99	6.99	5.22	3.96	1.98
O35453	C400	so3h	0.1	0.1	0.1	0.39	0.59	0.91
O54775	C153	reversible	0.1	0.1	0.1	0.39	0.59	0.91
O88799	C3120	so2h	0.1	0.1	0.1	0.31	0.45	0.67
O88799	C3221	so3h	0.1	0.1	0.1	0.19	0.26	0.36
P00342	C163	so3h	6.99	6.99	6.99	4.97	3.54	1.28
P00520	C5	so3h	6.99	6.99	6.99	5.01	3.61	1.4
P05125	C145	reversible	0.1	0.1	0.1	0.37	0.56	0.86
P08249	C89	so2h	6.99	6.99	6.99	5.03	3.64	1.46
P0CW02	C78	so3h	0.1	0.1	0.1	0.3	0.45	0.67
P15265	C40	reversible	6.99	6.99	6.99	5.35	4.19	2.36
P19221	C215	reversible	6.99	6.99	6.99	4.97	3.53	1.28
P21278	C330	reversible	0.1	0.1	0.1	0.38	0.58	0.89
P26039	C1661	so2h	6.99	6.99	6.99	5.03	3.65	1.47
P26039	C1671	so3h	6.99	6.99	6.99	5.03	3.65	1.47
P26043	C117	reversible	6.99	6.99	6.99	4.9	3.43	1.1
P29268	C241	so2h	0.1	0.1	0.1	0.37	0.55	0.84
P30416	C103	so2h	6.99	6.99	6.99	4.88	3.38	1.03
P35385	C125	so2h	0.1	0.1	0.1	0.32	0.47	0.71
P45376	C299	so3h	6.99	6.99	6.99	4.94	3.5	1.22
P47226	C418	so3h	6.99	6.99	6.99	5.12	3.8	1.72
P48754	C44	reversible	6.99	6.99	6.99	5.57	4.57	2.99
P50543	C8	so3h	0.1	0.1	0.1	0.35	0.53	0.8
P52503	C79	so3h	6.99	6.99	6.99	4.93	3.48	1.18

P55098	C138	so2h	0.1	0.1	0.1	0.36	0.53	0.81
P59222	C278	reversible	6.99	6.99	6.99	4.91	3.43	1.11
P62259	C98	so2h	6.99	6.99	6.99	4.98	3.55	1.3
Q01065	C409	reversible	0.1	0.1	0.1	0.3	0.44	0.66
Q04592	C1599	reversible	6.99	6.99	6.99	4.97	3.54	1.29
Q08376	C366	reversible	6.99	6.99	6.99	5.03	3.65	1.47
Q11131	C365	reversible	6.99	6.99	6.99	4.87	3.37	1.01
Q3U128	C52	so3h	0.1	0.1	0.1	0.35	0.53	0.81
Q45VK7	C3224	so3h	6.99	6.99	6.99	4.87	3.38	1.02
Q4U2R1	C3981	so3h	0.1	0.1	0.1	0.38	0.57	0.88
Q52KR2	C48	reversible	0.1	0.1	0.1	0.36	0.55	0.83
Q5HZK1	C115	reversible	0.1	0.1	0.1	0.41	0.63	0.98
Q5SSH7	C2826	so2h	6.99	6.99	6.99	4.97	3.55	1.3
Q5U405	C293	so3h	6.99	6.99	6.99	5.7	4.79	3.35
Q61598	C317	reversible	6.99	6.99	6.99	5.13	3.82	1.75
Q62028	C1376	so2h	6.99	6.99	6.99	4.91	3.43	1.11
Q63836	C31	reversible	0.1	0.1	0.1	0.41	0.63	0.97
Q63836	C371	reversible	6.99	6.99	6.99	4.96	3.53	1.27
Q68FD5	C736	reversible	6.99	6.99	6.99	4.91	3.44	1.12
Q69ZB0	C85	reversible	0.1	0.1	0.1	0.37	0.56	0.86
Q69ZK6	C1682	so3h	0.1	0.1	0.1	0.19	0.26	0.35
Q69ZX8	C105	reversible	6.99	6.99	6.99	4.99	3.58	1.35
Q6P8X6	C193	reversible	6.99	6.99	6.99	5.09	3.74	1.63
Q6P8X6	C196	reversible	6.99	6.99	6.99	5.09	3.74	1.63
Q6R6I7	C380	so2h	0.1	0.1	0.1	0.38	0.58	0.89
Q6WKZ8	C513	reversible	0.1	0.1	0.1	0.42	0.64	0.99
Q6ZQM0	C286	reversible	0.1	0.1	0.1	0.37	0.56	0.86
Q71FD7	C268	so2h	0.1	0.1	0.1	0.33	0.48	0.73
Q7TNM2	C246	so3h	6.99	6.99	6.99	5.23	3.98	2.02
Q7TPQ9	C182	so3h	6.99	6.99	6.99	5.29	4.09	2.19
Q7TS63	C827	reversible	0.1	0.1	0.1	0.41	0.62	0.96
Q80SU3	C102	reversible	6.99	6.99	6.99	5.05	3.68	1.51
Q80TA9	C430	reversible	0.1	0.1	0.1	0.39	0.59	0.91
Q80UG2	C803	reversible	0.1	0.1	0.1	0.4	0.6	0.93
Q80UW0	C256	reversible	6.99	6.99	6.99	4.94	3.49	1.21
Q80Y84	C708	reversible	6.99	6.99	6.99	5.11	3.79	1.69
Q80Z71	C1486	reversible	6.99	6.99	6.99	4.91	3.44	1.12
Q810J8	C103	reversible	6.99	6.99	6.99	4.9	3.42	1.1
Q811B3	C1396	reversible	0.1	0.1	0.1	0.42	0.64	1

Q811C2	C388	reversible	0.1	0.1	0.1	0.4	0.61	0.94
Q811T9	C668	reversible	0.1	0.1	0.1	0.31	0.46	0.69
Q8BGC4	C55	reversible	0.1	0.1	0.1	0.4	0.61	0.93
Q8BIK4	C1488	reversible	6.99	6.99	6.99	4.87	3.37	1.01
Q8BKC5	C682	so3h	6.99	6.99	6.99	5.32	4.14	2.28
Q8BKX6	C3184	reversible	0.1	0.1	0.1	0.34	0.5	0.76
Q8BMQ3	C863	reversible	0.1	0.1	0.1	0.25	0.35	0.51
Q8BRH4	C484	reversible	0.1	0.1	0.1	0.3	0.45	0.67
Q8BWT1	C382	reversible	0.1	0.1	0.1	0.35	0.52	0.78
Q8C0R9	C524	so3h	6.99	6.99	6.99	4.87	3.37	1.01
Q8C267	C359	reversible	6.99	6.99	6.99	4.89	3.4	1.05
Q8C6B2	C62	reversible	0.1	0.1	0.1	0.32	0.47	0.71
Q8CBX0	C300	reversible	0.1	0.1	0.1	0.25	0.35	0.5
Q8CFG4	C112	so3h	6.99	6.99	6.99	5.07	3.7	1.56
Q8CG65	C3182	so2h	6.99	6.99	6.99	5.02	3.63	1.43
Q8K354	C226	so2h	6.99	6.99	6.99	4.89	3.4	1.05
Q8K354	C227	so2h	6.99	6.99	6.99	4.89	3.4	1.05
Q8R4U0	C207	so2h	0.1	0.1	0.1	0.32	0.47	0.71
Q8R4U0	C212	so2h	0.1	0.1	0.1	0.32	0.47	0.71
Q91V24	C772	reversible	6.99	6.99	6.99	5.06	3.7	1.54
Q91VR2	C103	so3h	0.1	0.1	0.1	0.34	0.5	0.76
Q91VY5	C840	so2h	0.1	0.1	0.1	0.39	0.6	0.92
Q91WS0	C83	so3h	6.99	6.99	6.99	4.92	3.46	1.16
Q91WT8	C145	so2h	0.1	0.1	0.1	0.4	0.6	0.93
Q91WT8	C151	so3h	0.1	0.1	0.1	0.4	0.6	0.93
Q91XD7	C98	reversible	6.99	6.99	6.99	4.88	3.39	1.04
Q91YT0	C206	so3h	6.99	6.99	6.99	5.24	4.01	2.06
Q91YT0	C255	so3h	0.1	0.1	0.1	0.36	0.55	0.83
Q91ZX7	C4157	so2h	0.1	0.1	0.1	0.35	0.52	0.8
Q921G7	C560	so3h	0.1	0.1	0.1	0.42	0.64	0.98
Q924C1	C50	so2h	6.99	6.99	6.99	5.05	3.68	1.52
Q99J62	C180	so2h	6.99	6.99	6.99	4.94	3.49	1.21
Q99N87	C318	so3h	0.1	0.1	0.1	0.24	0.33	0.48
Q9CU24	C404	so3h	6.99	6.99	6.99	4.88	3.38	1.02
Q9CU65	C729	so3h	0.1	0.1	0.1	0.31	0.46	0.69
Q9CWD0	C125	so2h	0.1	0.1	0.1	0.31	0.46	0.69
Q9CWD0	C48	so3h	6.99	6.99	6.99	5.09	3.75	1.64
Q9CZ13	C453	so3h	6.99	6.99	6.99	4.92	3.45	1.13
Q9CZ96	C110	so2h	6.99	6.99	6.99	5.25	4.02	2.09

Q9D0B5	C36	so3h	0.1	0.1	0.1	0.31	0.45	0.68
Q9D0M0	C208	so3h	0.1	0.1	0.1	0.38	0.58	0.89
Q9DAG9	C276	reversible	0.1	0.1	0.1	0.22	0.3	0.43
Q9DB20	C141	so2h	0.1	0.1	0.1	0.42	0.64	1
Q9DBR3	C3	reversible	6.99	6.99	6.99	4.93	3.47	1.17
Q9ERR8	C105	so2h	0.1	0.1	0.1	0.11	0.11	0.11
Q9ERR8	C108	so2h	0.1	0.1	0.1	0.11	0.11	0.11
Q9JLV6	C444	so2h	6.99	6.99	6.99	4.9	3.43	1.1
Q9R160	C620	so2h	0.1	0.1	0.1	0.33	0.49	0.74
Q9WV74	C75	reversible	6.99	6.99	6.99	6.3	5.82	5.06
Q9Z0J1	C682	so2h	6.99	6.99	6.99	4.87	3.37	1
Q9Z0J1	C688	so3h	6.99	6.99	6.99	4.87	3.37	1
Q9Z1K6	C160	so2h	6.99	6.99	6.99	4.93	3.48	1.18

Supplemental Table S2. Essential 10 biological functions associated with signature cysteine O-PTMs in each temporal cluster**.**

Biological Function #	Biological Function	Cluster	UniProt ID	Modified Amino Acid	Cysteine PTM type
BF1	Neutrophil degranulation	1	Q61598	C302	reversible
BF1	Neutrophil degranulation	2	Q01853	C209	reversible
BF1	Neutrophil degranulation	2	Q01853	C691	reversible
BF1	Neutrophil degranulation	3	Q8BY89	C401	reversible
BF1	Neutrophil degranulation	4	Q61598	C414	reversible
BF1	Neutrophil degranulation	5	P10605	C319	reversible
BF1	Neutrophil degranulation	1	P09528	C131	reversible
BF1	Neutrophil degranulation	2	Q61598	C282	reversible
BF1	Neutrophil degranulation	2	Q61598	C317	reversible
BF1	Neutrophil degranulation	3	Q08481	C470	reversible
BF1	Neutrophil degranulation	4	Q60854	C102	reversible
BF1	Neutrophil degranulation	5	Q60854	C352	reversible
BF1	Neutrophil degranulation	1	Q9D1Q6	C189	reversible
BF1	Neutrophil degranulation	1	Q9D1Q6	C301	reversible
BF1	Neutrophil degranulation	1	Q9D1Q6	C318	reversible
BF1	Neutrophil degranulation	2	P80314	C395	reversible
BF1	Neutrophil degranulation	3	P58252	C567	reversible
BF1	Neutrophil degranulation	4	Q8CI94	C373	reversible
BF1	Neutrophil degranulation	4	Q8CI94	C581	reversible
BF1	Neutrophil degranulation	5	O08807	C54	reversible
BF1	Neutrophil degranulation	1	P06745	C404	reversible
BF1	Neutrophil degranulation	2	P58252	C290	reversible
BF1	Neutrophil degranulation	2	P58252	C591	reversible
BF1	Neutrophil degranulation	2	P58252	C693	reversible
BF1	Neutrophil degranulation	3	P24270	C232	reversible
BF1	Neutrophil degranulation	3	P24270	C425	reversible
BF1	Neutrophil degranulation	4	P18242	C288	reversible
BF1	Neutrophil degranulation	5	P10126	C234	reversible
BF1	Neutrophil degranulation	1	O08709	C47	reversible
BF1	Neutrophil degranulation	1	O08709	C47	so2h
BF1	Neutrophil degranulation	1	O08709	C47	so3h
BF1	Neutrophil degranulation	2	Q921H8	C381	reversible
BF1	Neutrophil degranulation	3	Q8CI94	C437	reversible
BF1	Neutrophil degranulation	3	Q8CI94	C143	reversible

BF1	Neutrophil degranulation	4	Q8R5C5	C34	reversible
BF1	Neutrophil degranulation	5	Q07113	C724	reversible
BF1	Neutrophil degranulation	5	Q07113	C807	reversible
BF1	Neutrophil degranulation	5	Q07113	C886	reversible
BF1	Neutrophil degranulation	5	Q07113	C2241	reversible
BF1	Neutrophil degranulation	1	Q3TXS7	C571	reversible
BF1	Neutrophil degranulation	2	P52480	C317	so3h
BF1	Neutrophil degranulation	3	Q08857	C313	reversible
BF1	Neutrophil degranulation	4	Q01853	C105	reversible
BF1	Neutrophil degranulation	5	Q01853	C572	reversible
BF1	Neutrophil degranulation	1	Q8CI94	C326	reversible
BF1	Neutrophil degranulation	2	P01027	C727	reversible
BF1	Neutrophil degranulation	3	Q8VDM4	C448	reversible
BF1	Neutrophil degranulation	4	P63001	C178	reversible
BF1	Neutrophil degranulation	5	P40124	C92	reversible
BF1	Neutrophil degranulation	1	P18242	C117	reversible
BF1	Neutrophil degranulation	2	Q9DBR3	C288	so3h
BF1	Neutrophil degranulation	2	Q9DBR3	C3	reversible
BF1	Neutrophil degranulation	3	O89103	C297	reversible
BF1	Neutrophil degranulation	4	P12382	C351	reversible
BF1	Neutrophil degranulation	5	P63001	C157	reversible
BF1	Neutrophil degranulation	1	P50543	C8	so3h
BF1	Neutrophil degranulation	2	Q8BWQ6	C303	so3h
BF1	Neutrophil degranulation	2	Q8BWQ6	C315	so3h
BF1	Neutrophil degranulation	3	P55849	C673	so2h
BF1	Neutrophil degranulation	4	Q01768	C109	reversible
BF1	Neutrophil degranulation	5	P13020	C670	reversible
BF1	Neutrophil degranulation	1	Q07113	C513	reversible
BF1	Neutrophil degranulation	2	P17742	C62	reversible
BF1	Neutrophil degranulation	3	Q07113	C1509	reversible
BF1	Neutrophil degranulation	4	P58252	C41	reversible
BF1	Neutrophil degranulation	4	P58252	C466	reversible
BF1	Neutrophil degranulation	5	P58252	C728	reversible
BF1	Neutrophil degranulation	1	Q01853	C522	reversible
BF1	Neutrophil degranulation	2	Q9JHW2	C170	reversible
BF1	Neutrophil degranulation	3	P17742	C161	reversible
BF1	Neutrophil degranulation	4	Q08857	C272	reversible
BF1	Neutrophil degranulation	5	Q91W90	C366	reversible
BF1	Neutrophil degranulation	1	P12382	C708	reversible

BF1	Neutrophil degranulation	4	P52480	C358	reversible
BF1	Neutrophil degranulation	4	P52480	C49	reversible
BF1	Neutrophil degranulation	5	P63011	C184	reversible
BF1	Neutrophil degranulation	1	Q7TMB8	C1087	so2h
BF1	Neutrophil degranulation	4	P05064	C178	reversible
BF1	Neutrophil degranulation	5	P01027	C1101	reversible
BF1	Neutrophil degranulation	5	P01027	C1389	reversible
BF1	Neutrophil degranulation	5	P01027	C1458	reversible
BF1	Neutrophil degranulation	5	P01027	C1489	reversible
BF1	Neutrophil degranulation	5	P01027	C1537	reversible
BF1	Neutrophil degranulation	5	P01027	C559	reversible
BF1	Neutrophil degranulation	5	P01027	C626	reversible
BF1	Neutrophil degranulation	5	P01027	C720	reversible
BF1	Neutrophil degranulation	5	P01027	C728	reversible
BF1	Neutrophil degranulation	5	P01027	C816	reversible
BF1	Neutrophil degranulation	5	P01027	C873	reversible
BF1	Neutrophil degranulation	1	O35598	C436	reversible
BF1	Neutrophil degranulation	4	Q9D0F9	C160	reversible
BF1	Neutrophil degranulation	5	Q9JHW2	C44	reversible
BF1	Neutrophil degranulation	1	Q08857	C311	so3h
BF1	Neutrophil degranulation	1	Q08857	C313	so2h
BF1	Neutrophil degranulation	4	P63017	C603	reversible
BF1	Neutrophil degranulation	1	Q9DBJ1	C153	reversible
BF1	Neutrophil degranulation	1	O54734	C130	reversible
BF1	Neutrophil degranulation	1	P52480	C326	reversible
BF1	Neutrophil degranulation	1	P05064	C202	reversible
BF1	Neutrophil degranulation	1	P05064	C339	reversible
BF1	Neutrophil degranulation	1	P05064	C73	reversible
BF1	Neutrophil degranulation	1	P01027	C1511	reversible
BF1	Neutrophil degranulation	1	Q3UDW8	C533	so2h
BF1	Neutrophil degranulation	1	Q3UDW8	C532	so2h
BF1	Neutrophil degranulation	1	Q9D0F9	C101	reversible
BF1	Neutrophil degranulation	1	P63017	C17	reversible
BF1	Neutrophil degranulation	1	Q9JHW2	C97	reversible
BF2	Response to elevated platelet cytosolic Ca2+	1	Q9JI91	C48	reversible
BF2	Response to elevated platelet cytosolic Ca2+	2	P07724	C385	so3h
BF2	Response to elevated platelet cytosolic Ca2+	3	P07724	C289	so3h

BF2	Response to elevated platelet cytosolic Ca2+	4	P07724	C114	reversible
BF2	Response to elevated platelet cytosolic Ca2+	4	P07724	C115	reversible
BF2	Response to elevated platelet cytosolic Ca2+	4	P07724	C148	reversible
BF2	Response to elevated platelet cytosolic Ca2+	4	P07724	C201	reversible
BF2	Response to elevated platelet cytosolic Ca2+	4	P07724	C289	reversible
BF2	Response to elevated platelet cytosolic Ca2+	4	P07724	C303	reversible
BF2	Response to elevated platelet cytosolic Ca2+	4	P07724	C313	reversible
BF2	Response to elevated platelet cytosolic Ca2+	4	P07724	C340	reversible
BF2	Response to elevated platelet cytosolic Ca2+	4	P07724	C77	reversible
BF2	Response to elevated platelet cytosolic Ca2+	5	Q9JI91	C187	reversible
BF2	Response to elevated platelet cytosolic Ca2+	1	P07724	C393	reversible
BF2	Response to elevated platelet cytosolic Ca2+	1	P07724	C461	reversible
BF2	Response to elevated platelet cytosolic Ca2+	1	P07724	C462	reversible
BF2	Response to elevated platelet cytosolic Ca2+	1	P07724	C472	reversible
BF2	Response to elevated platelet cytosolic Ca2+	1	P07724	C472	so3h
BF2	Response to elevated platelet cytosolic Ca2+	1	P07724	C501	reversible
BF2	Response to elevated platelet cytosolic Ca2+	1	P07724	C538	reversible
BF2	Response to elevated platelet cytosolic Ca2+	1	P07724	C582	reversible
BF2	Response to elevated platelet cytosolic Ca2+	1	P07724	C583	reversible
BF2	Response to elevated platelet cytosolic Ca2+	1	P07724	C591	reversible
BF2	Response to elevated platelet cytosolic Ca2+	1	P07724	C86	reversible
BF2	Response to elevated platelet cytosolic Ca2+	1	P07724	C384	reversible
BF2	Response to elevated platelet cytosolic Ca2+	1	P07724	C385	reversible
BF2	Response to elevated platelet cytosolic Ca2+	2	Q9CZ30	C187	reversible
BF2	Response to elevated platelet cytosolic Ca2+	3	Q08481	C470	reversible
BF2	Response to elevated platelet cytosolic Ca2+	4	Q08857	C272	reversible
BF2	Response to elevated platelet cytosolic Ca2+	5	P07724	C302	reversible

BF2	Response to elevated platelet cytosolic Ca2+	5	P07724	C485	reversible
BF2	Response to elevated platelet cytosolic Ca2+	5	P07724	C500	reversible
BF2	Response to elevated platelet cytosolic Ca2+	5	P07724	C511	reversible
BF2	Response to elevated platelet cytosolic Ca2+	5	P07724	C99	reversible
BF2	Response to elevated platelet cytosolic Ca2+	1	P20918	C73	reversible
BF2	Response to elevated platelet cytosolic Ca2+	1	P20918	C257	reversible
BF2	Response to elevated platelet cytosolic Ca2+	1	P20918	C335	reversible
BF2	Response to elevated platelet cytosolic Ca2+	2	P20918	C586	reversible
BF2	Response to elevated platelet cytosolic Ca2+	3	Q08857	C313	reversible
BF2	Response to elevated platelet cytosolic Ca2+	4	P20918	C103	reversible
BF2	Response to elevated platelet cytosolic Ca2+	5	Q9CZ30	C75	reversible
BF2	Response to elevated platelet cytosolic Ca2+	1	P63260	C217	reversible
BF2	Response to elevated platelet cytosolic Ca2+	2	E9PV24	C438	reversible
BF2	Response to elevated platelet cytosolic Ca2+	4	P05064	C178	reversible
BF2	Response to elevated platelet cytosolic Ca2+	5	Q9ESB3	C103	reversible
BF2	Response to elevated platelet cytosolic Ca2+	5	Q9ESB3	C216	reversible
BF2	Response to elevated platelet cytosolic Ca2+	1	Q8VCM7	C48	reversible
BF2	Response to elevated platelet cytosolic Ca2+	1	Q8VCM7	C164	reversible
BF2	Response to elevated platelet cytosolic Ca2+	2	P63260	C217	so3h
BF2	Response to elevated platelet cytosolic Ca2+	4	Q921I1	C373	reversible
BF2	Response to elevated platelet cytosolic Ca2+	5	Q8VCM7	C34	reversible
BF2	Response to elevated platelet cytosolic Ca2+	1	A2ASS6	C6705	so2h
BF2	Response to elevated platelet cytosolic Ca2+	2	Q921I1	C526	so2h
BF2	Response to elevated platelet cytosolic Ca2+	4	O88342	C325	reversible
BF2	Response to elevated platelet cytosolic Ca2+	5	Q921I1	C156	reversible
BF2	Response to elevated platelet cytosolic Ca2+	5	Q921I1	C177	reversible
BF2	Response to elevated platelet cytosolic Ca2+	5	Q921I1	C180	reversible

BF2	Response to elevated platelet cytosolic Ca2+	5	Q921I1	C246	reversible
BF2	Response to elevated platelet cytosolic Ca2+	5	Q921I1	C260	reversible
BF2	Response to elevated platelet cytosolic Ca2+	5	Q921I1	C28	reversible
BF2	Response to elevated platelet cytosolic Ca2+	5	Q921I1	C350	reversible
BF2	Response to elevated platelet cytosolic Ca2+	5	Q921I1	C363	reversible
BF2	Response to elevated platelet cytosolic Ca2+	5	Q921I1	C386	reversible
BF2	Response to elevated platelet cytosolic Ca2+	5	Q921I1	C506	reversible
BF2	Response to elevated platelet cytosolic Ca2+	5	Q921I1	C597	reversible
BF2	Response to elevated platelet cytosolic Ca2+	5	Q921I1	C633	reversible
BF2	Response to elevated platelet cytosolic Ca2+	5	Q921I1	C638	reversible
BF2	Response to elevated platelet cytosolic Ca2+	5	Q921I1	C692	reversible
BF2	Response to elevated platelet cytosolic Ca2+	1	P11276	C136	reversible
BF2	Response to elevated platelet cytosolic Ca2+	2	P11276	C374	reversible
BF2	Response to elevated platelet cytosolic Ca2+	1	Q8K0E8	C213	so2h
BF2	Response to elevated platelet cytosolic Ca2+	2	P26039	C1661	so2h
BF2	Response to elevated platelet cytosolic Ca2+	2	P26039	C1671	so3h
BF2	Response to elevated platelet cytosolic Ca2+	1	Q8C522	C34	reversible
BF2	Response to elevated platelet cytosolic Ca2+	1	Q08857	C311	so3h
BF2	Response to elevated platelet cytosolic Ca2+	1	Q08857	C313	so2h
BF2	Response to elevated platelet cytosolic Ca2+	1	P05064	C202	reversible
BF2	Response to elevated platelet cytosolic Ca2+	1	P05064	C339	reversible
BF2	Response to elevated platelet cytosolic Ca2+	1	P05064	C73	reversible
BF2	Response to elevated platelet cytosolic Ca2+	1	E9PV24	C56	reversible
BF2	Response to elevated platelet cytosolic Ca2+	1	Q9ESB3	C89	reversible
BF2	Response to elevated platelet cytosolic Ca2+	1	P20444	C143	so2h
BF2	Response to elevated platelet cytosolic Ca2+	1	Q921I1	C67	reversible
BF2	Response to elevated platelet cytosolic Ca2+	1	O88342	C225	so2h

BF3	Extracellular matrix organization	1	Q61554	C1182	reversible
BF3	Extracellular matrix organization	1	Q61554	C494	reversible
BF3	Extracellular matrix organization	1	Q61554	C1008	reversible
BF3	Extracellular matrix organization	1	Q61554	C1032	so3h
BF3	Extracellular matrix organization	1	Q61554	C1039	so3h
BF3	Extracellular matrix organization	2	Q61554	C2251	reversible
BF3	Extracellular matrix organization	2	Q61554	C845	reversible
BF3	Extracellular matrix organization	2	Q61554	C1081	reversible
BF3	Extracellular matrix organization	3	Q61554	C2061	reversible
BF3	Extracellular matrix organization	4	Q61554	C1847	reversible
BF3	Extracellular matrix organization	5	P02463	C1616	reversible
BF3	Extracellular matrix organization	5	P02463	C1493	reversible
BF3	Extracellular matrix organization	1	Q61555	C512	so3h
BF3	Extracellular matrix organization	2	Q60675	C1141	reversible
BF3	Extracellular matrix organization	2	Q60675	C1502	reversible
BF3	Extracellular matrix organization	2	Q60675	C349	reversible
BF3	Extracellular matrix organization	2	Q60675	C3115	reversible
BF3	Extracellular matrix organization	2	Q60675	C831	reversible
BF3	Extracellular matrix organization	3	P97927	C1222	reversible
BF3	Extracellular matrix organization	4	P10493	C616	reversible
BF3	Extracellular matrix organization	5	P10605	C319	reversible
BF3	Extracellular matrix organization	1	Q8BKV0	C90	so2h
BF3	Extracellular matrix organization	2	P26262	C419	reversible
BF3	Extracellular matrix organization	2	P26262	C383	reversible
BF3	Extracellular matrix organization	3	Q61555	C2335	so3h
BF3	Extracellular matrix organization	4	P09055	C618	reversible
BF3	Extracellular matrix organization	5	Q61554	C2413	reversible
BF3	Extracellular matrix organization	5	Q61554	C460	reversible
BF3	Extracellular matrix organization	5	Q61554	C2489	reversible
BF3	Extracellular matrix organization	1	P55066	C1160	so3h
BF3	Extracellular matrix organization	2	P55066	C252	so2h
BF3	Extracellular matrix organization	3	Q60675	C1447	reversible
BF3	Extracellular matrix organization	4	Q61292	C906	reversible
BF3	Extracellular matrix organization	5	Q60675	C493	reversible
BF3	Extracellular matrix organization	1	P20918	C73	reversible
BF3	Extracellular matrix organization	1	P20918	C257	reversible
BF3	Extracellular matrix organization	1	P20918	C335	reversible
BF3	Extracellular matrix organization	2	O08529	C39	reversible
BF3	Extracellular matrix organization	3	O35566	C185	reversible

BF3	Extracellular matrix organization	4	P20918	C103	reversible
BF3	Extracellular matrix organization	5	A2ASQ1	C441	reversible
BF3	Extracellular matrix organization	1	P18242	C117	reversible
BF3	Extracellular matrix organization	2	P34960	C3	reversible
BF3	Extracellular matrix organization	3	Q08481	C470	reversible
BF3	Extracellular matrix organization	4	Q61739	C881	reversible
BF3	Extracellular matrix organization	5	Q8K4G1	C599	reversible
BF3	Extracellular matrix organization	1	A2ASQ1	C1921	reversible
BF3	Extracellular matrix organization	2	P20918	C586	reversible
BF3	Extracellular matrix organization	3	O88322	C1317	reversible
BF3	Extracellular matrix organization	4	P18242	C288	reversible
BF3	Extracellular matrix organization	5	Q8VCM7	C34	reversible
BF3	Extracellular matrix organization	1	Q8VCM7	C48	reversible
BF3	Extracellular matrix organization	1	Q8VCM7	C164	reversible
BF3	Extracellular matrix organization	2	P11276	C374	reversible
BF3	Extracellular matrix organization	4	Q05793	C2313	reversible
BF3	Extracellular matrix organization	5	Q05793	C1156	reversible
BF3	Extracellular matrix organization	5	Q05793	C3137	reversible
BF3	Extracellular matrix organization	5	Q05793	C811	reversible
BF3	Extracellular matrix organization	5	Q05793	C892	reversible
BF3	Extracellular matrix organization	1	Q05793	C2456	reversible
BF3	Extracellular matrix organization	1	Q05793	C2456	so3h
BF3	Extracellular matrix organization	2	Q3V3R4	C130	reversible
BF3	Extracellular matrix organization	5	Q3V1T4	C571	reversible
BF3	Extracellular matrix organization	1	O88839	C668	so2h
BF3	Extracellular matrix organization	2	Q05793	C731	reversible
BF3	Extracellular matrix organization	2	Q05793	C1140	reversible
BF3	Extracellular matrix organization	5	P10493	C423	reversible
BF3	Extracellular matrix organization	1	A2ARA8	C933	so2h
BF3	Extracellular matrix organization	1	A2ARA8	C923	so2h
BF3	Extracellular matrix organization	1	A2ARA8	C21	so3h
BF3	Extracellular matrix organization	1	A2ARA8	C22	so3h
BF3	Extracellular matrix organization	2	P33434	C21	so3h
BF3	Extracellular matrix organization	2	P33434	C22	so3h
BF3	Extracellular matrix organization	5	Q61292	C505	reversible
BF3	Extracellular matrix organization	1	Q61738	C103	reversible
BF3	Extracellular matrix organization	2	Q80Z71	C1486	reversible
BF3	Extracellular matrix organization	5	Q61739	C154	reversible
BF3	Extracellular matrix organization	1	Q9QZR9	C1675	reversible

BF3	Extracellular matrix organization	2	P10493	C409	reversible
BF3	Extracellular matrix organization	2	P10493	C712	reversible
BF3	Extracellular matrix organization	5	P02469	C170	reversible
BF3	Extracellular matrix organization	5	P02469	C1785	reversible
BF3	Extracellular matrix organization	5	P02469	C190	reversible
BF3	Extracellular matrix organization	1	P02468	C349	reversible
BF3	Extracellular matrix organization	1	P02468	C472	reversible
BF3	Extracellular matrix organization	1	P02468	C1013	reversible
BF3	Extracellular matrix organization	1	P02468	C859	so2h
BF3	Extracellular matrix organization	1	P02468	C954	reversible
BF3	Extracellular matrix organization	2	Q61738	C546	reversible
BF3	Extracellular matrix organization	5	O88792	C49	reversible
BF3	Extracellular matrix organization	1	P08122	C1653	reversible
BF3	Extracellular matrix organization	2	A6H584	C1974	so2h
BF3	Extracellular matrix organization	1	E9PV24	C56	reversible
BF3	Extracellular matrix organization	2	P08122	C1660	reversible
BF3	Extracellular matrix organization	1	P29788	C179	so2h
BF3	Extracellular matrix organization	2	E9PV24	C438	reversible
BF3	Extracellular matrix organization	1	Q60675	C1544	reversible
BF3	Extracellular matrix organization	1	Q60675	C784	reversible
BF3	Extracellular matrix organization	2	Q9WVH9	C53	reversible
BF3	Extracellular matrix organization	1	P37889	C754	reversible
BF3	Extracellular matrix organization	1	P11276	C136	reversible
BF3	Extracellular matrix organization	1	Q3V3R4	C548	so2h
BF3	Extracellular matrix organization	1	Q8K0E8	C213	so2h
BF3	Extracellular matrix organization	1	P10493	C1230	so3h
BF3	Extracellular matrix organization	1	P10493	C1232	so3h
BF3	Extracellular matrix organization	1	O35598	C436	reversible
BF3	Extracellular matrix organization	1	Q61292	C50	reversible
BF3	Extracellular matrix organization	1	P20444	C143	so2h
BF4	Protein Translation	1	P51410	C134	reversible
BF4	Protein Translation	2	P62908	C97	reversible
BF4	Protein Translation	3	P35979	C141	reversible
BF4	Protein Translation	4	P58252	C41	reversible
BF4	Protein Translation	4	P58252	C466	reversible
BF4	Protein Translation	5	P97351	C96	reversible
BF4	Protein Translation	1	P29341	C132	reversible
BF4	Protein Translation	2	Q9CXW4	C72	reversible
BF4	Protein Translation	3	P58252	C567	reversible

BF4	Protein Translation	5	P62908	C119	reversible
BF4	Protein Translation	5	P62908	C134	reversible
BF4	Protein Translation	1	Q8R2Q4	C329	reversible
BF4	Protein Translation	2	P58252	C290	reversible
BF4	Protein Translation	2	P58252	C591	reversible
BF4	Protein Translation	2	P58252	C693	reversible
BF4	Protein Translation	3	P47962	C62	so2h
BF4	Protein Translation	5	P58252	C728	reversible
BF4	Protein Translation	1	Q99N87	C326	reversible
BF4	Protein Translation	1	Q99N87	C318	so3h
BF4	Protein Translation	2	Q6ZWV3	C80	so2h
BF4	Protein Translation	3	Q91VM9	C156	reversible
BF4	Protein Translation	5	Q6ZWV3	C105	reversible
BF4	Protein Translation	2	Q9D0G0	C196	reversible
BF4	Protein Translation	3	Q9D7N3	C227	reversible
BF4	Protein Translation	5	P62717	C109	reversible
BF4	Protein Translation	2	P62702	C181	reversible
BF4	Protein Translation	5	P62702	C41	reversible
BF4	Protein Translation	2	Q9CQF0	C50	so3h
BF4	Protein Translation	5	P10126	C234	reversible
BF4	Protein Translation	2	P60867	C36	reversible
BF4	Protein Translation	5	P62281	C60	reversible
BF4	Protein Translation	2	P62754	C12	reversible
BF4	Protein Translation	5	P25444	C229	reversible
BF4	Protein Translation	2	Q8BJU9	C267	reversible
BF5	Post-translational protein phosphorylation	1	Q61554	C1182	reversible
BF5	Post-translational protein phosphorylation	1	Q61554	C494	reversible
BF5	Post-translational protein phosphorylation	1	Q61554	C1008	reversible
BF5	Post-translational protein phosphorylation	1	Q61554	C1032	so3h
BF5	Post-translational protein phosphorylation	1	Q61554	C1039	so3h
BF5	Post-translational protein phosphorylation	2	Q61147	C275	reversible
BF5	Post-translational protein phosphorylation	3	P21956	C303	reversible
BF5	Post-translational protein phosphorylation	4	P21956	C290	reversible
BF5	Post-translational protein phosphorylation	5	Q61554	C2413	reversible
BF5	Post-translational protein phosphorylation	5	Q61554	C460	reversible
BF5	Post-translational protein phosphorylation	5	Q61554	C2489	reversible
BF5	Post-translational protein phosphorylation	1	Q61147	C173	reversible
BF5	Post-translational protein phosphorylation	2	Q61554	C2251	reversible
BF5	Post-translational protein phosphorylation	2	Q61554	C845	reversible

BF5	Post-translational protein phosphorylation	2	Q61554	C1081	reversible
BF5	Post-translational protein phosphorylation	3	Q61554	C2061	reversible
BF5	Post-translational protein phosphorylation	4	Q61554	C1847	reversible
BF5	Post-translational protein phosphorylation	5	Q61147	C199	reversible
BF5	Post-translational protein phosphorylation	5	Q61147	C713	reversible
BF5	Post-translational protein phosphorylation	1	P07724	C393	reversible
BF5	Post-translational protein phosphorylation	1	P07724	C461	reversible
BF5	Post-translational protein phosphorylation	1	P07724	C462	reversible
BF5	Post-translational protein phosphorylation	1	P07724	C472	reversible
BF5	Post-translational protein phosphorylation	1	P07724	C472	so3h
BF5	Post-translational protein phosphorylation	1	P07724	C501	reversible
BF5	Post-translational protein phosphorylation	1	P07724	C538	reversible
BF5	Post-translational protein phosphorylation	1	P07724	C582	reversible
BF5	Post-translational protein phosphorylation	1	P07724	C583	reversible
BF5	Post-translational protein phosphorylation	1	P07724	C591	reversible
BF5	Post-translational protein phosphorylation	1	P07724	C86	reversible
BF5	Post-translational protein phosphorylation	1	P07724	C384	reversible
BF5	Post-translational protein phosphorylation	1	P07724	C385	reversible
BF5	Post-translational protein phosphorylation	2	P07724	C385	so3h
BF5	Post-translational protein phosphorylation	3	P07724	C289	so3h
BF5	Post-translational protein phosphorylation	4	P07724	C114	reversible
BF5	Post-translational protein phosphorylation	4	P07724	C115	reversible
BF5	Post-translational protein phosphorylation	4	P07724	C148	reversible
BF5	Post-translational protein phosphorylation	4	P07724	C201	reversible
BF5	Post-translational protein phosphorylation	4	P07724	C289	reversible
BF5	Post-translational protein phosphorylation	4	P07724	C303	reversible
BF5	Post-translational protein phosphorylation	4	P07724	C313	reversible
BF5	Post-translational protein phosphorylation	4	P07724	C340	reversible
BF5	Post-translational protein phosphorylation	4	P07724	C77	reversible
BF5	Post-translational protein phosphorylation	5	P07724	C302	reversible
BF5	Post-translational protein phosphorylation	5	P07724	C485	reversible
BF5	Post-translational protein phosphorylation	5	P07724	C500	reversible
BF5	Post-translational protein phosphorylation	5	P07724	C511	reversible
BF5	Post-translational protein phosphorylation	5	P07724	C99	reversible
BF5	Post-translational protein phosphorylation	1	Q8VCM7	C48	reversible
BF5	Post-translational protein phosphorylation	1	Q8VCM7	C164	reversible
BF5	Post-translational protein phosphorylation	2	P32261	C54	reversible
BF5	Post-translational protein phosphorylation	3	P32261	C463	reversible
BF5	Post-translational protein phosphorylation	4	P16045	C61	reversible

BF5	Post-translational protein phosphorylation	5	Q61292	C505	reversible
BF5	Post-translational protein phosphorylation	1	P11276	C136	reversible
BF5	Post-translational protein phosphorylation	2	P01029	C1721	reversible
BF5	Post-translational protein phosphorylation	4	Q61292	C906	reversible
BF5	Post-translational protein phosphorylation	5	P02469	C170	reversible
BF5	Post-translational protein phosphorylation	5	P02469	C1785	reversible
BF5	Post-translational protein phosphorylation	5	P02469	C190	reversible
BF5	Post-translational protein phosphorylation	1	O35887	C13	so3h
BF5	Post-translational protein phosphorylation	2	E9PV24	C438	reversible
BF5	Post-translational protein phosphorylation	4	Q921I1	C373	reversible
BF5	Post-translational protein phosphorylation	5	P01027	C1101	reversible
BF5	Post-translational protein phosphorylation	5	P01027	C1389	reversible
BF5	Post-translational protein phosphorylation	5	P01027	C1458	reversible
BF5	Post-translational protein phosphorylation	5	P01027	C1489	reversible
BF5	Post-translational protein phosphorylation	5	P01027	C1537	reversible
BF5	Post-translational protein phosphorylation	5	P01027	C559	reversible
BF5	Post-translational protein phosphorylation	5	P01027	C626	reversible
BF5	Post-translational protein phosphorylation	5	P01027	C720	reversible
BF5	Post-translational protein phosphorylation	5	P01027	C728	reversible
BF5	Post-translational protein phosphorylation	5	P01027	C816	reversible
BF5	Post-translational protein phosphorylation	5	P01027	C873	reversible
BF5	Post-translational protein phosphorylation	1	P21956	C308	reversible
BF5	Post-translational protein phosphorylation	2	P01027	C727	reversible
BF5	Post-translational protein phosphorylation	5	Q8VCM7	C34	reversible
BF5	Post-translational protein phosphorylation	1	O35598	C436	reversible
BF5	Post-translational protein phosphorylation	2	Q921I1	C526	so2h
BF5	Post-translational protein phosphorylation	5	Q921I1	C156	reversible
BF5	Post-translational protein phosphorylation	5	Q921I1	C177	reversible
BF5	Post-translational protein phosphorylation	5	Q921I1	C180	reversible
BF5	Post-translational protein phosphorylation	5	Q921I1	C246	reversible
BF5	Post-translational protein phosphorylation	5	Q921I1	C260	reversible
BF5	Post-translational protein phosphorylation	5	Q921I1	C28	reversible
BF5	Post-translational protein phosphorylation	5	Q921I1	C350	reversible
BF5	Post-translational protein phosphorylation	5	Q921I1	C363	reversible
BF5	Post-translational protein phosphorylation	5	Q921I1	C386	reversible
BF5	Post-translational protein phosphorylation	5	Q921I1	C506	reversible
BF5	Post-translational protein phosphorylation	5	Q921I1	C597	reversible
BF5	Post-translational protein phosphorylation	5	Q921I1	C633	reversible
BF5	Post-translational protein phosphorylation	5	Q921I1	C638	reversible

BF5	Post-translational protein phosphorylation	5	Q921I1	C692	reversible
BF5	Post-translational protein phosphorylation	1	Q61292	C50	reversible
BF5	Post-translational protein phosphorylation	2	P11276	C374	reversible
BF5	Post-translational protein phosphorylation	1	P02468	C349	reversible
BF5	Post-translational protein phosphorylation	1	P02468	C472	reversible
BF5	Post-translational protein phosphorylation	1	P02468	C1013	reversible
BF5	Post-translational protein phosphorylation	1	P02468	C859	so2h
BF5	Post-translational protein phosphorylation	1	P02468	C954	reversible
BF5	Post-translational protein phosphorylation	1	P01027	C1511	reversible
BF5	Post-translational protein phosphorylation	1	E9PV24	C56	reversible
BF5	Post-translational protein phosphorylation	1	Q921I1	C67	reversible
BF6	Glucose metabolism	1	P17710	C214	reversible
BF6	Glucose metabolism	2	P17710	C869	reversible
BF6	Glucose metabolism	2	P17710	C890	so3h
BF6	Glucose metabolism	3	P09411	C99	reversible
BF6	Glucose metabolism	4	Q9CR62	C184	reversible
BF6	Glucose metabolism	5	P17182	C337	reversible
BF6	Glucose metabolism	1	P12382	C708	reversible
BF6	Glucose metabolism	2	Q05920	C372	reversible
BF6	Glucose metabolism	3	O08528	C368	reversible
BF6	Glucose metabolism	3	O08528	C375	reversible
BF6	Glucose metabolism	3	O08528	C909	reversible
BF6	Glucose metabolism	4	P16858	C154	reversible
BF6	Glucose metabolism	4	P16858	C22	reversible
BF6	Glucose metabolism	5	Q9QXX4	C504	reversible
BF6	Glucose metabolism	1	P06745	C404	reversible
BF6	Glucose metabolism	2	P14152	C137	so3h
BF6	Glucose metabolism	3	Q8BH59	C505	reversible
BF6	Glucose metabolism	4	O70250	C153	reversible
BF6	Glucose metabolism	5	Q8BH59	C435	reversible
BF6	Glucose metabolism	1	Q9ERU9	C1366	reversible
BF6	Glucose metabolism	2	P16858	C150	so2h
BF6	Glucose metabolism	2	P16858	C154	so3h
BF6	Glucose metabolism	4	P17751	C268	reversible
BF6	Glucose metabolism	1	Q9DBJ1	C153	reversible
BF6	Glucose metabolism	2	P52480	C317	so3h
BF6	Glucose metabolism	4	P17710	C684	reversible
BF6	Glucose metabolism	1	P16858	C150	reversible
BF6	Glucose metabolism	1	P16858	C245	reversible

BF6	Glucose metabolism	2	P17182	C339	reversible
BF6	Glucose metabolism	4	P12382	C351	reversible
BF6	Glucose metabolism	1	P52480	C326	reversible
BF6	Glucose metabolism	2	Q9WUA3	C717	so3h
BF6	Glucose metabolism	4	P14152	C137	reversible
BF6	Glucose metabolism	4	P14152	C154	reversible
BF6	Glucose metabolism	1	P05064	C202	reversible
BF6	Glucose metabolism	1	P05064	C339	reversible
BF6	Glucose metabolism	1	P05064	C73	reversible
BF6	Glucose metabolism	2	P08249	C93	so3h
BF6	Glucose metabolism	2	P08249	C89	so2h
BF6	Glucose metabolism	4	P52480	C358	reversible
BF6	Glucose metabolism	4	P52480	C49	reversible
BF6	Glucose metabolism	1	P21550	C337	reversible
BF6	Glucose metabolism	4	P09411	C50	reversible
BF6	Glucose metabolism	1	Q9WUA3	C122	reversible
BF6	Glucose metabolism	4	P05064	C178	reversible
BF6	Glucose metabolism	1	P05201	C391	reversible
BF6	Glucose metabolism	4	P21550	C339	reversible
BF6	Glucose metabolism	1	P05202	C295	reversible
BF6	Glucose metabolism	1	P05202	C382	reversible
BF6	Glucose metabolism	4	P08249	C212	reversible
BF6	Glucose metabolism	4	P08249	C275	reversible
BF6	Glucose metabolism	4	P08249	C285	reversible
BF6	Glucose metabolism	4	P08249	C89	reversible
BF6	Glucose metabolism	4	P08249	C93	reversible
BF6	Glucose metabolism	4	P47857	C114	reversible
BF6	Glucose metabolism	4	P47857	C709	reversible
BF6	Glucose metabolism	4	P05202	C106	reversible
BF6	Glucose metabolism	4	P05202	C187	reversible
BF7	Pyruvate metabolism and Citric Acid (TCA) cycle	1	Q8BMF3	C450	reversible
BF7	Pyruvate metabolism and Citric Acid (TCA) cycle	1	Q8BMF3	C82	reversible
BF7	Pyruvate metabolism and Citric Acid (TCA) cycle	2	P16125	C164	reversible
BF7	Pyruvate metabolism and Citric Acid (TCA) cycle	3	Q60597	C395	reversible
BF7	Pyruvate metabolism and Citric Acid (TCA) cycle	4	Q60597	C566	reversible
BF7	Pyruvate metabolism and Citric Acid (TCA) cycle	4	Q60597	C904	reversible

BF7	Pyruvate metabolism and Citric Acid (TCA) cycle	5	P06801	C47	reversible
BF7	Pyruvate metabolism and Citric Acid (TCA) cycle	1	Q8R0F8	C115	reversible
BF7	Pyruvate metabolism and Citric Acid (TCA) cycle	2	Q99KE1	C190	so3h
BF7	Pyruvate metabolism and Citric Acid (TCA) cycle	3	Q9D6R2	C359	reversible
BF7	Pyruvate metabolism and Citric Acid (TCA) cycle	4	Q9D6R2	C222	reversible
BF7	Pyruvate metabolism and Citric Acid (TCA) cycle	4	Q9D6R2	C331	reversible
BF7	Pyruvate metabolism and Citric Acid (TCA) cycle	4	Q9D6R2	C351	reversible
BF7	Pyruvate metabolism and Citric Acid (TCA) cycle	5	O08749	C477	reversible
BF7	Pyruvate metabolism and Citric Acid (TCA) cycle	1	P06151	C163	reversible
BF7	Pyruvate metabolism and Citric Acid (TCA) cycle	2	Q9CZB0	C70	reversible
BF7	Pyruvate metabolism and Citric Acid (TCA) cycle	3	P06801	C468	reversible
BF7	Pyruvate metabolism and Citric Acid (TCA) cycle	4	Q60932	C140	reversible
BF7	Pyruvate metabolism and Citric Acid (TCA) cycle	4	Q60932	C245	reversible
BF7	Pyruvate metabolism and Citric Acid (TCA) cycle	5	Q9WUM5	C181	reversible
BF7	Pyruvate metabolism and Citric Acid (TCA) cycle	5	Q9WUM5	C60	reversible
BF7	Pyruvate metabolism and Citric Acid (TCA) cycle	1	Q9D2G2	C105	reversible
BF7	Pyruvate metabolism and Citric Acid (TCA) cycle	2	P06151	C163	so3h
BF7	Pyruvate metabolism and Citric Acid (TCA) cycle	3	Q91YP0	C273	reversible
BF7	Pyruvate metabolism and Citric Acid (TCA) cycle	4	O08749	C80	reversible
BF7	Pyruvate metabolism and Citric Acid (TCA) cycle	4	O08749	C85	reversible
BF7	Pyruvate metabolism and Citric Acid (TCA) cycle	5	Q9CPU0	C139	reversible
BF7	Pyruvate metabolism and Citric Acid (TCA) cycle	1	Q9CZU6	C359	so2h
BF7	Pyruvate metabolism and Citric Acid (TCA) cycle	2	Q99KI0	C126	so3h
BF7	Pyruvate metabolism and Citric Acid (TCA) cycle	2	Q99KI0	C385	so3h
BF7	Pyruvate metabolism and Citric Acid (TCA) cycle	3	Q8BMF3	C275	reversible
BF7	Pyruvate metabolism and Citric Acid (TCA) cycle	4	Q9D051	C263	reversible
BF7	Pyruvate metabolism and Citric Acid (TCA) cycle	5	P70404	C333	reversible

BF7	Pyruvate metabolism and Citric Acid (TCA) cycle	1	Q9D051	C169	reversible
BF7	Pyruvate metabolism and Citric Acid (TCA) cycle	1	Q9D051	C161	reversible
BF7	Pyruvate metabolism and Citric Acid (TCA) cycle	2	Q8BFP9	C238	reversible
BF7	Pyruvate metabolism and Citric Acid (TCA) cycle	3	Q9Z2I9	C270	reversible
BF7	Pyruvate metabolism and Citric Acid (TCA) cycle	4	P35486	C181	reversible
BF7	Pyruvate metabolism and Citric Acid (TCA) cycle	4	P35486	C261	reversible
BF7	Pyruvate metabolism and Citric Acid (TCA) cycle	4	P35486	C273	reversible
BF7	Pyruvate metabolism and Citric Acid (TCA) cycle	5	Q8K2B3	C357	reversible
BF7	Pyruvate metabolism and Citric Acid (TCA) cycle	1	Q8BMF4	C581	reversible
BF7	Pyruvate metabolism and Citric Acid (TCA) cycle	2	P08249	C93	so3h
BF7	Pyruvate metabolism and Citric Acid (TCA) cycle	2	P08249	C89	so2h
BF7	Pyruvate metabolism and Citric Acid (TCA) cycle	3	Q99KI0	C410	reversible
BF7	Pyruvate metabolism and Citric Acid (TCA) cycle	4	Q8BMF4	C290	reversible
BF7	Pyruvate metabolism and Citric Acid (TCA) cycle	4	Q8BMF4	C483	reversible
BF7	Pyruvate metabolism and Citric Acid (TCA) cycle	1	P54071	C336	reversible
BF7	Pyruvate metabolism and Citric Acid (TCA) cycle	2	P54071	C113	reversible
BF7	Pyruvate metabolism and Citric Acid (TCA) cycle	2	P54071	C154	reversible
BF7	Pyruvate metabolism and Citric Acid (TCA) cycle	3	Q7TSQ8	C84	reversible
BF7	Pyruvate metabolism and Citric Acid (TCA) cycle	4	Q8K2B3	C238	reversible
BF7	Pyruvate metabolism and Citric Acid (TCA) cycle	4	Q8K2B3	C266	reversible
BF7	Pyruvate metabolism and Citric Acid (TCA) cycle	4	Q8K2B3	C475	reversible
BF7	Pyruvate metabolism and Citric Acid (TCA) cycle	4	Q8K2B3	C536	reversible
BF7	Pyruvate metabolism and Citric Acid (TCA) cycle	4	Q8K2B3	C654	reversible
BF7	Pyruvate metabolism and Citric Acid (TCA) cycle	1	P70404	C148	reversible
BF7	Pyruvate metabolism and Citric Acid (TCA) cycle	1	P70404	C235	reversible
BF7	Pyruvate metabolism and Citric Acid (TCA) cycle	1	P70404	C236	reversible
BF7	Pyruvate metabolism and Citric Acid (TCA) cycle	2	P00342	C163	so3h

BF7	Pyruvate metabolism and Citric Acid (TCA) cycle	3	P54071	C418	reversible
BF7	Pyruvate metabolism and Citric Acid (TCA) cycle	4	Q9Z2I9	C152	reversible
BF7	Pyruvate metabolism and Citric Acid (TCA) cycle	4	Q9Z2I9	C158	reversible
BF7	Pyruvate metabolism and Citric Acid (TCA) cycle	4	Q9Z2I9	C430	reversible
BF7	Pyruvate metabolism and Citric Acid (TCA) cycle	1	Q8K2B3	C189	reversible
BF7	Pyruvate metabolism and Citric Acid (TCA) cycle	1	Q8K2B3	C467	reversible
BF7	Pyruvate metabolism and Citric Acid (TCA) cycle	2	Q8K2B3	C191	reversible
BF7	Pyruvate metabolism and Citric Acid (TCA) cycle	4	Q9CZB0	C107	reversible
BF7	Pyruvate metabolism and Citric Acid (TCA) cycle	4	Q99KI0	C385	reversible
BF7	Pyruvate metabolism and Citric Acid (TCA) cycle	4	Q9CZU6	C101	reversible
BF7	Pyruvate metabolism and Citric Acid (TCA) cycle	4	P08249	C212	reversible
BF7	Pyruvate metabolism and Citric Acid (TCA) cycle	4	P08249	C275	reversible
BF7	Pyruvate metabolism and Citric Acid (TCA) cycle	4	P08249	C285	reversible
BF7	Pyruvate metabolism and Citric Acid (TCA) cycle	4	P08249	C89	reversible
BF7	Pyruvate metabolism and Citric Acid (TCA) cycle	4	P08249	C93	reversible
BF7	Pyruvate metabolism and Citric Acid (TCA) cycle	4	P54071	C308	reversible
BF7	Pyruvate metabolism and Citric Acid (TCA) cycle	4	P54071	C402	reversible
BF8	Respiratory electron transport	1	Q91VD9	C554	reversible
BF8	Respiratory electron transport	1	Q91VD9	C179	so3h
BF8	Respiratory electron transport	1	Q91VD9	C176	so2h
BF8	Respiratory electron transport	1	Q91VD9	C182	so3h
BF8	Respiratory electron transport	2	Q921G7	C588	reversible
BF8	Respiratory electron transport	3	Q91VD9	C78	reversible
BF8	Respiratory electron transport	4	Q91VD9	C367	reversible
BF8	Respiratory electron transport	4	Q91VD9	C75	reversible
BF8	Respiratory electron transport	4	Q91VD9	C92	reversible
BF8	Respiratory electron transport	5	Q921G7	C386	reversible
BF8	Respiratory electron transport	1	Q921G7	C560	so3h
BF8	Respiratory electron transport	2	Q9DCJ5	C78	reversible
BF8	Respiratory electron transport	3	Q8JZN5	C275	reversible
BF8	Respiratory electron transport	3	Q8JZN5	C617	reversible
BF8	Respiratory electron transport	4	Q921G7	C117	reversible

BF8	Respiratory electron transport	4	Q921G7	C265	reversible
BF8	Respiratory electron transport	5	P52503	C104	reversible
BF8	Respiratory electron transport	1	Q7TMF3	C92	reversible
BF8	Respiratory electron transport	2	Q9CR61	C59	reversible
BF8	Respiratory electron transport	4	Q9D8B4	C18	reversible
BF8	Respiratory electron transport	4	Q9D8B4	C95	reversible
BF8	Respiratory electron transport	5	Q9CQJ8	C42	reversible
BF8	Respiratory electron transport	1	Q9DC70	C194	reversible
BF8	Respiratory electron transport	2	P52503	C79	so3h
BF8	Respiratory electron transport	4	Q9DCS9	C125	reversible
BF8	Respiratory electron transport	4	Q9DCS9	C77	reversible
BF8	Respiratory electron transport	5	Q91YT0	C255	reversible
BF8	Respiratory electron transport	1	Q91YT0	C238	reversible
BF8	Respiratory electron transport	1	Q91YT0	C255	so3h
BF8	Respiratory electron transport	2	Q8JZN5	C331	reversible
BF8	Respiratory electron transport	4	Q9DCJ5	C110	reversible
BF8	Respiratory electron transport	4	Q9DCJ5	C36	reversible
BF8	Respiratory electron transport	4	Q9DCJ5	C66	reversible
BF8	Respiratory electron transport	2	Q91YT0	C206	so3h
BF8	Respiratory electron transport	4	Q9CR61	C80	reversible
BF8	Respiratory electron transport	4	Q99LC5	C109	reversible
BF8	Respiratory electron transport	4	Q99LC5	C155	reversible
BF8	Respiratory electron transport	4	Q99LC5	C53	reversible
BF8	Respiratory electron transport	4	Q99LC5	C68	reversible
BF8	Respiratory electron transport	4	Q99LY9	C43	reversible
BF8	Respiratory electron transport	4	Q99LY9	C66	reversible
BF8	Respiratory electron transport	4	Q99LC3	C183	reversible
BF8	Respiratory electron transport	4	Q99LC3	C67	reversible
BF8	Respiratory electron transport	4	P52503	C79	reversible
BF8	Respiratory electron transport	4	Q9DCW4	C66	reversible
BF8	Respiratory electron transport	4	Q9DCW4	C71	reversible
BF8	Respiratory electron transport	4	Q91YT0	C125	reversible
BF8	Respiratory electron transport	4	Q91YT0	C142	reversible
BF9	Branched-chain amino acid catabolism	1	Q9DBL1	C261	reversible
BF9	Branched-chain amino acid catabolism	2	O35855	C346	reversible
BF9	Branched-chain amino acid catabolism	3	O35855	C343	reversible
BF9	Branched-chain amino acid catabolism	3	O35855	C93	reversible
BF9	Branched-chain amino acid catabolism	4	Q9DBL1	C234	reversible
BF9	Branched-chain amino acid catabolism	5	O35855	C136	reversible

BF9	Branched-chain amino acid catabolism	1	O35855	C148	reversible
BF9	Branched-chain amino acid catabolism	2	Q99MR8	C186	reversible
BF9	Branched-chain amino acid catabolism	3	Q8QZS1	C44	reversible
BF9	Branched-chain amino acid catabolism	4	Q99MR8	C450	reversible
BF9	Branched-chain amino acid catabolism	4	Q99MR8	C591	reversible
BF9	Branched-chain amino acid catabolism	5	O08749	C477	reversible
BF9	Branched-chain amino acid catabolism	1	Q9D7B6	C281	reversible
BF9	Branched-chain amino acid catabolism	2	Q3ULD5	C267	reversible
BF9	Branched-chain amino acid catabolism	2	Q3ULD5	C392	reversible
BF9	Branched-chain amino acid catabolism	4	Q9D7B6	C215	reversible
BF9	Branched-chain amino acid catabolism	4	Q9D7B6	C249	reversible
BF9	Branched-chain amino acid catabolism	1	Q8BH95	C225	reversible
BF9	Branched-chain amino acid catabolism	2	P53395	C333	reversible
BF9	Branched-chain amino acid catabolism	4	Q3ULD5	C167	reversible
BF9	Branched-chain amino acid catabolism	4	Q3ULD5	C391	reversible
BF9	Branched-chain amino acid catabolism	1	Q6P3A8	C226	reversible
BF9	Branched-chain amino acid catabolism	2	Q6P3A8	C297	reversible
BF9	Branched-chain amino acid catabolism	4	O08749	C80	reversible
BF9	Branched-chain amino acid catabolism	4	O08749	C85	reversible
BF9	Branched-chain amino acid catabolism	1	Q8QZS1	C335	reversible
BF9	Branched-chain amino acid catabolism	2	Q9JHI5	C134	reversible
BF9	Branched-chain amino acid catabolism	4	Q9JHI5	C252	reversible
BF9	Branched-chain amino acid catabolism	4	Q9JHI5	C348	reversible
BF9	Branched-chain amino acid catabolism	2	P50136	C194	reversible
BF9	Branched-chain amino acid catabolism	4	Q8QZS1	C94	reversible
BF9	Branched-chain amino acid catabolism	4	Q8QZT1	C116	reversible
BF9	Branched-chain amino acid catabolism	4	Q8QZT1	C193	reversible
BF9	Branched-chain amino acid catabolism	4	Q9EQ20	C86	reversible
BF10	Fatty acid metabolism	1	Q9CQR4	C74	reversible
BF10	Fatty acid metabolism	2	P34914	C120	reversible
BF10	Fatty acid metabolism	3	P34914	C521	reversible
BF10	Fatty acid metabolism	4	P34914	C230	reversible
BF10	Fatty acid metabolism	5	O70325	C102	reversible
BF10	Fatty acid metabolism	1	Q07417	C151	reversible
BF10	Fatty acid metabolism	1	Q07417	C109	reversible
BF10	Fatty acid metabolism	2	Q9WUR2	C279	reversible
BF10	Fatty acid metabolism	3	Q99MN9	C518	reversible
BF10	Fatty acid metabolism	3	Q99MN9	C367	reversible
BF10	Fatty acid metabolism	3	Q99MN9	C519	reversible

BF10	Fatty acid metabolism	4	Q61425	C201	reversible
BF10	Fatty acid metabolism	5	Q8BWT1	C103	reversible
BF10	Fatty acid metabolism	5	Q8BWT1	C287	reversible
BF10	Fatty acid metabolism	1	Q8BWT1	C107	reversible
BF10	Fatty acid metabolism	1	Q8BWT1	C382	reversible
BF10	Fatty acid metabolism	2	P16332	C469	reversible
BF10	Fatty acid metabolism	3	O70325	C16	so2h
BF10	Fatty acid metabolism	4	Q924X2	C448	reversible
BF10	Fatty acid metabolism	4	Q924X2	C526	reversible
BF10	Fatty acid metabolism	5	Q9Z2Z6	C58	reversible
BF10	Fatty acid metabolism	1	Q8BH95	C225	reversible
BF10	Fatty acid metabolism	2	Q61425	C99	reversible
BF10	Fatty acid metabolism	3	Q07417	C289	reversible
BF10	Fatty acid metabolism	4	Q9Z2Z6	C155	reversible
BF10	Fatty acid metabolism	5	Q9CQ62	C86	reversible
BF10	Fatty acid metabolism	1	Q8C7H1	C97	reversible
BF10	Fatty acid metabolism	2	O55137	C412	reversible
BF10	Fatty acid metabolism	3	P51174	C342	reversible
BF10	Fatty acid metabolism	4	Q8BMS1	C747	reversible
BF10	Fatty acid metabolism	5	P41216	C242	reversible
BF10	Fatty acid metabolism	1	Q99J39	C31	so3h
BF10	Fatty acid metabolism	1	Q99J39	C447	reversible
BF10	Fatty acid metabolism	2	Q921H8	C381	reversible
BF10	Fatty acid metabolism	3	Q924X2	C155	reversible
BF10	Fatty acid metabolism	4	P41216	C510	reversible
BF10	Fatty acid metabolism	5	P50544	C423	reversible
BF10	Fatty acid metabolism	1	P41216	C55	reversible
BF10	Fatty acid metabolism	2	Q8BWT1	C92	reversible
BF10	Fatty acid metabolism	3	Q99J39	C359	reversible
BF10	Fatty acid metabolism	4	Q80XL6	C635	so3h
BF10	Fatty acid metabolism	2	P52825	C512	reversible
BF10	Fatty acid metabolism	2	P52825	C84	reversible
BF10	Fatty acid metabolism	3	Q99P30	C223	reversible
BF10	Fatty acid metabolism	4	P50544	C478	reversible
BF10	Fatty acid metabolism	2	P41216	C626	reversible
BF10	Fatty acid metabolism	3	Q8BMS1	C470	reversible
BF10	Fatty acid metabolism	2	P47934	C449	reversible
BF10	Fatty acid metabolism	3	P41216	C133	reversible
BF10	Fatty acid metabolism	2	P50544	C216	reversible

BF10	Fatty acid metabolism	2	P50544	C238	reversible
BF10	Fatty acid metabolism	2	P50544	C604	reversible
BF10	Fatty acid metabolism	3	Q9R0H0	C199	reversible
